# Metallomimetic C–F Activation Catalysis by Simple Phosphines

**DOI:** 10.1021/jacs.3c10614

**Published:** 2024-01-11

**Authors:** Sara Bonfante, Christian Lorber, Jason M. Lynam, Antoine Simonneau, John M. Slattery

**Affiliations:** † Department of Chemistry, University of York, Heslington, York YO10 5DD, U.K.; ‡ LCC−CNRS, Université de Toulouse, CNRS, UPS, 205 Route de Narbonne, BP44099, Toulouse Cedex 4 F-31077, France

## Abstract

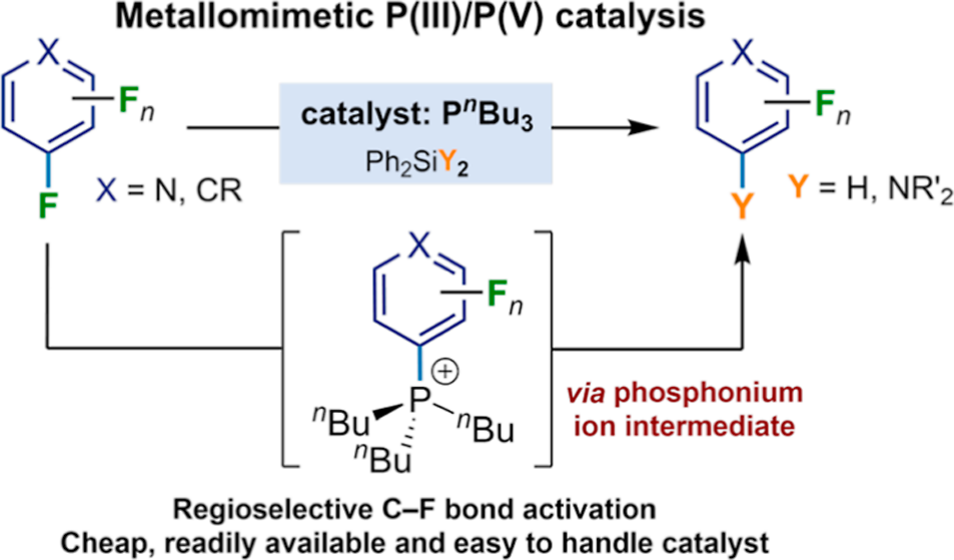

Delivering metallomimetic reactivity from simple p-block compounds is highly desirable in the search to replace expensive, scarce precious metals by cheap and abundant elements in catalysis. This contribution demonstrates that metallomimetic catalysis, involving facile redox cycling between the P(III) and P(V) oxidation states, is possible using only simple, cheap, and readily available trialkylphosphines without the need to enforce unusual geometries at phosphorus or use external oxidizing/reducing agents. Hydrodefluorination and aminodefluorination of a range of fluoroarenes was realized with good to very good yields under mild conditions. Experimental and computational mechanistic studies show that the phosphines undergo oxidative addition of the fluoroaromatic substrate via a Meisenheimer-like transition state to form a fluorophosphorane. This undergoes a pseudotransmetalation step with a silane, via initial fluoride transfer from P to Si, to give experimentally observed phosphonium ions. Hydride transfer from a hydridosilicate counterion then leads to a hydridophosphorane, which undergoes reductive elimination of the product to reform the phosphine catalyst. This behavior is analogous to many classical transition-metal-catalyzed reactions and so is a rare example of both functional and mechanistically metallomimetic behavior in catalysis by a main-group element system. Crucially, the reagents used are cheap, readily available commercially, and easy to handle, making these reactions a realistic prospect in a wide range of academic and industrial settings.

## Introduction

There has been a significant amount of interest in recent years in the activation and functionalization of small molecules and strong bonds by main-group-element compounds acting in similar ways to transition metals.^1−4^ In addition to fundamental interest and the development of novel reactivity, this has been driven, in part, by a desire to find more sustainable alternatives to the low-abundant, expensive, and potentially toxic precious metals that are commonly used in homogeneous catalysis. A key foundation of this work is the concept of metallomimetic behavior,^5,6^ where main-group-element species can be prompted to display analogous reactivity to transition metals, e.g., through the formation of unusual bonding modes, oxidation states, coordination geometries, and of course, the huge efforts around frustrated Lewis pairs.^7−15^ Of all the transition-metal like reactivity, the ability to undergo reversible two-electron redox processes, such as oxidative addition (OA) and reductive elimination (RE), is at the core of many traditional homogeneous catalytic cycles. Similarly facile redox cycling in main-group elements is much rarer, although promoting OA and RE processes have been a highly active area of study in recent years.^16^ A key challenge with many main-group species is that there are often larger differences in stability between different oxidation states when compared to transition metals, which results in either rapid OA or RE, but then a much more challenging reverse process that renders catalysis difficult. However, among the main group elements the pnictogens stand out in this regard, often allowing access to stable species in different oxidation states, and as such they are ideal candidates for the development of metallomimetic reactivity and catalysis.^17^ A key design concept that has led to observations of redox-cycling within these systems is the introduction of geometrical constraints around the pnictogen atoms, moving them away from their preferred pyramidal to more planar geometries, resulting in changes in orbital energies and reactivity.^18−24^ Pincer ligands are often used for this purpose, promoting ambiphilic behavior and unusual reactivity of the pnictogen,^25−34^ although metal–ligand cooperativity is also frequently observed in these systems.^35−43^

**Scheme 1:**
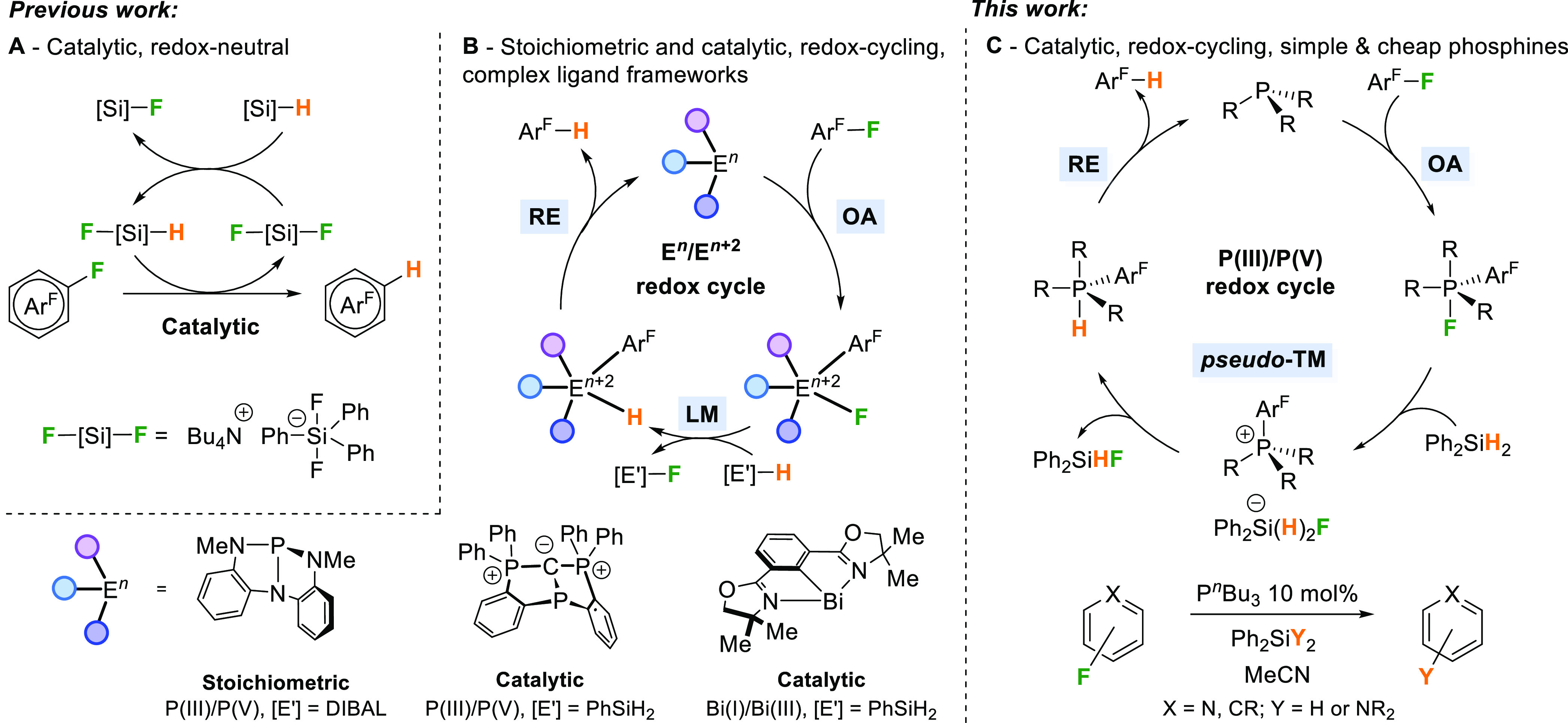
Comparison of Recent Aromatic Hydrodefluorination (HDF) Reactions Promoted or Catalysed by Main Group Compounds Through Redox-Neutral (A) or Redox-Cycling (B) Pathways and the Present Work (C)

Key reactions in which main-group systems have been studied are catalytic hydrodefluorination (HDF) and related defluorination processes (Scheme 1). These are important approaches to prepare complex fluorinated molecules, which have applications in pharmaceuticals,^44,45^ agrochemicals,^46^ and materials chemistry,^47^ from readily available polyfluorinated precursors.^48^ Transition-metal-based C–F functionalization is relatively well established,^49−52^ and a range of main-group systems have also been explored for HDF.^53,54^ These include non-redox systems such as strong Lewis acids,^55^ including electron-deficient phosphonium ions,^56−58^ and FLPs,^59^ which allow HDF of aliphatic C–F bonds, tetrabutylammonium triphenyldifluorosilicate (TBAT),^60^ NaBH_4_,^61^ and diazaphospholenes,^62,63^ which can catalyze HDF of aromatic C–F bonds and trifluoromethylalkenes. Important recent studies have elegantly demonstrated that geometrically constrained pnictogen systems can promote HDF, either through a series of stoichiometric steps or catalytically, via P(III)/P(V) or Bi(I)/Bi(III) redox cycling. Crucially, the three key processes of OA, ligand metathesis (LM)/transmetalation (TM), and RE that underpin many transition-metal catalytic mechanisms were seen in these systems (Scheme 1).^21,23,24^ These are remarkable demonstrations that main-group systems can mechanistically mimic the key steps in transition-metal catalysis. However, complex ligand architectures were used, and these were proposed to play an important role in the observed reactivity through geometrically constraining the pnictogen. Inspired by reports of stoichiometric reactivity of simple phosphines with fluoroarenes, we set out to explore whether geometric constraints are a necessary prerequisite of redox-cycling in HDF using pnictogen catalysts.^64−66^ We now report that a simple catalyst system constituted from commercially available alkyl phosphines and silanes is able to perform the HDF reaction on a range of aromatic substrates.

## Results and Discussion

Initial investigations into the catalytic HDF of pentafluoropyridine (**1**) by simple phosphines probed whether **1** and PhSiH_3_ could form 2,3,5,6-tetrafluoropyridine (**2**) in the presence of catalytic P^*i*^Pr_3_ (10 mol %) under similar reaction conditions to those used by Dobrovetsky and co-workers (*o*-C_6_H_4_F_2_ at 60 °C, Table 1, entry 1) with a geometrically constrained phosphine catalyst.^21^ After 18 h, we were pleased to find that **2** was formed, albeit in only modest, 26%, yield. The ^19^F NMR spectra of the reaction mixture showed peaks at δ = −92.9 and −141.5 ppm indicative of the *ortho*- and *meta*-fluorine environments of **2**, respectively. Unreacted **1** could also clearly be seen (65%) and 9% of the product of reductive coupling of **1**, perfluoro 4,4′-bipyridine (**3**) was also formed, identified through ^19^F NMR signals at δ = −95.0 and −137.2 ppm. A control experiment under the same conditions showed that **1** and PhSiH_3_ did not form **2** in the absence of a phosphine (see Supporting Information for details). These results demonstrate for the first time that catalytic HDF is possible using only a simple trialkylphosphine as the catalyst. However, the reaction was significantly slower than that reported by Dobrovetsky and co-workers, who observed 95% yield of **2** in only 3 h at 80 °C with a geometrically constrained P(III) catalyst. The simple nature of the phosphine and silane in our system allowed for straightforward reaction optimization with a range of potential catalysts and hydride sources. Our focus here was not only on rate and selectivity, but also to make use of the simplest, most widely available and cheapest catalysts and supporting reagents in order to ensure that reactions are widely applicable.

**Table 1:**
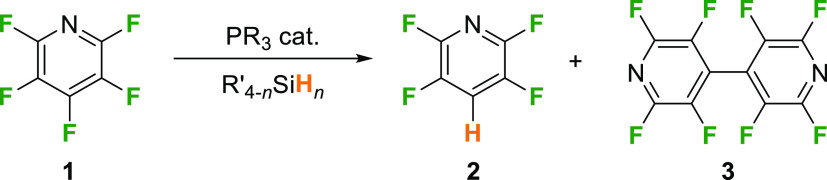
Reaction Optimization for HDF of Pentafluoropyridine (**1**)^a^

entry	PR_3_	PR_3_ mol %	silane	silane equiv.	solvent	temp./°C	time/h	yield of **2**/%
1	P^*i*^Pr_3_	10	PhSiH_3_	1	*o*-C_6_H_4_F_2_	60	18	26
2	P^*i*^Pr_3_	10	PhSiH_3_	1	MeCN	60	44	76
3	P^*i*^Pr_3_	10	Ph_2_SiH_2_	1	MeCN	20	18	84
4	P^*i*^Pr_3_	10	Ph_3_SiH	2	MeCN	20	18	2
5	P^*n*^Bu_3_	10	PhSiH_3_	1	MeCN	60	2	100
6	P^*n*^Bu_3_	10	Ph_2_SiH_2_	1	MeCN	20	0.33	93
7	P^*n*^Bu_3_	10	Ph_3_SiH	2	MeCN	20	18	6
8	P^*n*^Bu_3_	10	Ph_2_SiH_2_	0.55	MeCN	20	3	87
9	P^*n*^Bu_3_	5	Ph_2_SiH_2_	1	MeCN	20	1	93
10	P^*n*^Bu_3_	1	Ph_2_SiH_2_	1	MeCN	20	18	83

a NMR yields determined by integration of ^19^F NMR spectra using trifluorotoluene as an internal standard (see Supporting Information).

## Reaction Optimization

A range of conditions were tested to explore phosphine, silane, and solvent effects on the HDF of **1** (Table 1). Changing the solvent from *o*-C_6_H_4_F_2_ to MeCN had a significant, positive effect on the reaction, and gave 67% of **2** after 18 h at 60 °C and 76% after 44 h (Table 1, entry 2) alongside a small amount of **3** (4%), trace unreacted **1** and small quantities of other unidentified fluorinated products. Changing the silane to Ph_2_SiH_2_ significantly increased the reaction rate (Table 1, entry 3), allowing a lower temperature to be used and giving 84% of **2** after only 18 h at 20 °C, in addition to a small amount of **3** (7%). Further H/Ph substitution on the silane, however, only led to trace amounts of **2** when Ph_3_SiH was used (Table 1, entry 4) with most of the starting material **1** left unreacted, alongside the formation of 8% of **3**.

Another substantial improvement in catalytic performance was found when P^*i*^Pr_3_ was substituted for P^*n*^Bu_3_, with the silanes showing the same trend for the two phosphines (Table 1 entries 5–7). The combination of P^*n*^Bu_3_ and Ph_2_SiH_2_ provided optimal reaction conditions allowing HDF of **1** to give **2** in excellent yield (93%) in only 20 min at 20 °C (Table 1 entry 6). This is among the best catalytic aromatic HDF performance so far reported by a main-group-element catalyst.^21,24,60,62^ Pleasingly, this combination of phosphine and silane were also the cheapest^67^ and most readily available that were tested. In addition, P^*n*^Bu_3_ and Ph_2_SiH_2_ gave HDF that was selective for **2** under these conditions, no other HDF products were observed, e.g., other regioisomers and/or multiple HDFs.

Experiments to reduce the phosphine and silane loadings (Table 1, entries 8–10) showed that using a close to stoichiometric (in terms of hydride equivalents) amount of Ph_2_SiH_2_ still allowed good conversion to **2** (87%), although the reaction was slower and a small amount of **3** (2%) was formed alongside the desired product (Table 1, entry 8). Reduction of the phosphine loading to 5 mol % was still very effective, albeit slower (93% **2** after 1 h), and at 1 mol % selective formation of **2** was still possible, but reaction times were much longer (83% yield after 18 h) and some unreacted **1** (11%) was present after this time. Given the low cost of both P^*n*^Bu_3_ and Ph_2_SiH_2_ it was decided to use the reaction conditions in entry 6 in Table 1, for further studies, as these led to fast reactions without a significant increase in cost.

## Substrate Scope

**Scheme 2:**
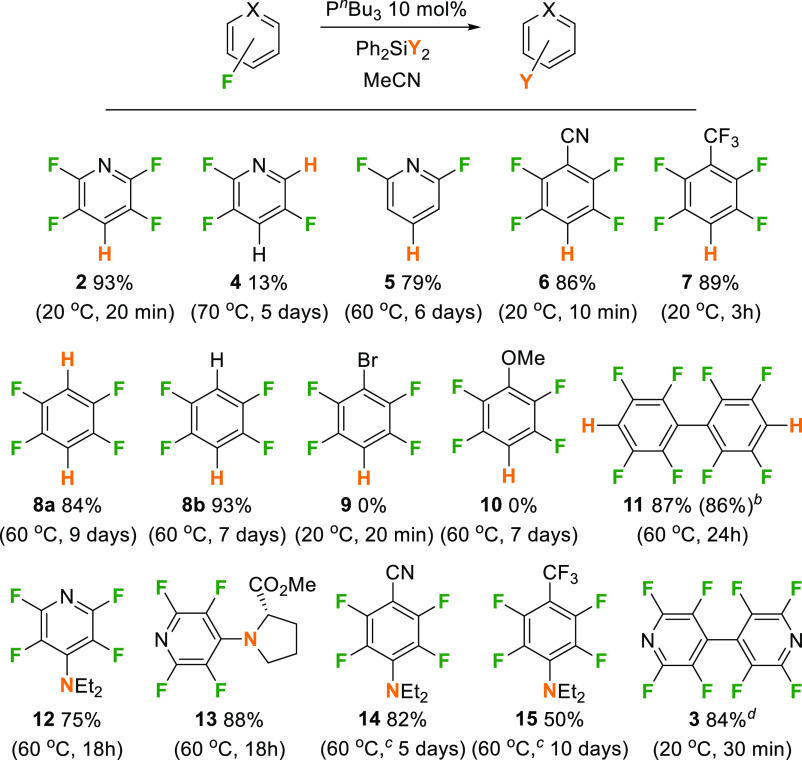
Results of Substrate Scope Studies Ph_2_SiH_2_ was used for all HDF reactions and Ph_2_Si(Cl)(NR_2_) were used for aminodefluorination reactions. Isolated yield after flash column chromatography shown in parentheses. Reactions were stirred at 60 °C for 4 days, followed by the remaining period at 80 °C. Stoichiometric reaction between P^*i*^Pr_3_ and **1** in MeCN (other phosphines behave similarly).

The substrate scope was explored for HDF of a range of other fluoroarenes and heterocycles (Scheme 2, HDF products **2–11**). In addition, it was possible to determine conditions for the selective reductive coupling of **1** to **3** and to explore preliminary aminodefluorination reactions (products **12–15**) to allow C–F functionalization by amines.

Exploration of the degree of fluorination of fluoropyridines and the regioselectivity of the HDF reaction gave insights into the reaction mechanism and synthetic possibilities. With 2,3,5,6-tetrafluoropyridine as a substrate, it was possible to observe the formation of **4**, but in low yield and with much more forcing conditions (13% after 5 days at 70 °C). ^19^F NMR spectroscopy clearly showed the presence of **4**, with peaks at δ = −92.3, −129.0, and −136.5 ppm, unreacted tetrafluoropyridine (25%) and some additional low-intensity signals that were consistent with other tri- and difluoropyridine isomers. This explains the selectivity of the HDF conditions in Table 1, entry 6, as a second HDF at the less activated *ortho*-position of the pyridine is substantially slower than the initial reaction at the *para*-position of **1**. The presence of other tri- and difluoropyridines suggests that the rate of HDF at the *ortho*- and *meta*-positions is similar to each other and slower than the rate of HDF at the *para*-position. Reducing the degree of fluorination of the substrate but maintaining a C–F bond at the most reactive *para*-position also allowed HDF and formation of **5** (79% yield), but the reaction was relatively slow and a small amount of unreacted 2,4,6-trifluoropyridine (5%) was present, along with some 2,4-difluoropyridine (14%), which results from *ortho*-HDF, after 6 days. It is clear that more electron-poor heteroaromatics lead to faster reactions, which is consistent with the mechanistic proposal (see below).

The catalytic HDF reaction is not limited to pyridines. Reaction of other electron-poor aromatics, such as pentafluorobenzonitrile and perfluorotoluene, led to rapid formation of **6** and **7** in very good yields (86 and 89%, respectively). Simple fluorobenzenes showed trends similar to those seen with the fluoropyridines, with hexafluorobenzene undergoing double HDF to ultimately form tetrafluorobenzene (Scheme 2, **8a**). Formation of **8a** can be seen after 2 h, with the amount increasing after 18 h, but interestingly only trace amounts of pentafluorobenzene were seen in the ^19^F NMR spectra at these time periods. It appears that the rates of HDF of hexa- and pentafluorobenzene are similar and overall significantly slower than perfluorotoluene. This was confirmed by direct HDF of pentafluorobenzene (Scheme 2, **8b**), which occurred on a similar time scale to **8a** under similar conditions. The ultimate yields of 1,2,4,5-tetrafluorobenzene were very good in both cases (84 and 93%, respectively). Perfluorobiphenyl also converted effectively to the doubly hydrodefluorinated product **11**, in 87% yield after 24 h at 60 °C. During the course of the reaction the monohydrodefluorinated product was seen (39%) in the ^19^F NMR spectra after 1 h with signals at δ = −139.5, −152.6, and −162.9 ppm, alongside **11** (15%) and unreacted perfluorobiphenyl (42%).

Introduction of an electron-donating substituent, in pentafluoroanisole, was not tolerated in this system, with only traces of hydrodefluorinated products observed after 7 days at 60 °C alongside the majority of the starting material remaining unreacted. The use of bromopentafluorobenzene as a substrate also resulted in no conversion to the hydrodefluorinated product **9** after 20 min at 20 °C. However, in this case a small amount of pentafluorobenzene (3%) was formed and a new species was seen in the ^19^F NMR spectrum at δ = −128.4, −142.9, and −158.3 ppm (11%). This was associated with a signal in the ^31^P NMR spectrum at 36.6 ppm, which was the major phosphorus-containing product. Addition of a further 0.9 equiv of P^*n*^Bu_3_ significantly increased the intensity of the signals from this species in the ^19^F and ^31^P NMR spectra (recorded after 30 min at 20 °C), along with the consumption of the majority of the bromopentafluorobenzene. This new species was assigned as the phosphonium salt [P(C_6_F_5_)(^*n*^Bu)_3_]Br (**[16]Br**), which results from C–Br rather than C–F activation of the fluoroarene. At this point, additional formation of pentafluorobenzene was also seen (21%) and after 18 h at 20 °C, this had increased slightly to 35%. For this substrate, it appears that hydrodebromination is favored over HDF, although higher phosphine loadings are required, as the system appears to rest as the relatively unreactive salt **[16]Br**, which has implications in terms of the mechanistic proposal (discussed below).

Extending the catalytic methodology to other C–F functionalization reactions was also explored. Preliminary studies investigated C–F amination by silylamides in the presence of catalytic P^*n*^Bu_3_.^21^ Thus, when **1** was reacted with Ph_2_Si(Cl)(NEt_2_) with 10 mol % of P^*n*^Bu_3_ at 60 °C for 18 h a good yield (75%) of the aminated product **12** was seen, alongside a small amount of unreacted **1** (10%). This was characterized by signals in the ^19^F NMR at δ = −96.7 and −157.0 ppm, in the ^1^H NMR at δ = 3.43 (qt, ^3^*J*_HH_ = 7 Hz, and ^5^*J*_HF_ = 2 Hz) and 1.22 (t, ^3^*J*_HH_ = 7 Hz) ppm and by an M^+^ ion of 222.07810 *m*/*z* (2.85 ppm deviation from theoretical) in the EI-MS. Unlike most of the HDF reactions described above, which were generally very selective, under aminodefluorination conditions an additional product (10%) was observed in the ^19^F NMR at δ = −89.0, −125.2, and −151.3 ppm. This was associated with a new signal in the ^31^P NMR spectrum at δ = 36.4 ppm, whose chemical shift was typical of a phosphonium salt, which represented the major phosphorus-containing species. Extending this reaction to more complex amines also proved possible, and we were delighted to see that the reaction of **1** with Ph_2_Si(Cl)(pro) (where pro is *l*-proline methyl ester) gave a very good yield of **13** (88%) alongside a small amount of unreacted **1**. The ^19^F NMR spectrum of **13** was similar to that of **12**, with ^19^F signals at δ = −97.0 and −160.1 ppm. The ^1^H NMR spectrum was consistent with retention of the proline methyl ester moiety (see Supporting Information) and EI-MS confirmed the accurate mass of the [M]^+^ ion at 278.06880 *m*/*z* (5.41 ppm deviation from theoretical). This demonstrated that potentially sensitive functional groups are tolerated by this catalytic aminodefluorination reaction and that it may find application in the preparation of highly functionalized, fluorinated amines, e.g., in pharmaceutical or agrochemical synthesis. Extending the fluoroarene substrates beyond **1** also proved possible, with both pentafluorobenzonitrile and perfluorotoluene also reacting with Ph_2_Si(Cl)(NEt_2_) in the presence of 10 mol % P^*n*^Bu_3_ to give **14** and **15** in 82 and 50% yield, respectively. However, these substrates proved more sluggish under the reaction conditions optimized for HDF and further optimization is desirable in future studies to extend the aminodefluorination reactions beyond these preliminary results.

Significant amounts of reductive coupling of pentafluoropyridine to form **3** were not seen under the catalytic conditions described above. In an attempt to optimize this simple phosphine system for the preparation of **3**, stoichiometric conditions for this reaction were explored. Thus, when P^*i*^Pr_3_ (1 equiv) and **1** were allowed to react in MeCN at 20 °C for 30 min, **3** was formed in 84% yield, alongside an equivalent amount of PF_2_(^*i*^Pr)_3_ and a small amount of unreacted **1** (16%). We have observed similar reactivity with other phosphines under stoichiometric conditions and analogous reactivity has been described before for P(NEt_2_)_3_.^68^ Although this is stoichiometric in phosphine, rather than catalytic, given the generally low cost of the phosphines used and the simple and mild reaction conditions, it may prove a useful route to **3**.

Overall, it is clear that both HDF and aminodefluorination are feasible for a range of substrates using only a simple, readily commercially available phosphine as the catalysts. The product yields and reaction times are among the best observed for main-group catalysts for highly electron-poor fluoroarenes. The performance of this system is often better than that in related geometrically constrained P(III)/P(V) systems, although the silane/solvent used there is different, suggesting that geometric constraints are not required for useable reactivity. However, this is an effective design principle for some substrates/reactions. The key features of the catalytic system described here, however, are simplicity and cost. As all reagents are commercially available and easy to handle, there is no barrier to entry into these catalytic reactions, broadening their potential applicability in a range of settings.

## Mechanistic Studies

**Scheme 3:**
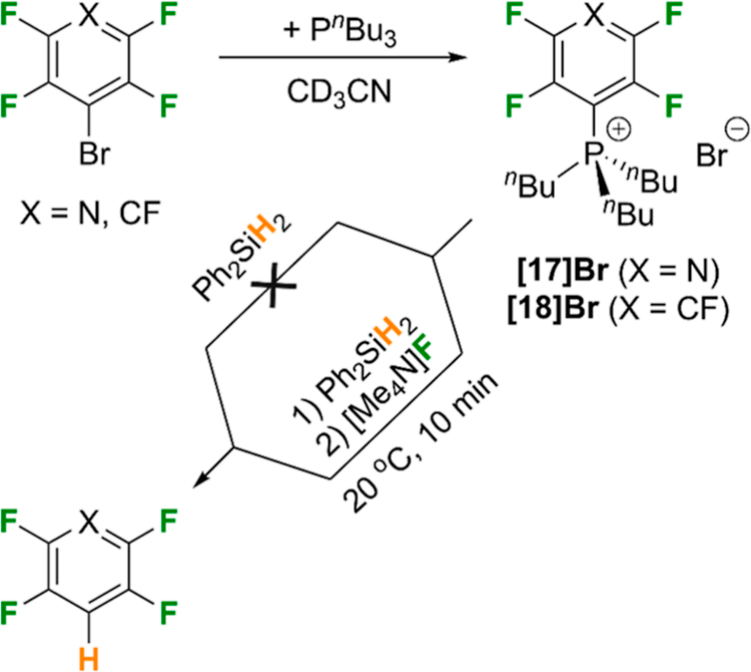
Preparation of Phosphonium Ions Proposed to be Intermediates in the Catalytic Reactions and their Reactivity Toward Silanes in the Presence, and Absence, of a Fluoride Source

Experimental and computational mechanistic studies were undertaken to probe the pathways underpinning the HDF reaction. Under the optimized reaction conditions for the HDF of **1** (Table 1, entry 6) the reaction occurred too quickly to observe any intermediates. However, for slower reactions it was possible to gain insights through the observation of intermediate phosphorus-containing species. When **1** was reacted with PhSiH_3_, with 10 mol % P^*n*^Bu_3_ in MeCN at 20 °C, after 1 h the ^31^P NMR spectrum showed very clean conversion of P^*n*^Bu_3_ into a new species with an apparent septet resonance at δ = 38.1 ppm (*J* = ca. 5 Hz). This was accompanied by new complex multiplet signals in the ^19^F NMR spectrum at δ = −88.5 and −130.3 ppm. The only other resonances present in the ^19^F NMR were from unreacted **1**, the HDF product **2** and the fluorosilane produced by H/F exchange. The resonance at δ = 38.1 ppm in the ^31^P NMR spectrum is characteristic of a phosphonium cation and was therefore assigned to the [(C_5_F_4_N)P^*n*^Bu_3_]^+^ ion, **[17]**^**+**^. This was confirmed by independent preparation of [(C_5_F_4_N)P^*n*^Bu_3_]Br (**[17]Br**) by reaction of P^*n*^Bu_3_ with 4-bromo-2,3,5,6-tetrafluoropyridine (Scheme 3), which displayed virtually identical spectroscopic signals (^31^P δ = 37.9 ppm and ^19^F δ = −89.2 and 130.3 ppm) and coupling patterns. Similar addition of phosphines (e.g., PPh_3_) to activated pyridines (*N*-trifluoromethanesulfonylpyridinium salts) to give phosphonium salts has been observed previously.^69^ In addition, phosphonium salts derived from fluoroalkanes have been formed by FLP systems through aliphatic C–F activation by a strong Lewis acid (e.g., B(C_6_F_5_)_3_), followed by trapping of the resulting carbocation by PR_3_.^70,71^ Related fluorinated phosphonium salts, e.g., [(C_6_F_5_)_3_PF][B(C_6_F_5_)_4_], have also been used in the catalytic HDF of fluoroalkanes.^56−58^ However, these species are substantially more Lewis acidic than ions such as **[17]**^**+**^ and directly abstract fluoride from aliphatic C–F bonds, which is very different from the role of the phosphonium salts described here (see below). At the end of the catalytic reaction, when **1** was fully consumed, the signal for **[17]**^**+**^ disappeared and free phosphine was observed in the reaction mixture (^31^P δ = −32.1 ppm), suggesting its role as a resting state.

To investigate whether **[17]**^**+**^ was a relevant catalytic intermediate, Ph_2_SiH_2_ was added to the independently prepared sample of **[17]Br** in MeCN (Scheme 3). After 10 min at 20 °C, there was no change in the ^31^P or ^19^F NMR spectra. The mixture was heated to 40 °C overnight and similarly showed no reaction between **[17]Br** and the silane. However, on addition of 1 equiv. anhydrous [Me_4_N]F to the system at 20 °C, **2** was seen to form after only 10 min, alongside the formation of fluorosilane Ph_2_Si(F)_*n*_H_2–*n*_ and some unreacted **[17]**^**+**^. Over time, the amount of **2** increased as the concentration of **[17]**^**+**^ decreased. This suggested that **[17]**^**+**^ is an intermediate in the catalytic cycle but that it does not react directly with the silane to form the HDF product. We propose that the addition of fluoride to the silane to form a silicate anion, [Ph_2_Si(F)H_2_]^−^, is necessary to promote H^–^ transfer and liberation of the hydrodefluorinated product. Similar silane/silicate reactivity has been reported by Ogoshi and co-workers in metal-free HDF reactions using catalytic [Bu_4_N][Ph_3_SiF_2_] (TBAT).^60^ In their case, it was proposed that fluoride transfer from TBAT to silanes, such as Ph_3_SiH, generated hydrofluorosilicate ions that through ligand redistribution form transient dihydrosilicate ions, ultimately performing HDF of fluoroarenes. In the present work, it is not clear whether mono- or dihydrosilicate ions are involved in hydride transfer to phosphonium ions like **[17]**^**+**^. In order to assess the potential role of hydride transfer directly from a hydrosilicate anion to **1**, i.e., bypassing the phosphine-induced HDF, a control reaction was run where **1**, Ph_2_SiH_2_ and 10 mol % of [NMe_4_]F were allowed to react in MeCN at 20 °C. After 20 min some of the HDF product **2** was formed, but only in 19% yield, alongside mostly unreacted **1**. After 18 h, the same reaction had reached a yield of 64% of **2**. This contrasts strongly with the reactivity mediated by P^*n*^Bu_3_ (Table 1, entry 6) where 93% of **2** was formed under analogous conditions after only 20 min. Thus, it is possible to conclude that for **1** as a substrate, direct HDF of **1** by catalytic hydrosilicate anions, formed in situ by reaction of **1** with P^*n*^Bu_3_, is possible but significantly slower than HDF through a phosphine-mediated pathway.

An additional possibility that was explored to explain the need for fluoride to be present to initiate a reaction between **[17]**^**+**^ and Ph_2_SiH_2_ was fluoride addition to **[17]**^**+**^ to form a fluorophosphorane, which could aid Ph_2_SiH_2_ activation through an FLP-type mechanism similar to that proposed by Piers for Si–H activation.^72,73^ This would involve activation of the silane by the fluorophosphorane, acting as a Lewis base and donating a fluoride to the silane, alongside **[17]**^**+**^ acting as a Lewis acid to abstract a hydride from the silane in a concerted manner. DFT studies explored this, but a transition state associated with a concerted P–H/Si–F bond formation mechanism was not found. Instead, a two-step pathway, where fluoride transfer to Si to form a fluorosilicate anion occurs prior to hydride transfer to a phosphonium ion, was seen (see Supporting Information). Thus, we conclude from these computational studies and the fact that cations such as **[17]**^**+**^ are seen as intermediates in the ^31^P NMR spectra that fluorosilicate anion formation is a key step in the catalytic reaction mechanism.

Although the above mechanistic studies focused on intermediates in the HDF of **1**, similar observations were made in other reactions. For example, when perfluorotoluene was reacted with Ph_2_SiH_2_ at 20 °C in MeCN with P^*n*^Bu_3_ as the catalyst (10 mol %), after 10 min, the formation of a phosphonium ion was seen (^31^P δ = −38.1 ppm, apparent septet, ca. 4 Hz; ^19^F δ = −58.0 (t, ^4^*J*_FF_ = 18 Hz), −126.8 (m), −137.3 (m) ppm), which was assigned as [(C_6_F_4_CF_3_)P^*n*^Bu_3_]^+^ (**[18]**^**+**^). Also, when bromopentafluorobenzene was used as a substrate, the formation of the phosphonium salt [C_6_F_5_P^*n*^Bu_3_]Br (**[16]Br**) was observed under catalytic conditions, and its concentration could be increased by addition of an extra 0.9 equiv of P^*n*^Bu_3_, as described above and shown in Scheme 3. Under these conditions, some pentafluorobenzene was formed (35%) as a result of hydrodebromination, but the reaction was sluggish due to the slow reactivity of **[16]Br** with the silane. Addition of 1 equiv. [NMe_4_]F to this solution led to the disappearance of the peaks associated with **[16]Br** in the ^19^F NMR after 10 min at 20 °C, and to an increase in the amount of pentafluorobenzene (43%) along with the formation of some tetrafluorobenzene (16%). In addition, a new phosphonium ion was seen with ^31^P δ = 36.3 ppm and ^19^F δ = −129.7 and −135.7 ppm, which represented 43% of the fluoroarene-derived species. This was assigned as [C_6_F_4_HP^*n*^Bu_3_]^+^ (**[19]**^**+**^) and results from the C–F activation of pentafluorobenzene in the reaction mixture by the phosphine. After 18 h at 20 °C the system evolved, leading to a slightly reduced amount of pentafluorobenzene (41%), increased amount of tetrafluorobenzene (23%), and a similar amount of the phosphonium ion **[19]**^**+**^ (41%). Addition of fluoride to the system appears to have significantly increased the rate of hydrodebromination, which was then followed by HDF of the pentafluorobenzene product (cf. Scheme 2, **8b**). This is consistent with the proposed importance of the hydrosilicate anions in this system. Reaction of **[16]Br** with Ph_2_SiH_2_ to form [Ph_2_Si(Br)H_2_]^−^ would be significantly less favorable than fluoride transfer to the silane to form [Ph_2_Si(F)H_2_]^−^, therefore the concentration of the hydrosilicate ions remained low and hydrodebromination was slow until a source of fluoride was added to the system.

One final observation is important to note in a mechanistic context. As described above, stoichiometric reaction of PR_3_ with **1** in MeCN leads to rapid formation of reductive coupling product **3** (Scheme 2, **3**) alongside the formation of difluorophosphorane PR_3_F_2_. Dobrovetsky and co-workers observed similar reactivity when a geometrically constrained tetrafluoropyridyl-substituted fluorophosphorane was heated to 110 °C for 10 h in *o*-C_6_H_4_F_2_, i.e., **3** and a difluorophosphorane are formed.^21^ In our system, the related phosphorane R_3_P(C_5_F_4_N)F is not observed, as the reaction to form **3** is fast at room temperature. However, the observation of **3** in both systems suggests that a fluorophosphorane resulting from OA of **1** to PR_3_ is also a likely intermediate in the PR_3_-promoted reaction.

**Scheme 4:**
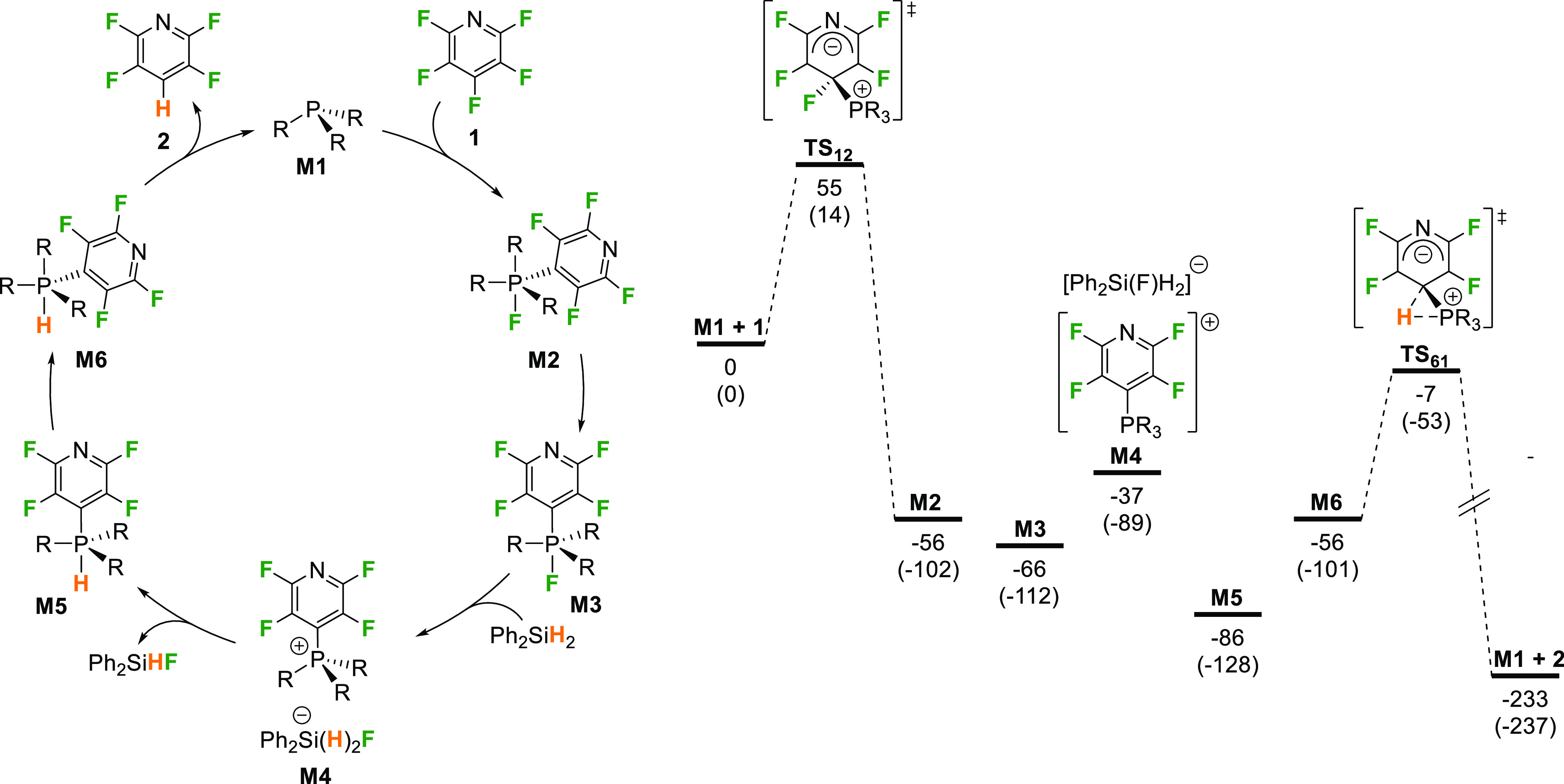
Proposed Catalytic Cycle and Computed Potential Energy Surface (PES) for R = Me All energies at the PBE0/def2-TZVP//BP86/SV(P) level in MeCN. Relative Gibbs energies (in kJ mol^–1^ at 298 K) shown outside brackets and relative enthalpies (in kJ mol^–1^ at 298 K) shown in brackets. See Supporting Information for details of solvent and dispersion corrections applied.

Looking at the catalytic reaction as a whole, the experimental mechanistic studies suggested that the phosphine catalyst adds to the fluoroarene/heteroarene to initially form a fluorophosphorane, e.g., P(F)(^*n*^Bu)_3_(C_5_F_4_N), but that the silane then acts as a Lewis acid to abstract a fluoride ion from the phosphorane to form a phosphonium salt, e.g., [(C_5_F_4_N)P^*n*^Bu_3_][Ph_2_Si(F)H_2_]. We note that this reactivity is very similar to the addition of phosphines to the fluoroarene rings of B(C_6_F_5_)_3_, which is also followed by fluoride transfer to a Lewis acid to form a phosphonium borate salt.^7^ The hydrosilicate anion then transfers a hydride to the phosphonium ion to form another phosphorane that eliminates the hydrodefluorinated product and regenerates the phosphine (Scheme 4). This H/F exchange between P and Si is driven by differences in the fluoride ion and hydride ion affinities of the phosphorane and silane, which have been discussed and computed for related species.^74−76^ The reactivity of non-fluorinated pyridylphosphonium salts with nucleophiles to form substituted pyridines has also been reported and, like the processes observed here, is proposed to occur via phosphorane intermediates.^77−79^ Underpinning all of this reactivity is the facile redox cycling between P(III) and P(V) oxidation states that is remarkable to see in simple trialkylphosphines.

**Figure 1:**
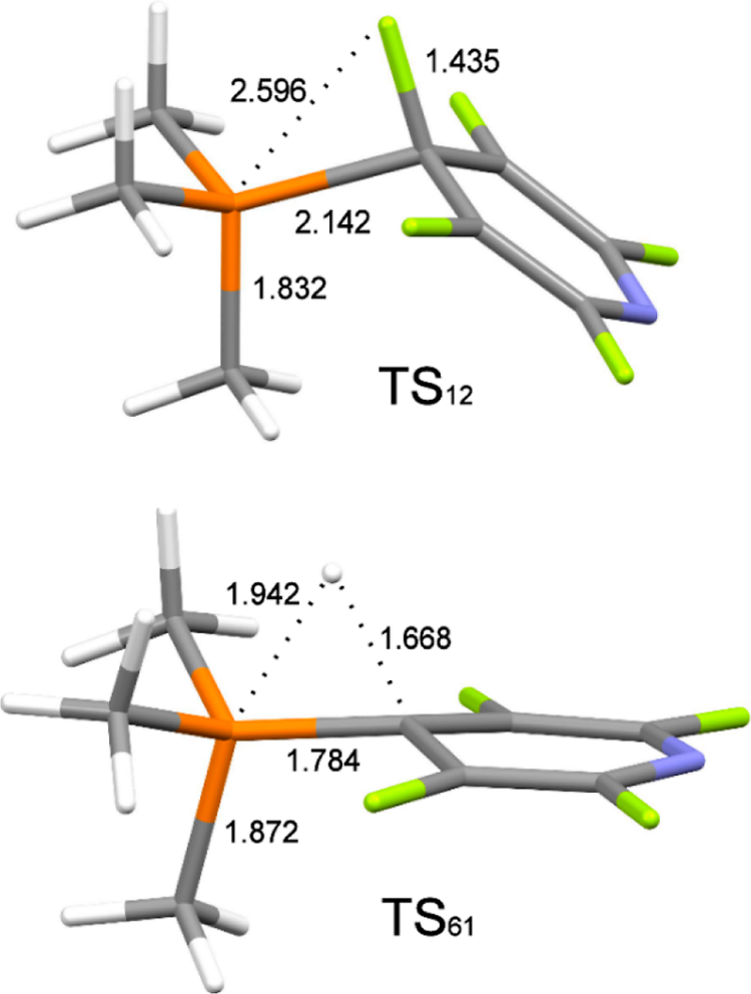
Transition states for the addition of PMe_3_ to **1** (**TS**_**12**_) and elimination of **2** from phosphorane **M6** (**TS**_**61**_). Hydrogen is shown in white, carbon in gray, phosphorus in orange, nitrogen in blue, and fluorine in green. Selected distances (in Å) are shown.

DFT calculations were performed to support the experimental mechanistic studies (see Supporting Information for details). These showed that initial reaction of the phosphine with pentafluoropyridine takes place through a Meisenheimer-like transition state (Scheme 4, **TS**_**12**_) to form a fluorophosphorane (**M2**) in a similar manner to that proposed by García and co-workers for reaction of **1** with PEt_3_.^64,65^ This is associated with a low barrier of 55 kJ mol^–1^. The structure of **TS**_**12**_ (Figure 1) shows the early nature of this transition state, where P–C bond formation and C–F bond elongation precede fluorine transfer to phosphorus to form the phosphorane. This step is effectively initiated by a nucleophilic addition of PR_3_ to the pentafluoropyridine and explains the preference for electron-poor arenes and heteroarenes in this reaction, where this will be promoted. No additional intermediates or transition states for fluorine transfer to phosphorus were identified. The addition of **1** to the phosphine leads to a formal oxidation state change from P(III) to P(V) and so can be characterized as an OA process, albeit one that is highly asynchronous in terms of C–F bond cleavage and reminiscent of concerted S_N_Ar mechanisms.^80^ The key structural parameters of **TS**_**12**_ are almost identical to those calculated by Dobrovetsky and co-workers for addition of **1** to a geometrically constrained σ^3^-P compound, suggesting a similar activation process, despite the very different structural frameworks involved.^21^

The fluorophosphorane that is initially formed (**M2**) can undergo isomerization to a lower energy isomer (**M3**) with fluorine *trans* to the tetrafluoropyridyl group, both in the apical positions. Fluoride transfer to Ph_2_SiH_2_ results in the formation of the observed phosphonium ion **[6]**^**+**^, in this case as the salt **M4**. Moving between neutral and ionic manifolds in this way will be strongly influenced by solvation effects. This leads to a small mismatch between the computed energies and the experimental observations, where **M4** is higher in energy than the phosphoranes, although phosphonium ions and not the phosphoranes are observed experimentally. This is likely due to limitations in using a dielectric continuum solvation model, which undersolvates the ions and raises their energies relative to neutral species. The neutral/ionic manifold switch may help to explain the solvent effect seen in this system, where moving from *o*-C_6_H_4_F_2_ (ε 13.4 = at 25 °C) to the more polar MeCN (ε = 35.9 at 25 °C) led to an increase in the catalytic rate.^81,82^ It is well-known that more polar solvents like MeCN promote the formation of ionic species from phosphoranes, and this would facilitate the formation of **M4**.

Hydride transfer from the silicate anion then leads to phosphorane **M5**, which can isomerize to form **M6**. This is very different from the mechanistic proposal of Dobrovetsky for HDF by geometrically constrained σ^3^-P systems,^21^ where it was suggested that PhSiH_3_ reacts directly with the fluorophosphorane through a transition state that involves concerted hydride transfer to P and fluoride transfer to Si. The experiments described above showed that for simple trialkylphosphines, phosphonium ions are intermediates, and these do not react directly with the neutral silanes. It may be that the positive charge on Dobrovetsky’s constrained σ^3^-P systems disfavors this pathway and leads to this divergence in mechanistic behavior.

The final step involves the RE of **2** from **M6** via **TS**_**61**_ (Figure 1). This transition state is more concerted than **TS**_**12**_, although still somewhat asynchronous, presumably because direct concerted RE from the axial and equatorial positions of a phosphorane is symmetry forbidden.^83^**TS**_**61**_ is again Meisenheimer-like, although less so than in the constrained σ^3^-P systems of Radosevich,^23^ where the C–H bond length of a related RE TS is 1.33 Å, and Dobrovetsky where it is 1.53 Å.^21^ It seems as though the substituents and geometric environment around phosphorus have a significant impact on the RE process. These final steps give rise to the energetic span for the reaction, which is defined by turnover-determining intermediate (TDI) **M5** and transition state (TDTS) **TS**_**61**_, and give an overall barrier for the catalytic reaction of 79 kJ mol^–1^. This low barrier is consistent with the observed fast reaction between **1** and Ph_2_SiH_2_ when a sterically relatively small phosphine like P^*n*^Bu_3_ is used as the catalyst (full conversion in 20 min at 20 °C in MeCN). The calculated energetic span for HDF of **1** by a constrained σ^3^-P system was significantly larger (140 kJ mol^–1^), which is consistent with the slower reactions observed in that study.^21^

## Conclusions

These data demonstrate that complex molecular architectures are not required to allow P(III) systems to act as catalysts for HDF or aminodefluorination of highly fluorinated arenes and heterocycles. In fact, simple trialkylphosphines were found to be fast and effective catalysts for these reactions for a range of substrates, but especially those that are highly electron poor. This is an important observation as some trialkylphosphines, especially the P^*n*^Bu_3_ used here, are cheap, readily available from commercial suppliers and simple to handle. This eliminates the barrier to entry that exists for some main-group catalyst systems, and so these reactions can be widely applied in a range of academic and industrial settings.

Mechanistic studies demonstrated that the catalytic HDF reactions described here are underpinned by metallomimetic behavior that is remarkable for such simple phosphines. Facile P(III)/P(V) redox cycling allows the phosphine to undergo OA of the substrates and RE of the products. Phosphonium ions, e.g., [(C_5_F_4_N)P^*n*^Bu_3_]^+^ ion, [**17**]^+^, were identified as key intermediates during catalysis. These are proposed to undergo hydride transfer from their hydrosilicate counterions, as part of a transition-metal-like transmetalation step in the catalytic cycle, prior to elimination of the products. This helps to explain the solvent and silane dependence of the observed reactions, where more polar solvents promote the formation of the phosphonium salts and specific substituents on the silane favor/disfavor formation of the silicate anions.

## Supporting Information Available

Experimental and computational methods, key spectroscopic data, and computational results, including energies and xyz coordinates (PDF)
