# Supplementary material for: Metallomimetic C–F Activation Catalysis by Simple Phosphines
Source: J Am Chem Soc. 2024 Jan 11;146(3):2005–14. doi: 10.1021/jacs.3c10614 (PMC10811696; doi:10.1021/jacs.3c10614)
Supplement: Supplementary file 1 — ja3c10614_si_001.pdf [file ja3c10614_si_001.pdf]

Metallomimetic C–F activation catalysis by simple phosphines

Sara Bonfante,[a,b] Christian Lorber,[b*] Jason M. Lynam,[a*] Antoine Simonneau[b*] and John M. Slattery[a*]

[a] Department of Chemistry, University of York, Heslington, York YO10 5DD, UK.

[b] LCC-CNRS, Université de Toulouse, CNRS, UPS, 205 route de Narbonne, BP 44099, F-31077 Toulouse cedex 4, France

Supporting Information

Contents

1. Experimental Details ... 3
1.1. General methods ... 3
1.2. Procedure for solvent optimisation ... 4
1.3. Procedure for catalyst optimisation ... 4
1.4. Procedure for silane optimisation ... 4
1.5. Procedure for optimisation of the catalyst loading ... 5
1.6. Procedure for optimisation of the silane loading ... 5
1.7. General procedure for the catalytic hydrodefluorination ... 6
1.8. Key Spectroscopic Data ... 7
1.9. Preparation of $Ph_2Si(Cl)(NEt_2)$ ... 15
1.10. Preparation of $Ph_2Si(Cl)$(pro) ... 17
1.11. Procedure for the catalytic aminodefluorination reaction ... 20
1.12. Procedure for the stoichiometric synthesis of 3 ... 31
2. Mechanistic Studies ... 32
2.1. Reactivity test of $PhSiH_3$ and pentafluoropyridine (1) ... 32
2.2. Reactivity test of Ph_2SiH_2 and P^nBu_3 ... 33
2.3. Reactivity test of Ph_2SiH_2 and pentafluoropyridine ... 34
2.4. Reactivity test of Ph_2SiH_2 and pentafluoropyridine in the presence of $[Me_4N]F$ ... 35
2.5. Characterisation of phosphonium salt [17]$[PhSi(H)_{4-n}F_n]$ ... 35
2.6. Characterisation and reactivity of phosphonium salt [17]Br ... 39
2.7. Observation of the phosphonium salt [18]$[Ph_2Si(H)_{3-n}F_n]$ ... 47
2.8. Characterisation and reactivity of the phosphonium salt [16]Br ... 49
3. Computational Chemistry ... 54
3.1. Tabulated energies for mechanism presented in scheme 4 ... 55
3.2. 3-component alternative mechanism for Si-H activation (c.f. Piers' mechanism) ... 57
3.3. xyz coordinates ... 58
4. References ... 72

1. Experimental Details

1.1. General methods

All reactions were performed in flame- or oven-dried glassware with rigorous exclusion of air and moisture, using a nitrogen-filled *MBraun* glove box (O_2 < 1 ppm, H_2O < 1 ppm) or regular Schlenk techniques.[1] Liquids were transferred using either plastic syringes, Teflon canulae with or without filtering tip, or Hamilton™ microsyringes. Unless otherwise indicated, the fluorinated aromatics and the silanes (Ph_3SiH, Ph_2SiH_2 and $PhSiH_3$) were purchased from Sigma Aldrich, Fluorochem or Apollo Scientific, dried over molecular sieves and degassed by freeze-pump-thaw cycles prior to use. CD_2Cl_2, CD_3CN, *o*-difluorobenzene (purchased from Merck), CH_3CN (pre-dried by passing through a Puresolv MD 7 solvent purification machine) were degassed by freeze-pump-thaw cycles, dried over CaH_2 and distilled. Et_2O was pre-dried by passing through a Puresolv MD 7 solvent purification machine. The phosphines, Ph_2SiCl_2, Et_2NH, Et_3N, $SOCl_2$ and *L*-proline were purchased from Sigma Aldrich and used without further purification. Tetramethylammonium fluoride was purchased from Sigma-Aldrich and dried at 100 °C for 48 h under hi-vac in a round bottom flask prior to its use. MeOH was purchased from Honeywell and dried over molecular sieves prior to use.

^{1}H, ^{31}P, ^{19}F and ^{13}C NMR spectra were recorded in CD_3CN, CD_2Cl_2 or C_6D_6 solution by using NMR tubes equipped with J. Young valves on a *Bruker* Avance III 500 (500 MHz for ^{1}H, 202 MHz for ^{31}P, 471 MHz for ^{19}F) or Avance NEO 600 (600 MHz for ^{1}H, 243 MHz for ^{31}P, 565 MHz for ^{19}F, 119 MHz for ^{29}Si) spectrometer at 25 °C. Chemical shifts are in parts per million (ppm) downfield from tetramethylsilane and are referenced to the residual solvent resonances as the internal standard (CHD_2CN: δ reported = 1.94 ppm; C_6HD_5: δ reported = 7.16 ppm; $CHDCl_2$: δ reported = 5.32 ppm for ^{1}H NMR). ^{13}C NMR spectra were calibrated according to the IUPAC recommendation using a unified chemical shift scale based on the proton resonance of tetramethylsilane as primary reference.[2,3] Data are reported as follows: chemical shift, multiplicity (br = broad, s = singlet, d = doublet, t = triplet, q = quartet, p = quintet, m = multiplet, mc = multiplet center), coupling constant (Hz), and integration.

MS spectra were measured using a Bruker Daltronics micrOTOF MS, Agilent series 1200LC with electrospray ionization (ESI and APCI) or on a Thermo LCQ using electrospray ionization, with <5 ppm error recorded for all HRMS samples. Mass spectral data are quoted as the m/z ratio along with the relative peak height in brackets (base peak = 100). Mass to charge ratios (m/z) are reported in Daltons. High-resolution mass spectra are reported with <5 ppm error.

1.2. Procedure for solvent optimisation

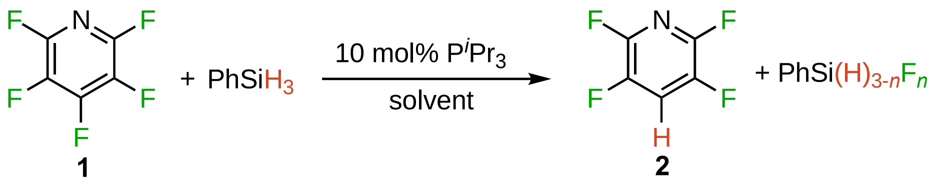

Pentafluoropyridine (44 µL, 0.40 mmol, 1.0 equivalent) and $PhSiH_3$ (50 µL, 0.40 mmol, 1.0 equivalent) were dissolved in 0.4 mL of the tested solvent and placed into a J-Young NMR tube. P^iPr_3 (7.6 µL, 0.040 mmol, 0.10 equivalent) was added and the tube was heated to monitor the progress of reaction (conditions specified in Table 1 of the manuscript). The reaction mixture was analyzed by quantitative ^{19}F NMR with trifluorotoluene (25 µL, 0.20 mmol, 0.50 equivalent) used as an internal standard, as described in section 1.7.

1.3. Procedure for catalyst optimisation

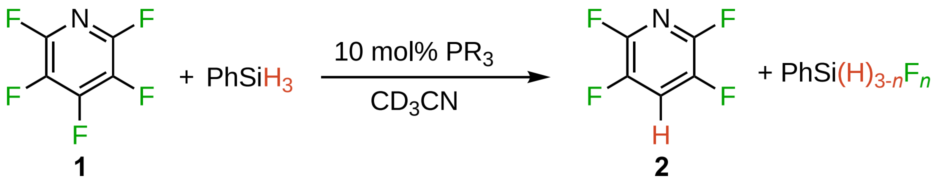

Pentafluoropyridine (44 µL, 0.40 mmol, 1.0 equivalent) and $PhSiH_3$ (50 µL, 0.40 mmol, 1.0 equivalent) were dissolved in 0.4 mL of CD_3CN and placed into a J-Young NMR tube. The tested phosphine (0.040 mmol, 0.10 equivalent) was added and the tube was heated to monitor the progress of reaction (conditions specified in Table 1 of the manuscript). The reaction mixture was analyzed by quantitative ^{19}F NMR with trifluorotoluene (25 µL, 0.20 mmol, 0.50 equivalent) used as an internal standard, as described in section 1.7.

1.4. Procedure for silane optimisation

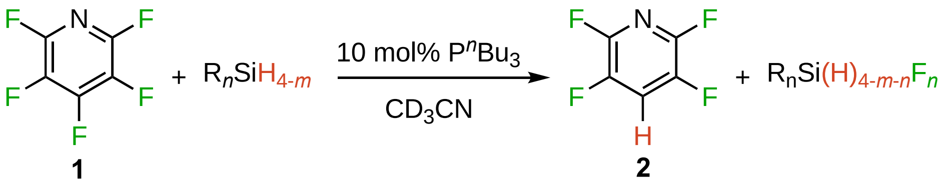

Pentafluoropyridine (44 µL, 0.40 mmol, 1.0 equivalent) and the tested silane (0.40 mmol, 1.0 equivalent) were dissolved in 0.4 mL of CD_3CN and placed into a J-Young NMR tube. P^nBu_3 (10 µL, 0.040 mmol, 0.10 equivalent) or P^iPr_3 (7.6 µL, 0.040 mmol, 0.10 equivalent) was added and the tube was heated to monitor the progress of reaction (conditions specified in Table 1 of the manuscript). The reaction mixture was analyzed by quantitative ^{19}F NMR with trifluorotoluene (25 µL, 0.20 mmol, 0.50 equivalent) used as an internal standard, as described in section 1.7.

1.5. Procedure for optimisation of the catalyst loading

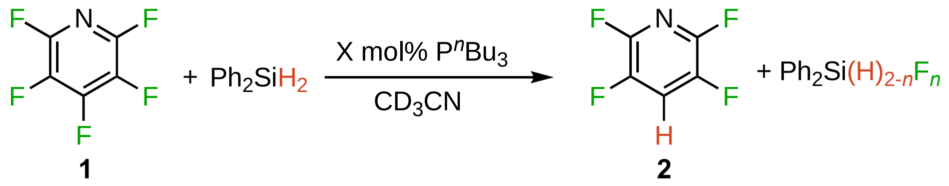

Pentafluoropyridine (44 µL, 0.40 mmol, 1.0 equivalent) and Ph_2SiH_2 (74 µL, 0.40 mmol, 1.0 equivalent) were dissolved in 0.4 mL of CD_3CN and placed into a J-Young NMR tube. Different amounts of P^nBu_3 were added (10 mol%, 5 mol% or 1 mol%) and the progress of reaction was monitored (conditions specified in Table 1 of the manuscript). The reaction mixture was analyzed by quantitative ^{19}F NMR with trifluorotoluene (25 µL, 0.20 mmol, 0.50 equivalent) used as an internal standard, as described in section 1.7.

1.6. Procedure for optimisation of the silane loading

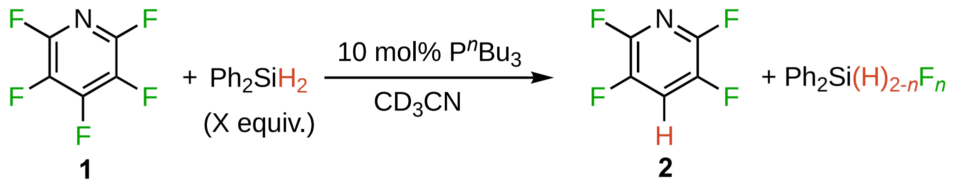

Pentafluoropyridine (44 µL, 0.40 mmol, 1.0 equivalent) and different amounts of Ph_2SiH_2 (1.0 equivalent and 0.55 equivalents) were dissolved in 0.4 mL of CD_3CN and placed into a J-Young NMR tube. P^nBu_3 (10 µL, 0.040 mmol, 0.1 equivalent) was added and the progress of reaction was monitored (conditions specified in Table 1 of the manuscript). The reaction mixture was analyzed by quantitative ^{19}F NMR with trifluorotoluene (25 µL, 0.20 mmol, 0.50 equivalent) used as an internal standard, as described in section 1.7.

1.7. General procedure for the catalytic hydrodefluorination

$$Ar^F\text{-}F + Ph_2SiH_2 \xrightarrow[CD_3CN]{10\ mol\%\ P^nBu_3} Ar^F\text{-}H + Ph_2Si(H)_{2\text{-}n}F_n$$

The Ar–F (0.40 mmol, 1.0 equivalent) and Ph_2SiH_2 (74 µL, 0.40 mmol, 1.0 equivalent) were dissolved in 0.4 mL of CD_3CN and placed into a J-Young NMR tube. P^nBu_3 (10 µL, 0.10 equivalent) was added and the progress of the reaction was monitored by ^{19}F NMR spectroscopy. If the hydrodefluorination product was volatile, rather than isolating it the reaction mixture was analyzed by quantitative ^{19}F NMR with trifluorotoluene used as an internal standard. Inversion recovery experiments were performed on **1** to determine appropriate parameters for these experiments. The *meta*-F signal at –162.70 ppm was on resonance throughout the experiment. The D1 was set at 10 seconds and a total of seven values ranging from 1 millisecond to 30 seconds were used for the inversion recovery delay (T1). It was observed that the *meta*-F exhibited the longest T1 value, measuring 7.8 seconds. Thus, the D1 parameter was set to 39 seconds for all the ^{19}F NMR experiments involving fluorinated pyridines. The product conversion was calculated from the ratio of the integrals of the trifluorotoluene and Ar–F signals before the reaction (blank sample) and the ratio of the trifluorotoluene and Ar–H signals at the end of the reaction. In the case of decafluorobiphenyl hydrodefluorination, after completion of the reaction the solution was evaporated and the crude product fully dried. The residue was dissolved in *n*-hexane and the resulting mixture was purified by chromatography column. The hexane was then evaporated and the residue was thoroughly dried to give an isolated yield of product (**11**).

Spectroscopic data for the products:

2,3,5,6-tetrafluoropyridine (2):[4] Yield of 93%. ^1H NMR (600 MHz, CD_3CN): 7.88 (tt, $^3J_{HF}$ = 7.9 Hz, $^4J_{HF}$ = 7.1 Hz, 1H). ^{19}F NMR (565 MHz, CD_3CN): δ –93.63 (mc, 2F, *ortho*-F), –141.55 (mc, 2F, *meta*-F).

3,5,6-trifluoropyridine (4):[5] Yield of 13%. ^1H NMR peaks have not been identified due to the low conversion and the overlapping of Ph_2SiH_2, 2,3,5,6-tetrafluoropyridine and trifluorotoluene signals in the ^1H NMR spectrum. ^{19}F NMR (565 MHz, CD_3CN): δ –92.34 (t, $^3J_{FF}$ = 27.9 Hz, 1F, 6-C*F*), –128.98 (ddd, $^5J_{FF}$ = 29.1 Hz, $^3J_{FH}$ = 7.6 Hz, $^3J_{FF}$ = 3.6 Hz, 1F, 5-CF), –136.47 (ddd, $^3J_{FH}$ = 26.3 Hz, $^3J_{FH}$ = 9.2 Hz, $^3J_{FF}$ = 3.7 Hz, 1F, 3-C*F*).

2,6-difuoropyridine (5):[6] Yield of 79%. ^1H NMR (600 MHz, CD_3CN): δ 7.99 (m, 1H, *para*-H), 6.93 (d, *J* = 8.0 Hz, 2H, *meta*-H). ^{19}F NMR (565 MHz, CD_3CN): δ –70.33 (bs, 2F).

2,3,5,6-tetrafluorobenzonitrile (6):[4] Yield of 86%. ^1H NMR (600 MHz, CD_3CN): δ 7.67 (m, 1H). ^{19}F NMR (471 MHz, CD_3CN): –134.81 (mc, 2F), –137.58 (mc, 2F).

1,2,4,5-tetrafluoro-3-(trifluoromethyl)benzene (7):[4] Yield of 89%. ^{1}H NMR (600 MHz, CD_3CN): δ 7.63 (m, 1H). ^{19}F NMR (565 MHz, CD_3CN): δ –57.35 (t, $^{4}J_{FF}$ = 22.3 Hz, 3F, CF_3), –138.41 (mc, 2F), –142.65 (mc, 2F).

1,2,4,5-tetrafluorobenzene (8):[7] Yield of 84% when Ar–F is C_6F_6, 93% when Ar–F is C_6F_5H. ^{1}H NMR (500 MHz, CD_3CN): δ 7.26 (p, *J* = 8.9 Hz, 2H). ^{19}F NMR (471 MHz, CD_3CN): δ –141.03 (t, *J* = 8.9 Hz, 4F).

2,2',3,3',5,5',6,6'-octafluoro-1,1'-biphenyl (11):[4] Isolated yield of 86%. ^{1}H NMR (600 MHz, CD_2Cl_2): δ 7.37 – 7.27 (m, 2H). ^{19}F NMR (565 MHz, CD_3CN): δ –138.65 – –138.78 (m, 2F), –139.12 – –139.25 (m, 2F).

[16]Br: ^{1}H NMR (500 MHz, CD_3CN): δ 2.93 (td, *J* = 12.9, 8.6 Hz, 6H), 1.65 (dq, *J* = 16.6, 8.6 Hz, 6H), 1.59 – 1.49 (m, 6H), 0.95 (t, $^{3}J_{HH}$ = 7.4 Hz, 9H). {^{1}H}^{31}P NMR (202 MHz, CD_3CN): δ 36.79 – 36.50 (m, 1P). ^{19}F NMR (471 MHz, CD_3CN): δ –128.37 (mc, 2F), –143.48 (mc, 1F), –158.54 (mc, 2F).

1.8. Key Spectroscopic Data

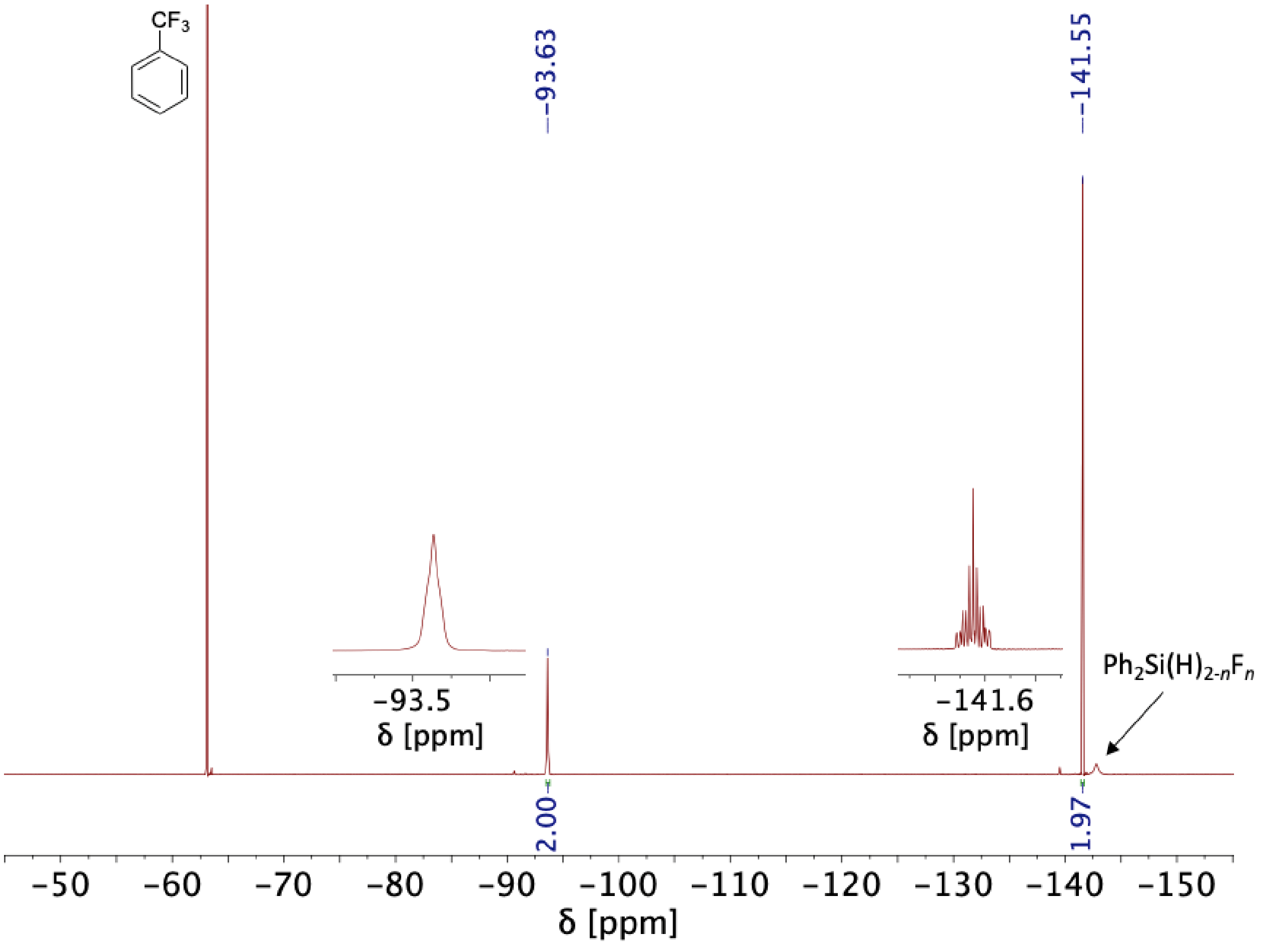

Figure S1: ^{19}F NMR spectrum (565 MHz, CD_3CN) of 2,3,5,6-tetrafluoropyridine (**2**) obtained after 20 minutes at 20 °C, using the general procedure in section 1.7.

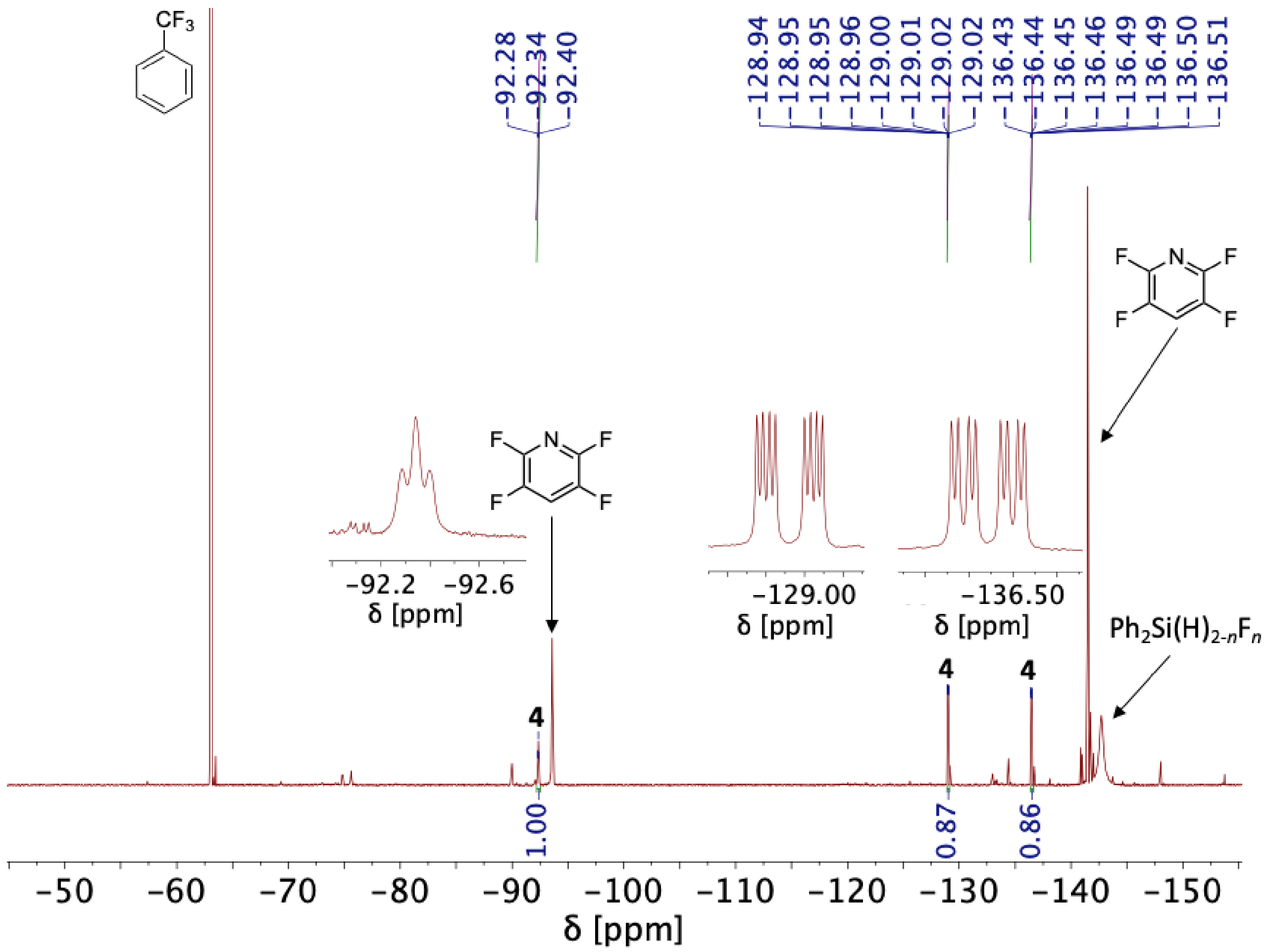

Figure S2: ^{19}F NMR spectrum (565 MHz, CD_3CN) of 3,5,6-trifluoropyridine (**4**) obtained after 5 days at 70 °C, using the general procedure in section 1.7.

.

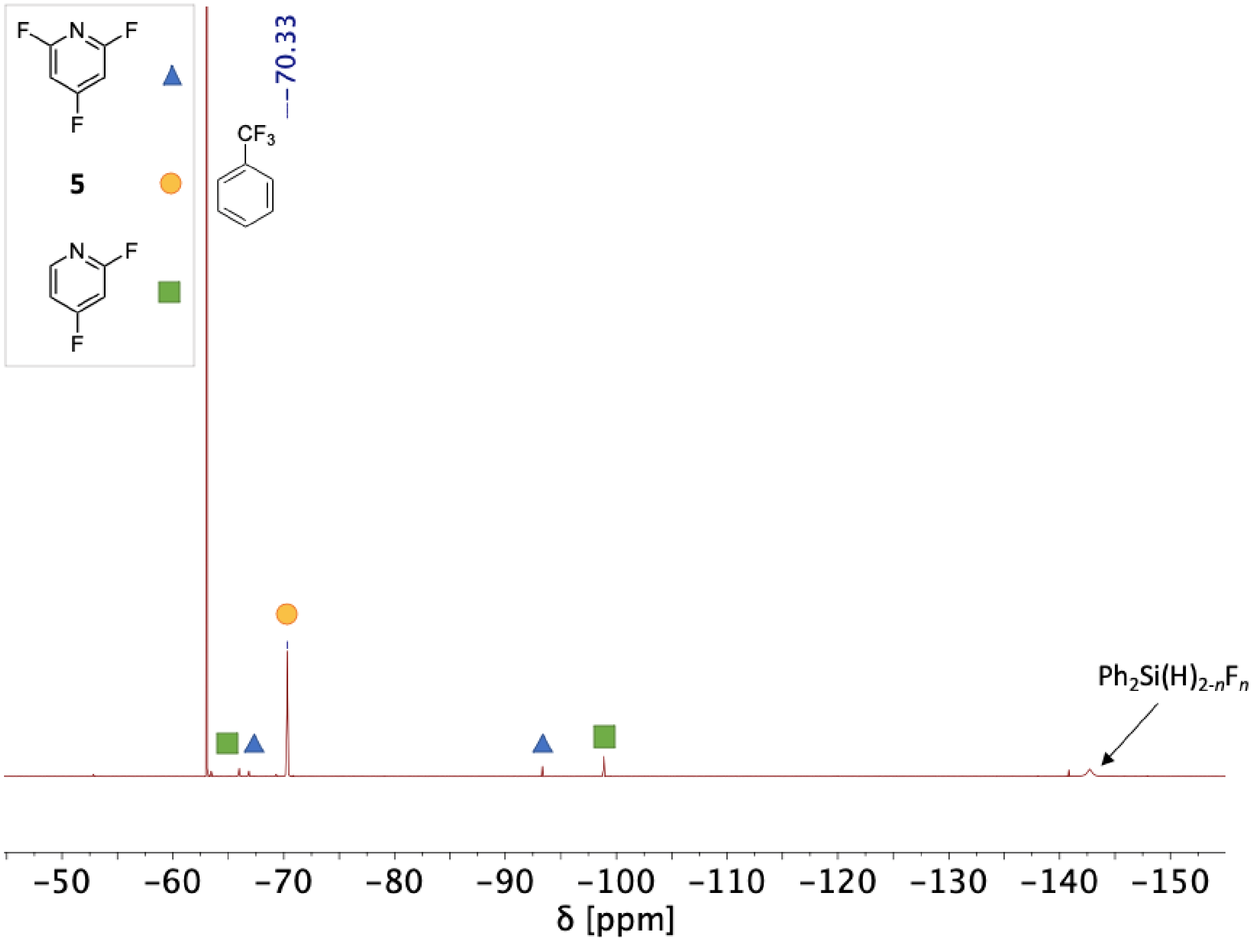

Figure S3: ^{19}F NMR spectrum (565 MHz, CD_3CN) of 2,6-difluoropyridine (**5**) obtained after 6 days at 60 °C, using the general procedure in section 1.7.

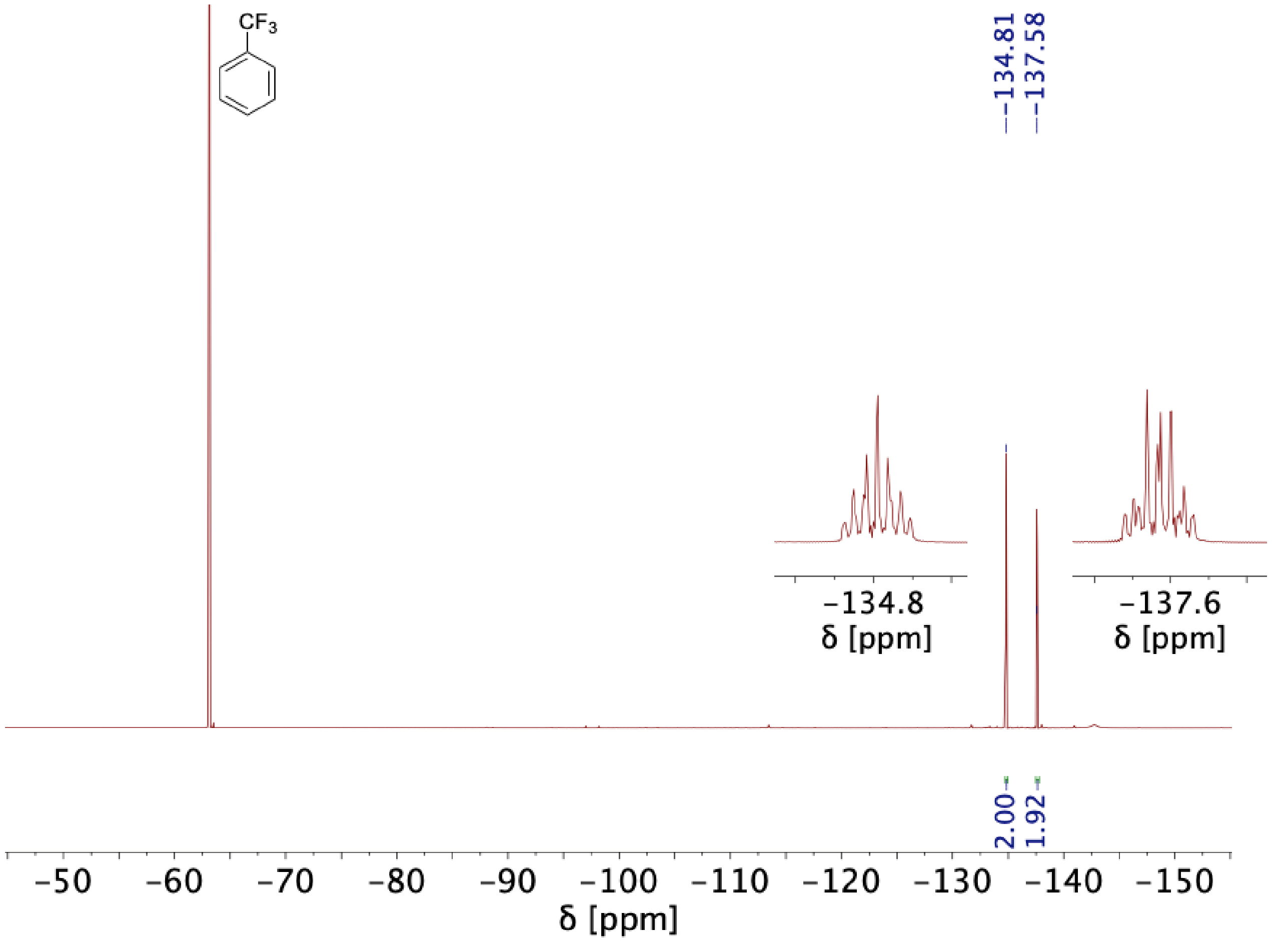

Figure S4: ^{19}F NMR spectrum (565 MHz, CD_3CN) of 2,3,5,6-tetrafluorobenzonitrile (**6**) obtained after 10 minutes at 20 °C, using the general procedure in section 1.7.

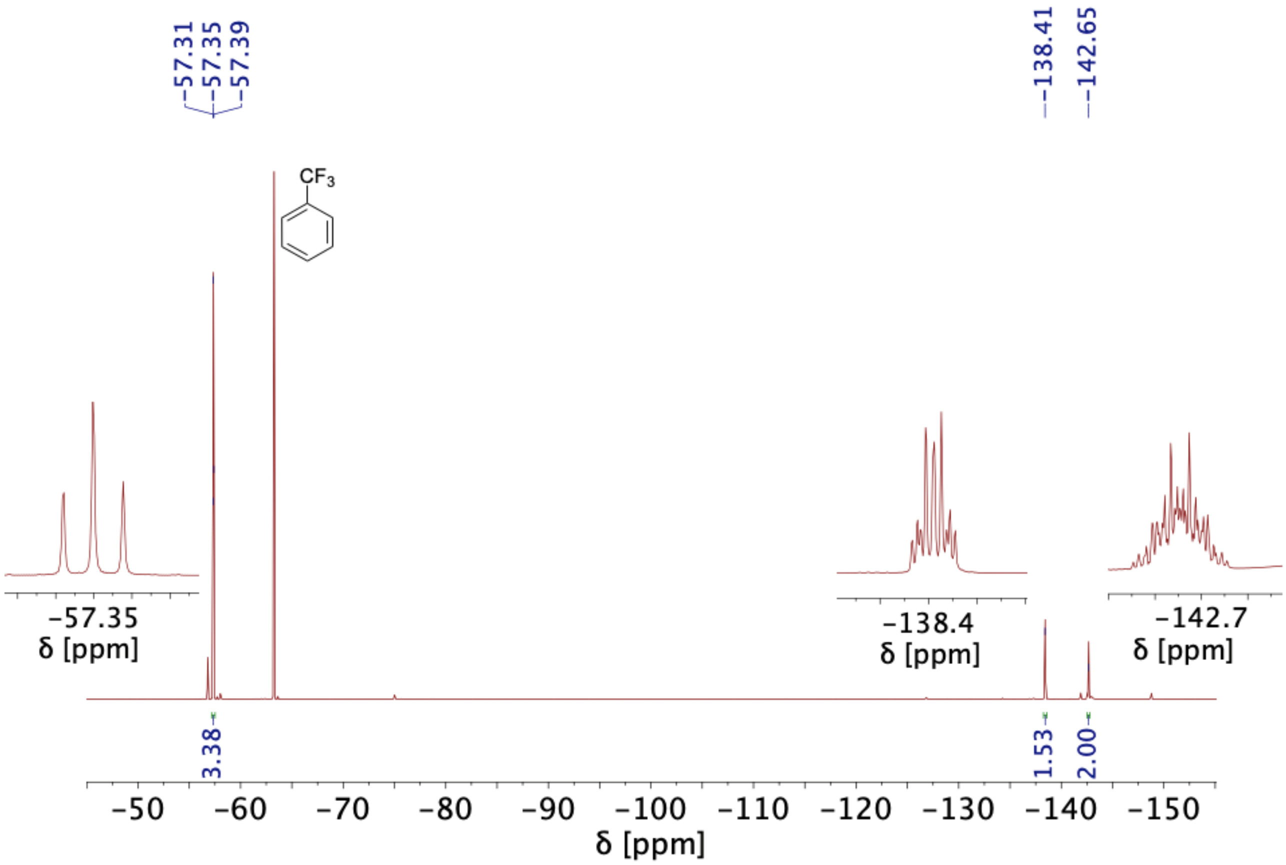

Figure S5: ^{19}F NMR spectrum (565 MHz, CD_3CN) of 1,2,4,5-tetrafluoro-3-(trifluoromethyl)benzene (**7**) obtained after 3 hours at 20 °C, using the general procedure in section 1.7.

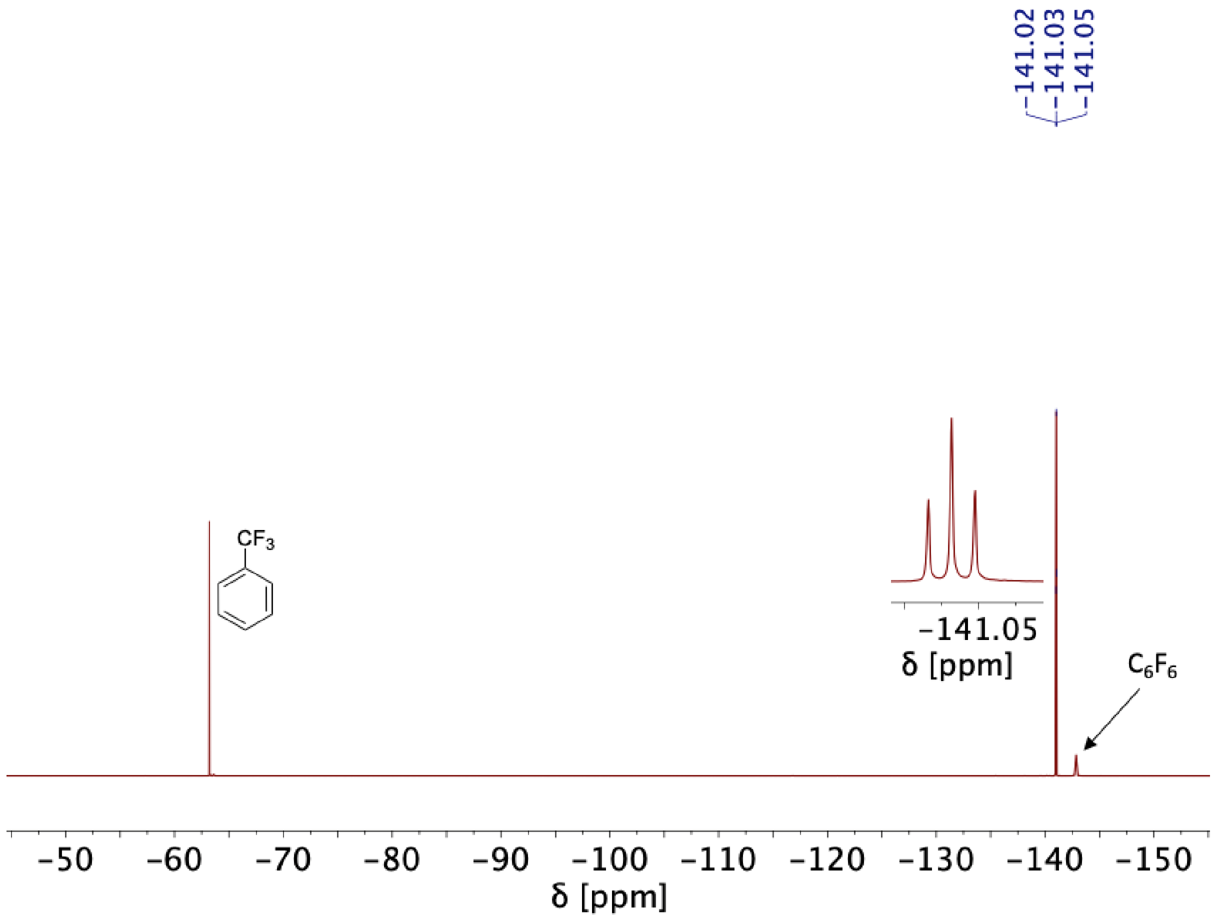

Figure S6: ^{19}F NMR spectrum (565 MHz, CD_3CN) of 1,2,4,5-tetrafluorobenzene (**8a**) obtained after 9 days at 60 °C, using the general procedure in section 1.7.

.

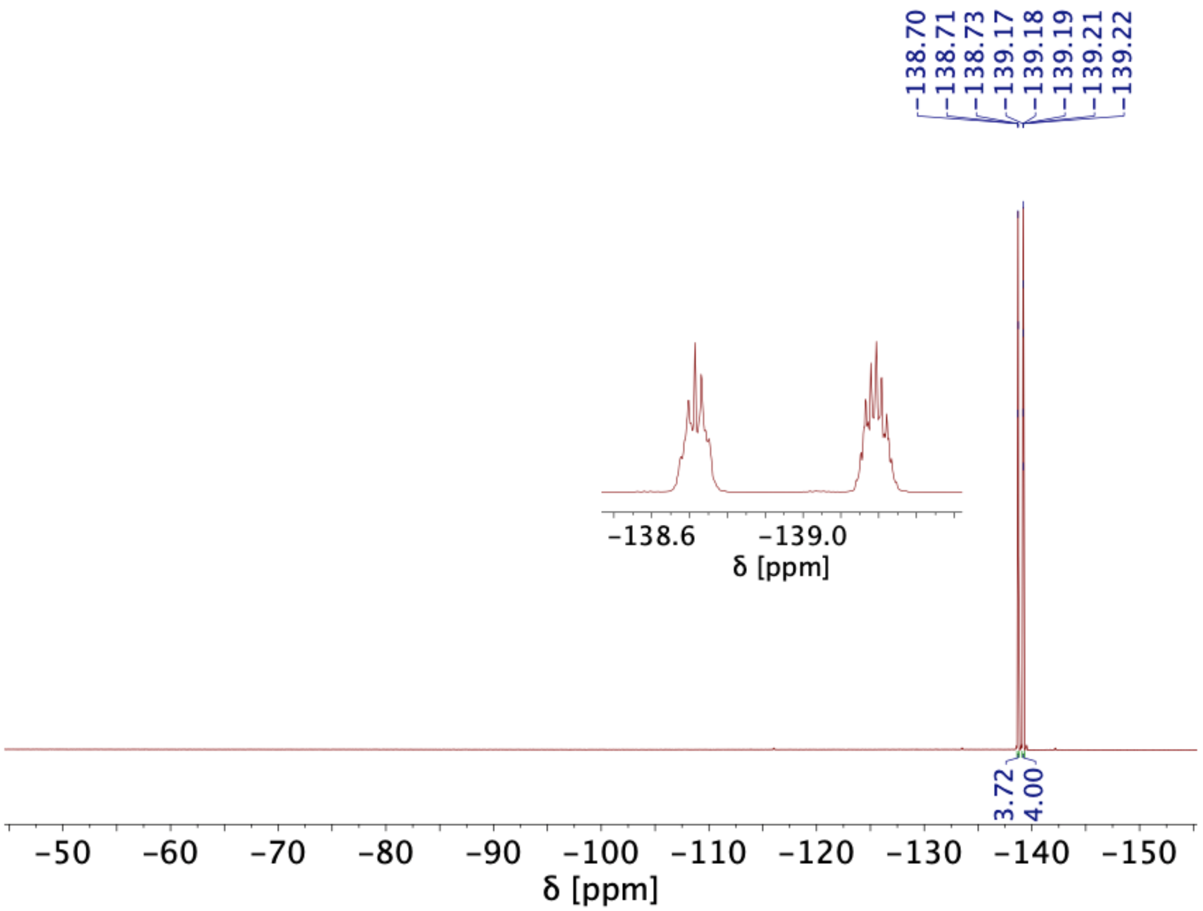

Figure S8: ^{19}F NMR spectrum (565 MHz, CD_3CN) of 2,2',3,3',5,5',6,6'-octafluoro-1,1'-biphenyl (**11**) isolated and purified according to the general procedure in section 1.7.

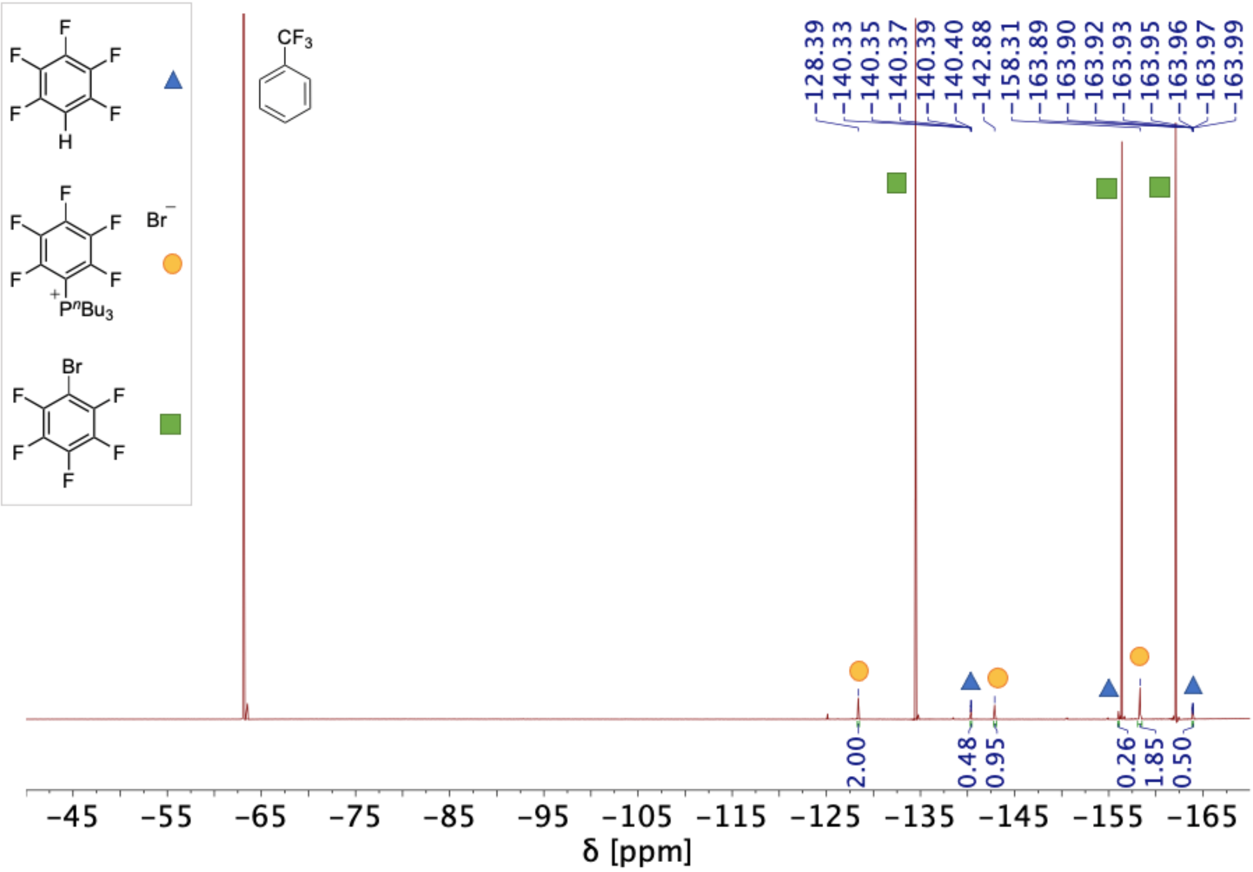

Figure S7: ^{19}F NMR spectrum (565 MHz, CD_3CN) of the mixture resulting from the catalytic HDF attempt of bromopentafluorobenzene and the assigned phosphonium salt **[16]Br** obtained after 20 minutes 20 °C, using the general procedure in section 1.7.

1.9. Preparation of $Ph_2Si(Cl)(NEt_2)$

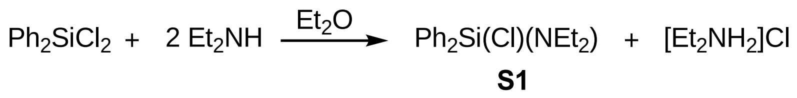

This product was prepared according to a literature method.[8] In a Schlenk tube were placed 1.2 mL (5.9 mmol, 1.0 equiv.) of diphenyldichlorosilane and 13 ml of ether. The solution was cooled to –78°C in a dry-ice/acetone bath and treated with a solution of 2.4 mL (24 mmol, 4.0 equiv.) of diethylamine in 4.4 mL of ether over a 30 min period, causing formation of a voluminous precipitate of diethylamine hydrochloride. The mixture was filtered under a positive pressure of nitrogen with a filter-stick, the solid was washed with diethylether (3 x 7 mL), the ether layers were combined and the product dried under vacuum. A yellow oil was obtained in 78% yield. N.B. The product may fume in contact with air.

^1H NMR (C_6D_6, 600 MHz): δ 7.88 – 7.84 (m, 4H), 7.17 – 7.13 (m, 6H), 2.86 (q, $^3J_{HH}$ = 7 Hz, 4H), 0.92 (t, $^3J_{HH}$ = 7 Hz, 6H). ^{29}Si NMR (C_6D_6, 119 MHz): –6.56 (s, 1Si). MS-APCI: $[C_{16}H_{21}ClNSi]^+$ 290.113336 *m/z* (2.4 ppm deviation from theoretical mass).

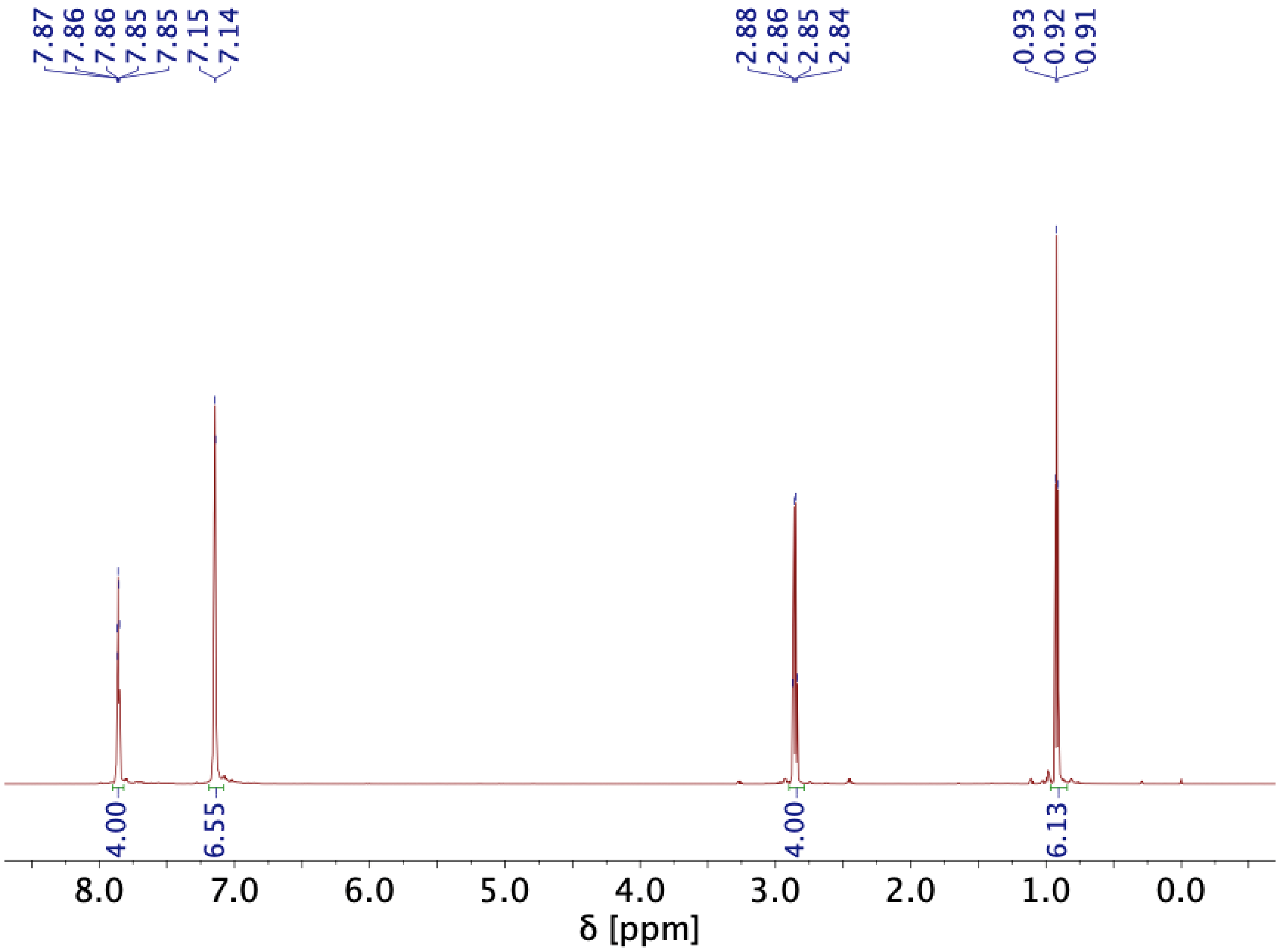

Figure S9: ^1H NMR spectrum (C_6D_6, 600 MHz) of $Ph_2Si(Cl)(NEt_2)$.

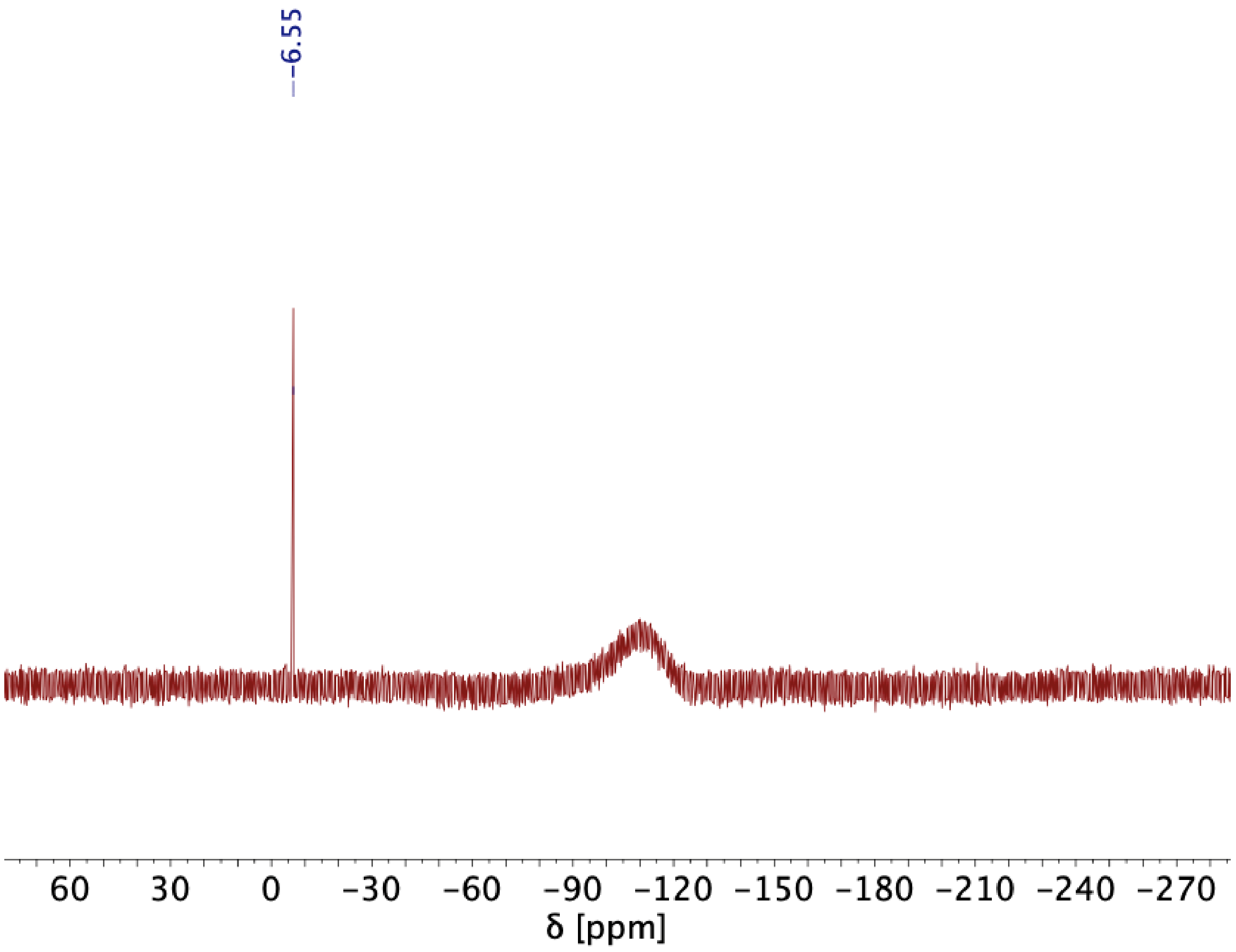

Figure S10: ^{29}Si NMR spectrum (C_6D_6, 119 MHz) of $Ph_2Si(Cl)(NEt_2)$. The broad signal corresponds to Si in the glass NMR tube.

1.10. Preparation of $Ph_2Si(Cl)(pro)$

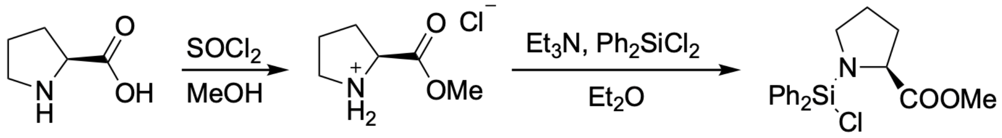

S2 **S3**

L-Proline methyl ester hydrochloride (**S2**) was prepared according to a literature method.[9] Thionyl chloride (0.72 mL, 10 mmol, 1.2 equivalent) was added dropwise to anhydrous methanol (42 mL) at 0 °C. The solution was stirred at 0 °C for 30 min and *L*-Proline (0.95 g, 8.3 mmol, 1 equivalent) was added. The reaction mixture was refluxed for 5 h and TLC (CH_3Cl/MeOH, 9/1) indicated complete disappearance of *L*-Proline. The reaction mixture was evaporated under reduced pressure and a yellow oil was obtained.

Silane **S3** was then prepared following a modified literature procedure.[8] The obtained hydrochloride salt, **S2**, (1.6 g, 10 mmol, 1.2 equivalent) was dissolved in 11 mL of ether and Et_3N (2.8 mL, 20 mmol, 2.4 equivalents) was added in the solution to obtain the *L*-Proline methyl ester. 1.0 mL (5.0 mmol, 0.60 equivalent) of diphenyldichlorosilane and 3.8 mL of ether were placed in another Schlenck tube. The solution was cooled to –78°C in a dry-ice/acetone bath and treated with the solution of *L*-Proline methyl ester over a 30 min period, causing formation of a voluminous precipitate. Filtration under a positive pressure of nitrogen with a filter-stick, washing the solid with ether, combination of the ether layers and drying the product under vacuum followed. The desired product was extracted with pentane (2 × 10 mL). A yellow oil was obtained in 43% yield.

^{1}H NMR (500 MHz, DCM-d_2): δ 7.91 – 7.85 (m, 2H), 7.56 – 7.42 (m, 8H), 3.94 (dd, $^3J_{HH}$ = 8.6, $^3J_{HH}$ = 3.3 Hz, 1H), 3.47 (s, 3H), 2.23 – 2.09 (m, 2H), 2.02 – 1.88 (m, 4H). ^{29}Si NMR (C_6D_6, 119 MHz): –8.90 (s, 1Si). ^{13}C NMR (151 MHz, DCM-d_2): δ 175.62, 135.02, 130.71, 128.08, 60.63, 51.36, 47.65, 31.67, 25.93. MS-APCI: $[C_{18}H_{20}NO_2Si]^+$ 310.126372 *m/z* (1.9 ppm deviation from theoretical mass); $[C_{18}H_{21}ClO_2Si]^+$ 346.102908 *m/z* (1.3 ppm deviation from theoretical mass). $[\alpha]_D^{20}$ –25° (*c* 0.5, MeOH).

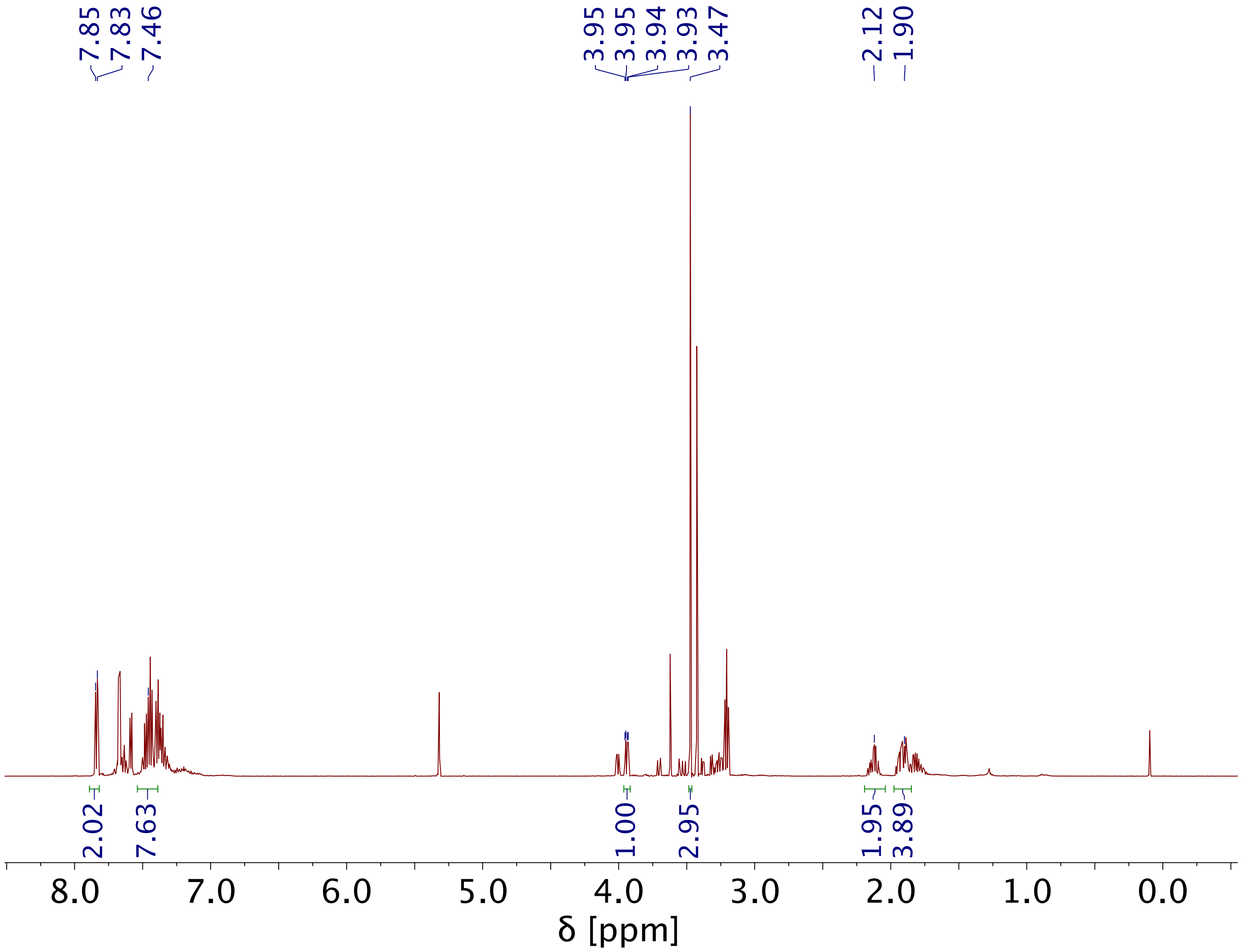

Figure S11: ^{1}H NMR spectrum (CD_2Cl_2, 600 MHz) of $Ph_2Si(Cl)(pro)$.

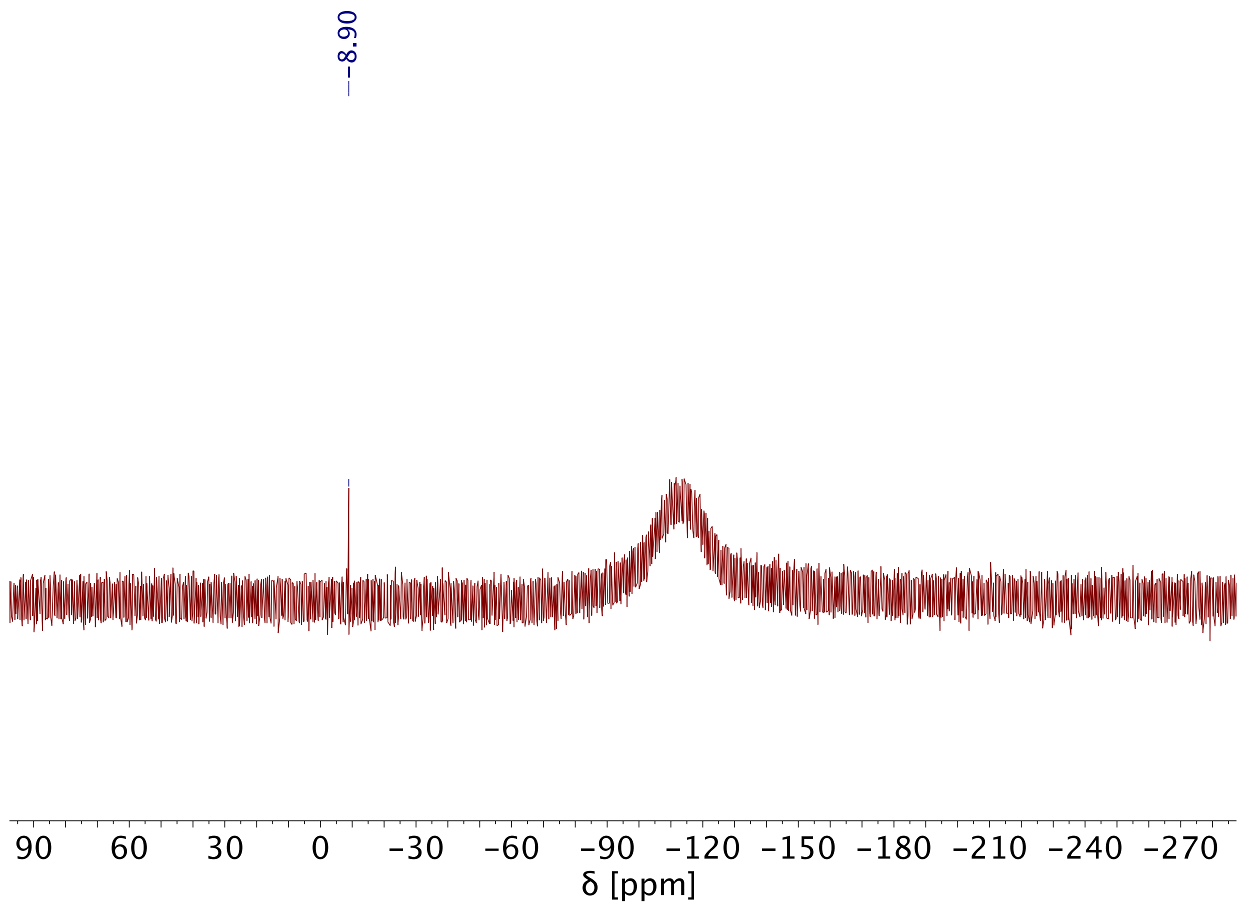

Figure S12: ^{29}Si NMR spectrum (CD_2Cl_2, 119 MHz) of $Ph_2Si(Cl)(pro)$. The broad signal corresponds to Si in the glass NMR tube.

1.11. Procedure for the catalytic aminodefluorination reaction

$$Ar^F\text{-}F + Ph_2Si(Cl)(NR_2) \xrightarrow[CD_3CN]{10\text{ mol\% }P^nBu_3} Ar^F\text{-}NR_2 + Ph_2Si(Cl)F + Ph_2SiF_2$$

S1, $NR_2 = NEt_2$
S3, NR_2 = L-proline ester

The Ar–F (0.40 mmol, 1.0 equivalent) and $Ph_2Si(Cl)(NR_2)$ (0.44 mmol, 1.1 equivalent) were dissolved in 0.4 mL of CD_3CN and placed into a J-Young NMR tube. P^nBu_3 (10 µL, 0.10 equivalent) was added and the system was heated at 60 °C for the synthesis of **12** and **13**, 80 °C for **14** and **15**. The reaction mixture was analyzed by quantitative ^{19}F NMR with trifluorotoluene (25 µL, 0.20 mmol, 0.50 equivalent) used as an internal standard, as described in section 1.7.

N,N-diethyl-2,3,5,6-tetrafluoropyridin-4-amine (12):[10] Yield of 75%. ^1H NMR (600 MHz, CD_3CN): δ 3.43 (qt, $^3J_{HH}$ = 7.1 Hz, $^5J_{HF}$ = 1.6 Hz, 4H), 1.22 (t, $^3J_{HH}$ = 7.1 Hz, 6H). ^{19}F NMR (565 MHz, CD_3CN): δ –96.66 (mc, 2F), –157.05 (mc, 2F). GC-MS(EI) $[C_9H_{10}N_2F_4]^{+\circ}$ 222.07810 *m/z* (2.85 ppm deviation from theoretical mass); $[C_8H_7F_4N_2]^{+\circ}$ 207.05472 *m/z*; $[C_7H_5F_4N_2]^{+\circ}$ 193.03912 *m/z*.

Methyl 2,3,5,6-tetrafluoropyrid-4-ylpyrrolidine-2-carboxylate (13): Yield of 88%. ^1H NMR (500 MHz, CD_3CN): δ 4.83 (dq, *J* = 8.2, 3.5 Hz, 1H), 3.92 (dtt, *J* = 10.1, 6.7, 3.3 Hz, 1H), 3.88 – 3.84 (m, 1H), 3.73 (s, 3H), 2.32 (dq, *J* = 12.9, 7.6 Hz, 1H), 2.06 (dq, *J* = 12.2, 6.1 Hz, 1H), 1.95 (p, *J* = 6.8 Hz, 2H). ^{19}F NMR (565 MHz, CD_3CN): δ –97.03 (mc, 2F), –160.09 (mc, 2F). ^{13}C (151 MHz, CD_3CN): δ 62.88 (t, *J* = 5.4 Hz, *C*H), 52.25 (t, *J* = 6.6 Hz, CH_2), 52.06 (s, CH_3), 30.28 (bs, CH_2), 23.58 (t, *J* = 2.0 Hz, CH_2). GC-MS(EI) $[C_{11}H_{10}N_2O_2F_4]^{+\circ}$ 278.06880 *m/z* (5.41 ppm deviation from theoretical mass); $[C_9H_7N_2F_4]^{+\circ}$ 219.05493 *m/z*; ; $[C_6HN_2F_4]^{+\circ}$ 177.00778 *m/z*. $[\alpha]_D^{20}$ –65 (*c* 2.5, CH_2Cl_2).

4-(diethylamino)-2,3,5,6-tetrafluorobenzonitrile (14):[10] Yield of 82%. ^1H NMR (600 MHz, CD_3CN): δ 3.37 (qt, $^3J_{HH}$ = 7.1 Hz, $^5J_{HF}$ = 1.4 Hz, 4H), 1.15 (t, $^3J_{HH}$ = 7.1 Hz, 6H). ^{19}F NMR (565 MHz, CD_3CN): δ –137.99 (mc, 2F), –151.34 (mc, 2F).

N,N-diethyl-2,3,5,6-tetrafluoro-4-(trifluoromethyl)aniline (15):[10] Yield of 50%. ^1H NMR (600 MHz, CD_3CN): δ 3.33 (qt, $^3J_{HH}$ = 7.1 Hz, $^5J_{HF}$ = 1.1 Hz, 4H), 1.13 (t, $^3J_{HH}$ = 7.1 Hz, 6H). ^{19}F NMR (565 MHz, CD_3CN): δ –56.05 (t, *J* = 21.1 Hz), –145.49 (mc, 2F), –151.27 (mc, 2F).

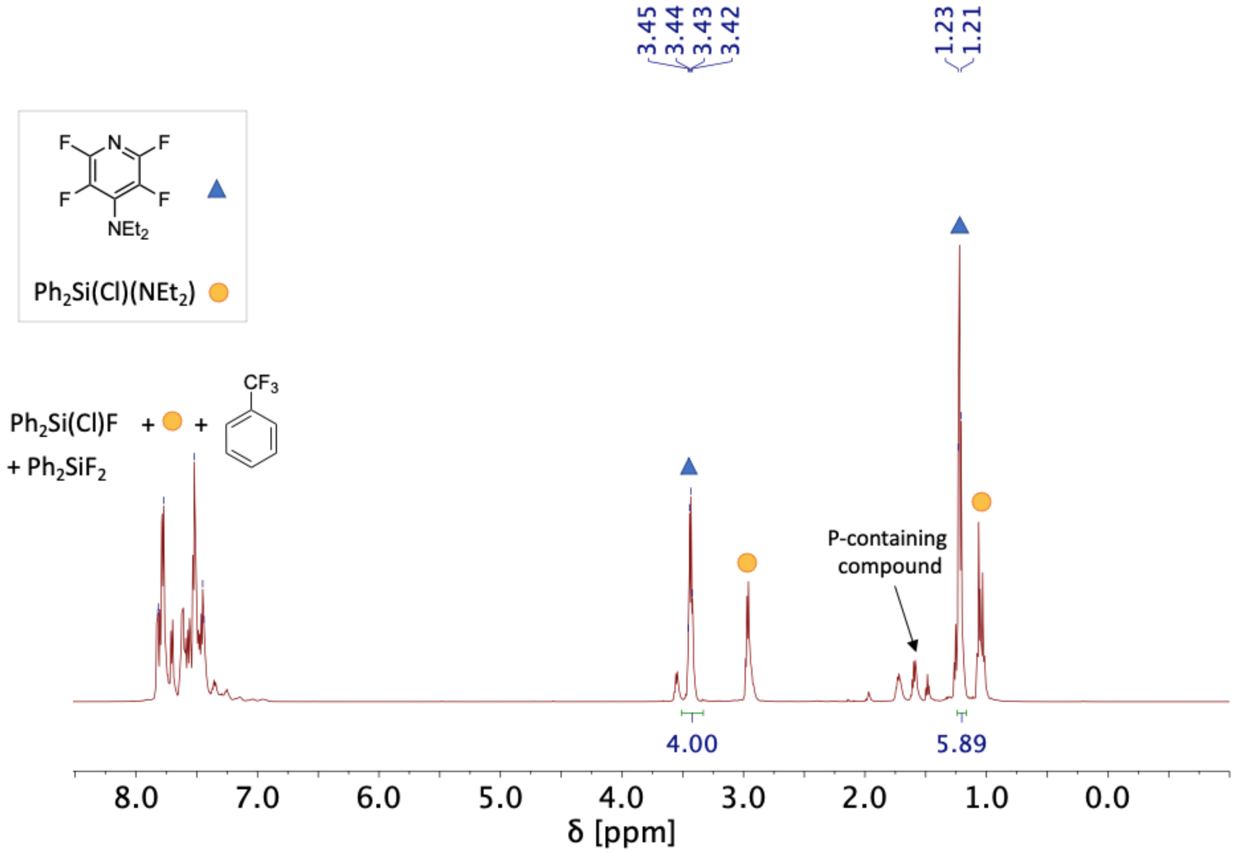

Figure S13: ^{1}H NMR spectrum (600 MHz, CD_3CN) of N,N-diethyl-2,3,5,6-tetrafluoropyridin-4-amine (**12**) obtained after 18 hours at 60 °C, using the general procedure in section 1.11.

.

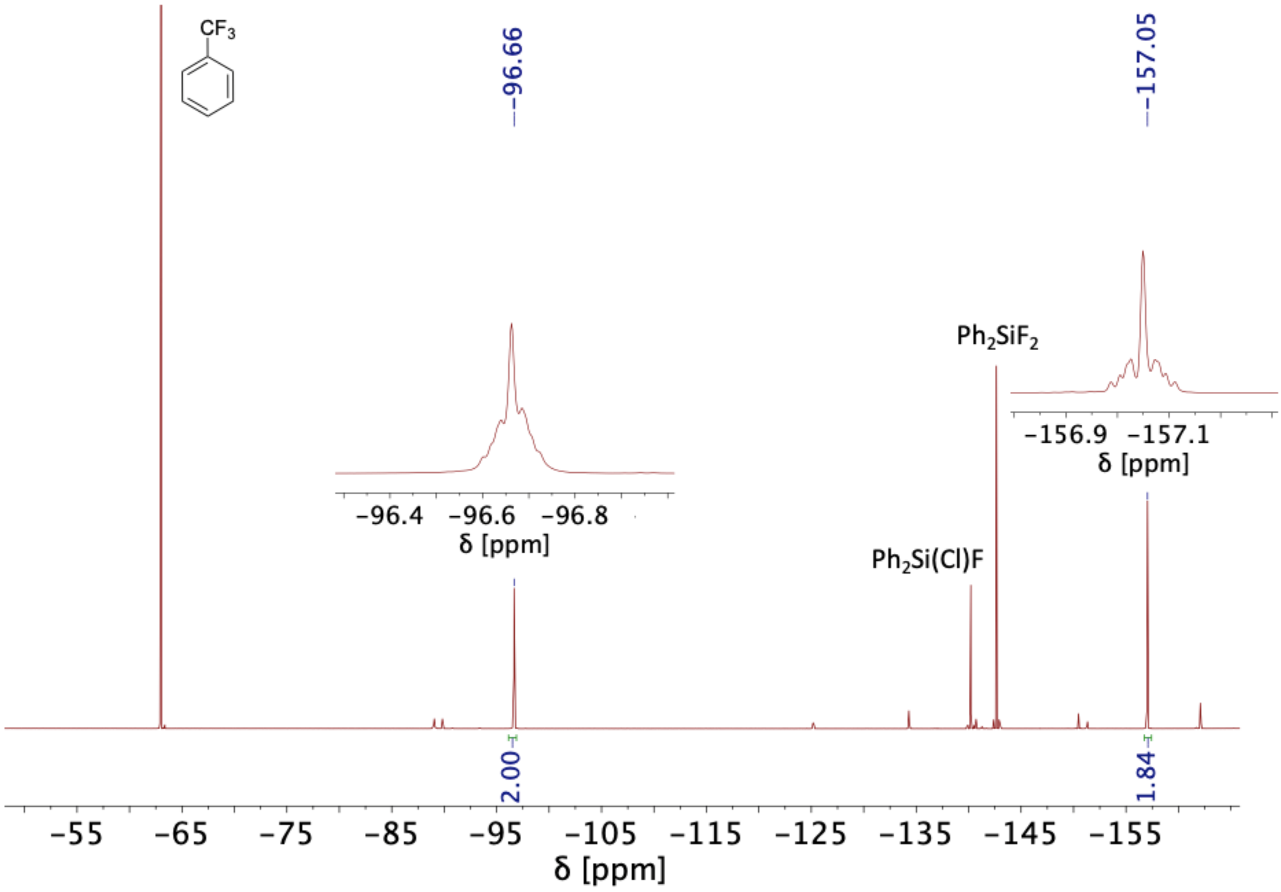

Figure S14: ^{19}F NMR spectrum (565 MHz, CD_3CN) of N,N-diethyl-2,3,5,6-tetrafluoropyridin-4-amine (**12**) obtained after 18 hours at 60 °C, using the general procedure in section 1.11.

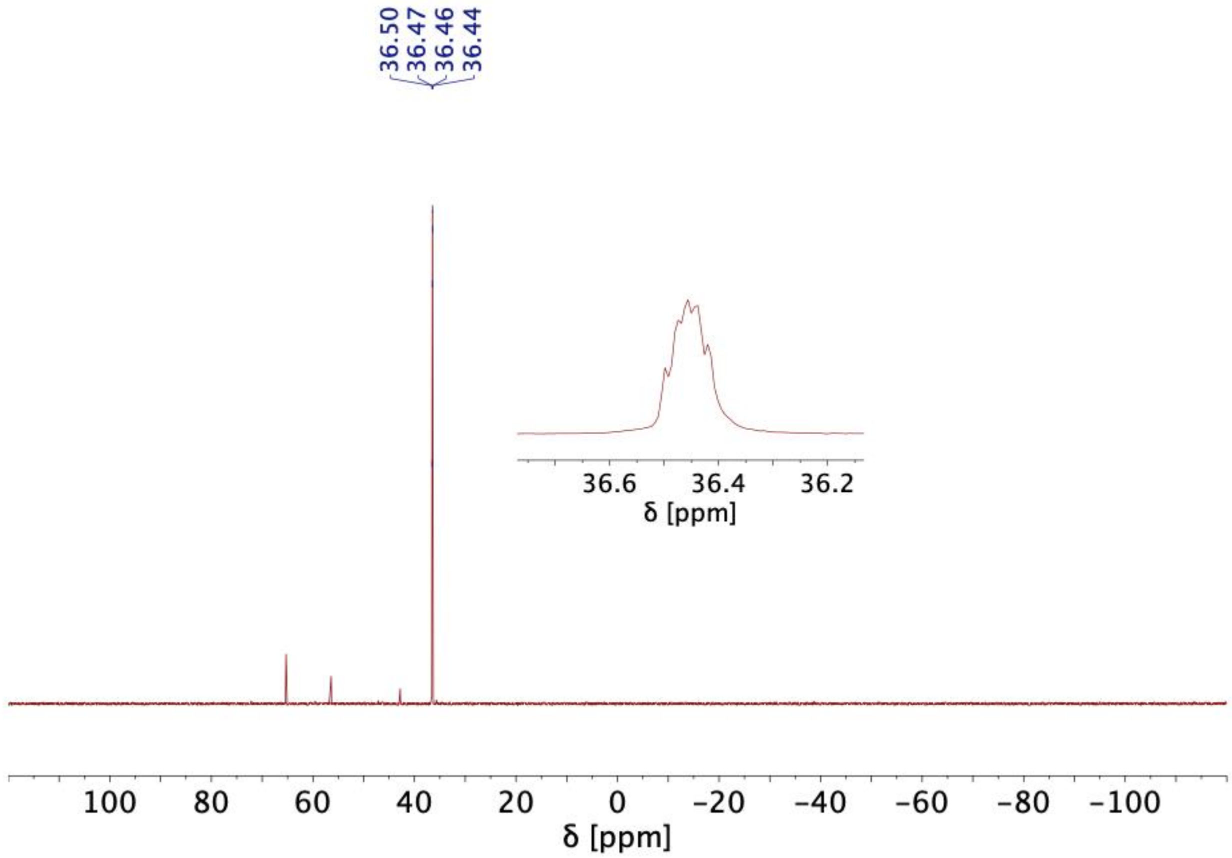

Figure S15: {^{1}H}^{31}P NMR spectrum (243 MHz, CD_3CN) of the reaction mixture obtained after 18 hours at 60 °C for the synthesis of N,N-diethyl-2,3,5,6-tetrafluoropyridin-4-amine (**12**), using the general procedure in section 1.11.

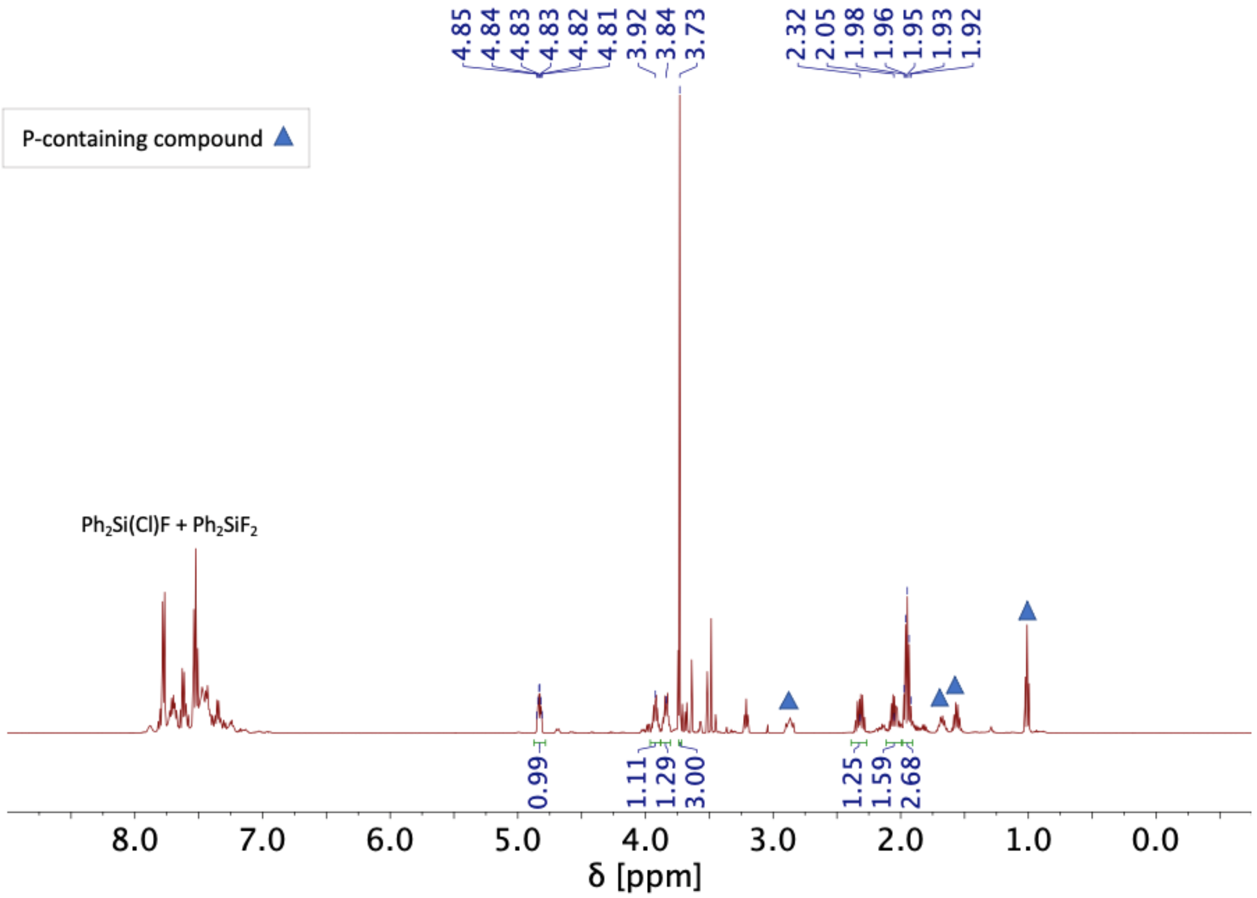

Figure S16: ^{1}H NMR spectrum (600 MHz, CD_3CN) of **13** obtained after 18 hours at 60 °C, using the general procedure in section 1.11.

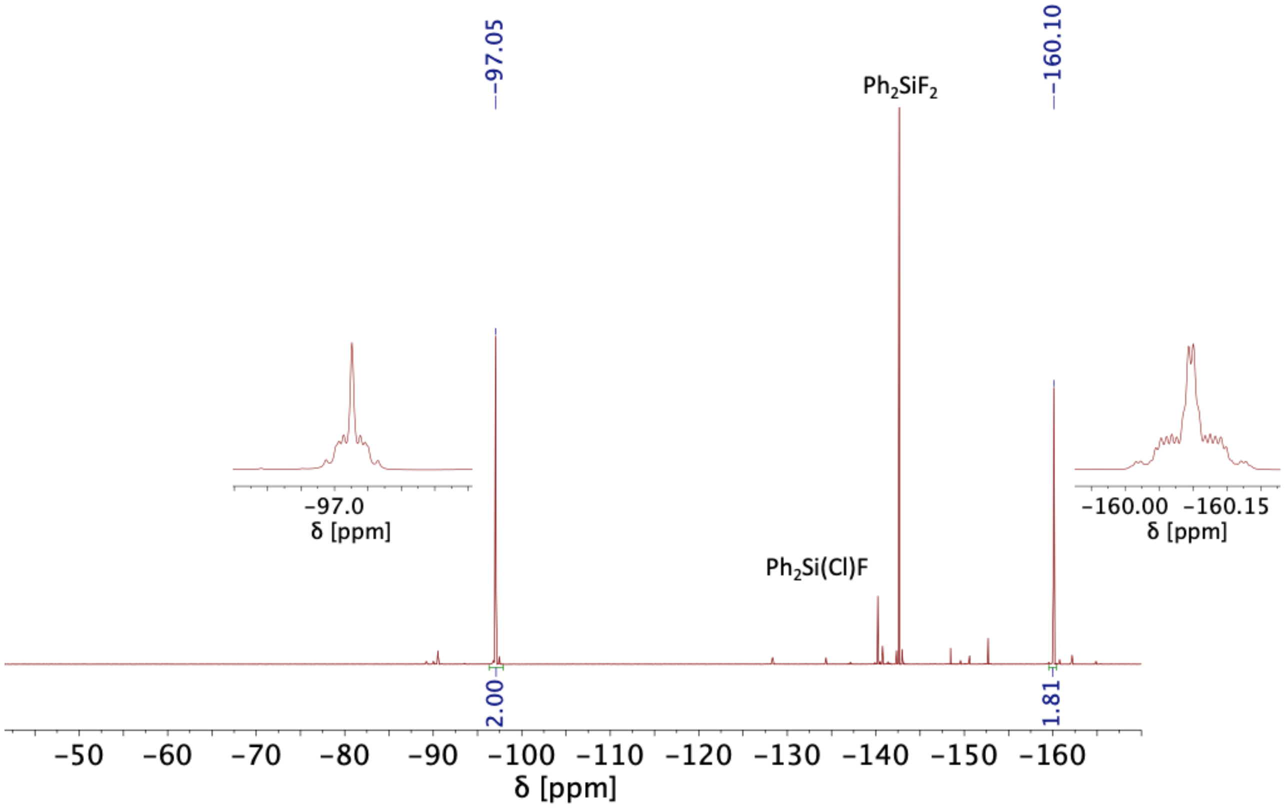

Figure S17: ^{19}F NMR spectrum (471 MHz, CD_3CN) of **13** obtained after 18 hours at 60 °C, using the general procedure in section 1.11.

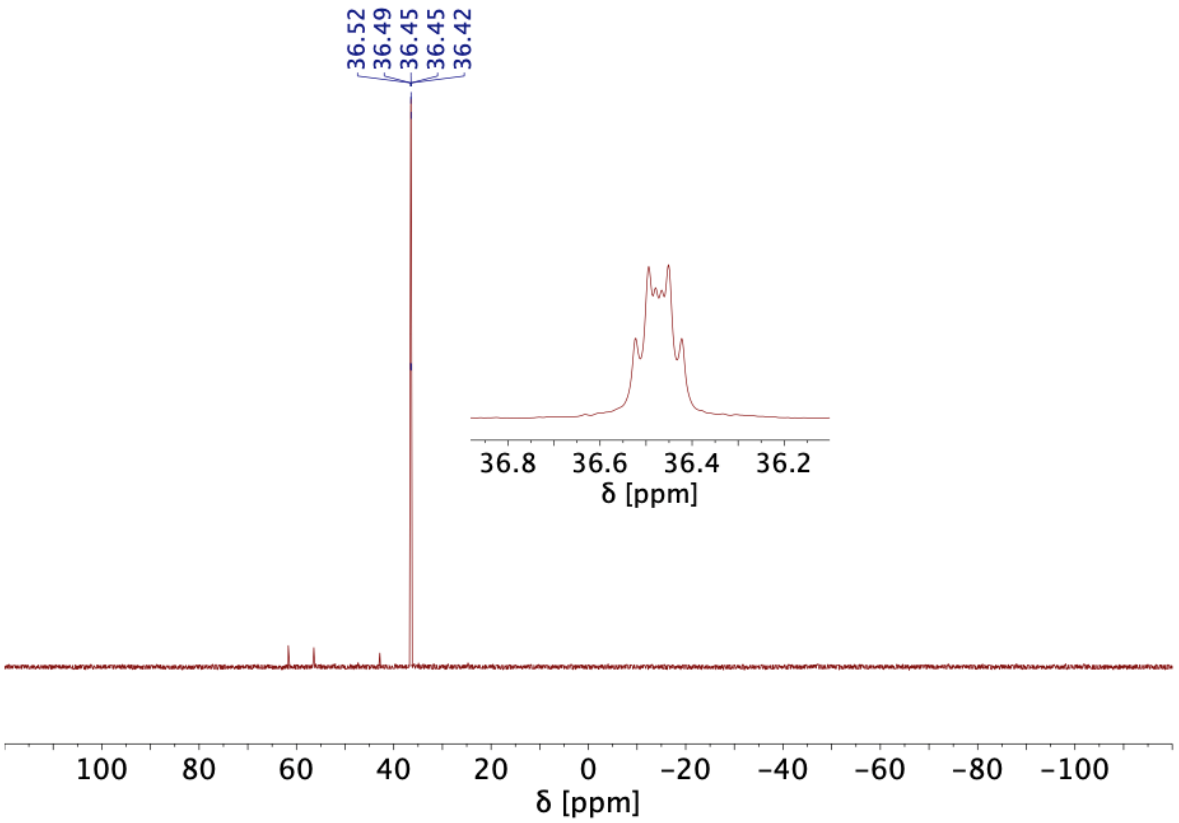

Figure S18: {^{1}H}^{31}P NMR spectrum (202 MHz, CD_3CN) of the reaction mixture obtained after 18 hours at 60 °C for the synthesis of **13**, using the general procedure in section 1.11.

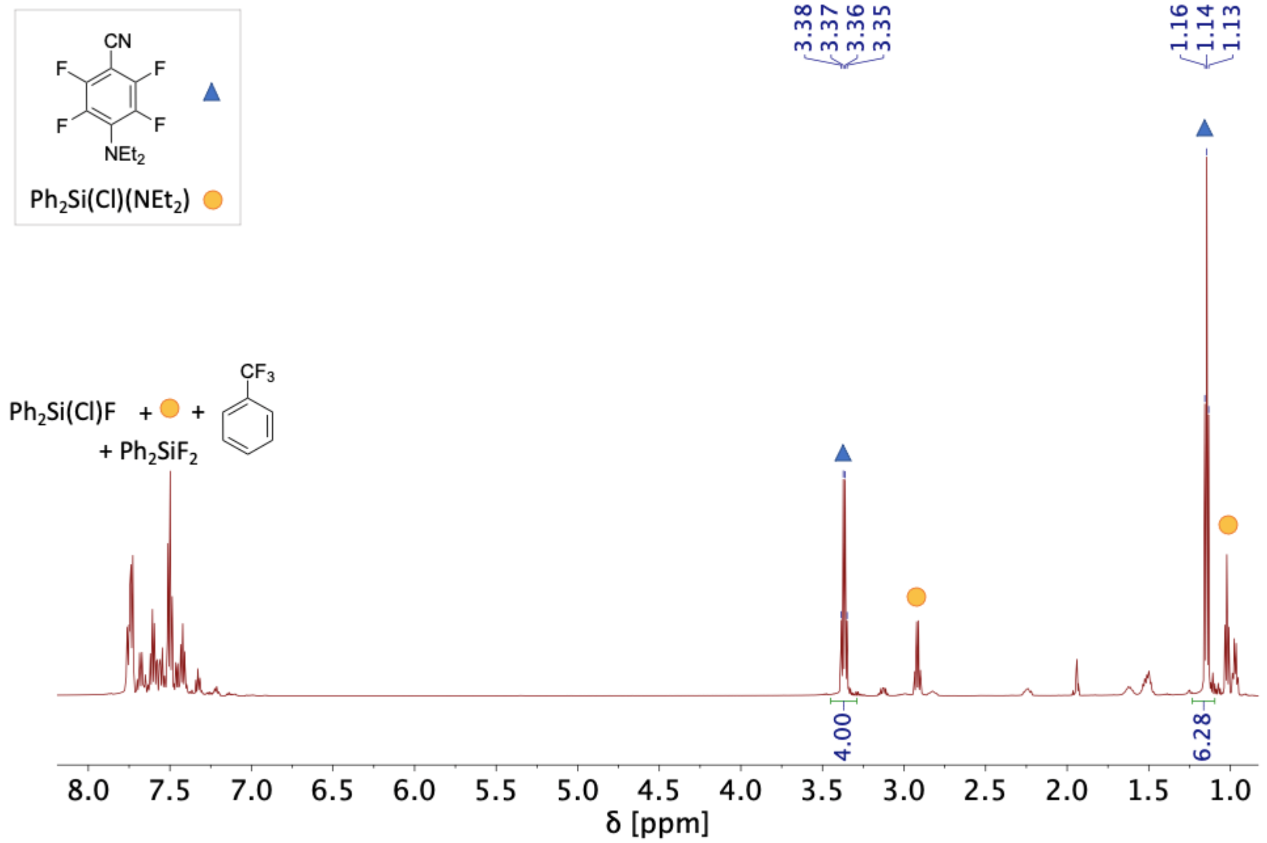

Figure S19: ^{1}H NMR spectrum (600 MHz, CD_3CN) of **14** obtained after 4 days at 60 °C followed by 1 day at 80 °C, using the general procedure in section 1.11.

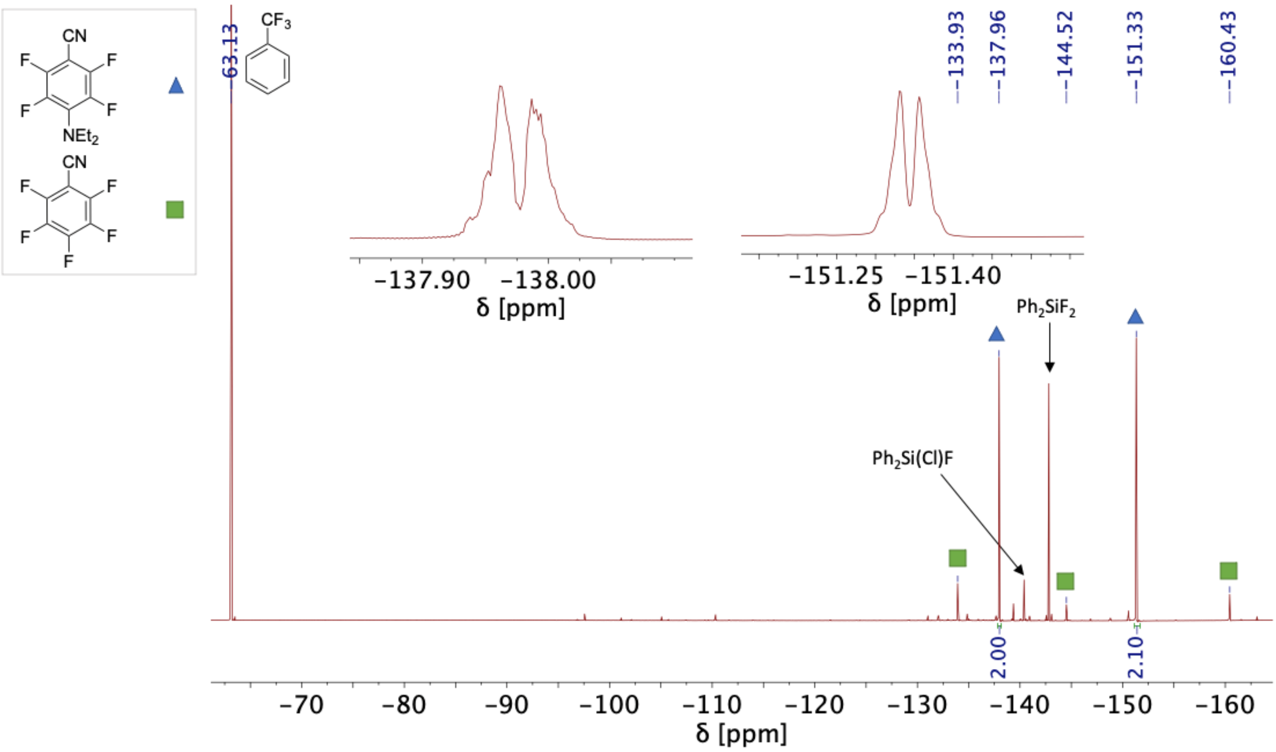

Figure S20: ^{19}F NMR spectrum (471 MHz, CD_3CN) of **14** obtained after 4 days at 60 °C followed by 1 day at 80 °C, using the general procedure in section 1.11.

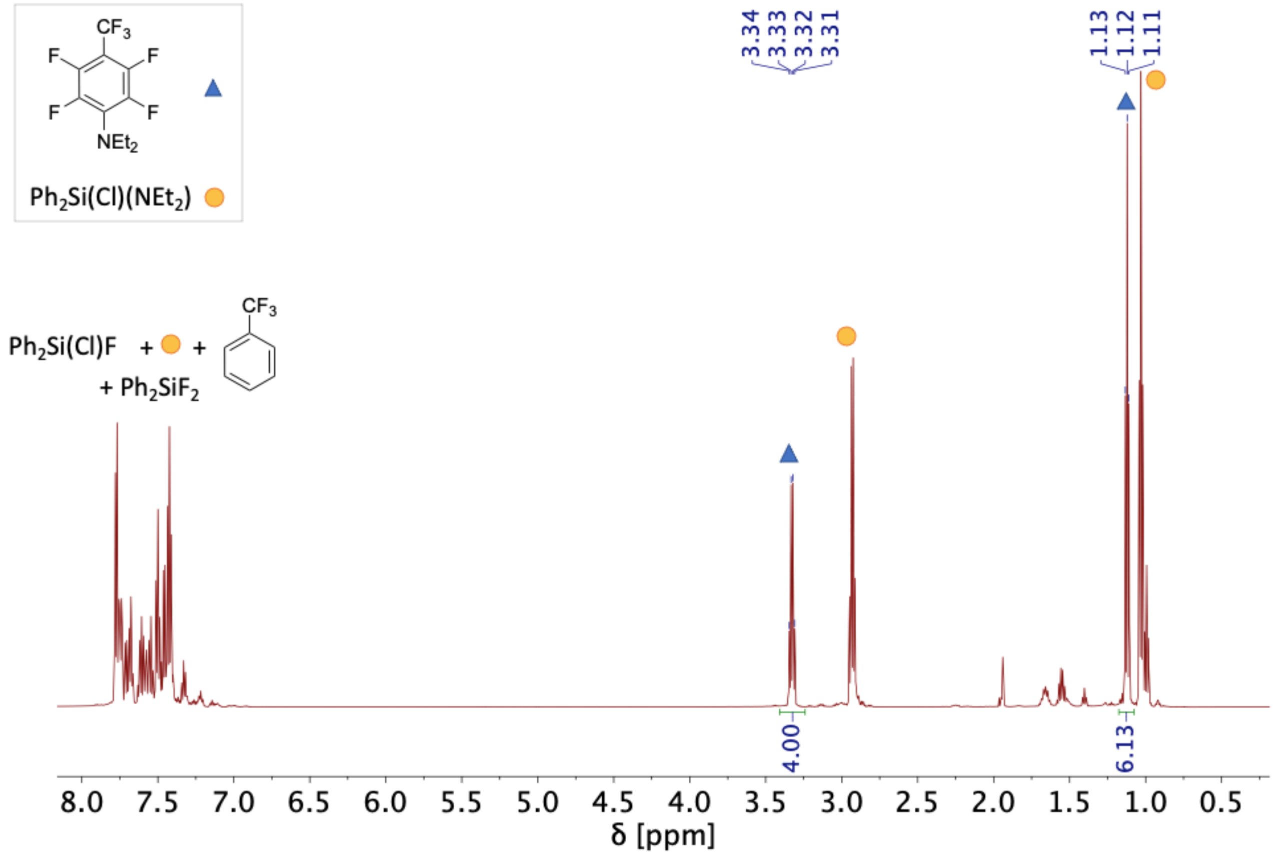

Figure S21: ^{1}H NMR spectrum (600 MHz, CD_3CN) of **15** obtained after 4 days at 60 °C followed by 6 days at 80 °C, using the general procedure in section 1.11.

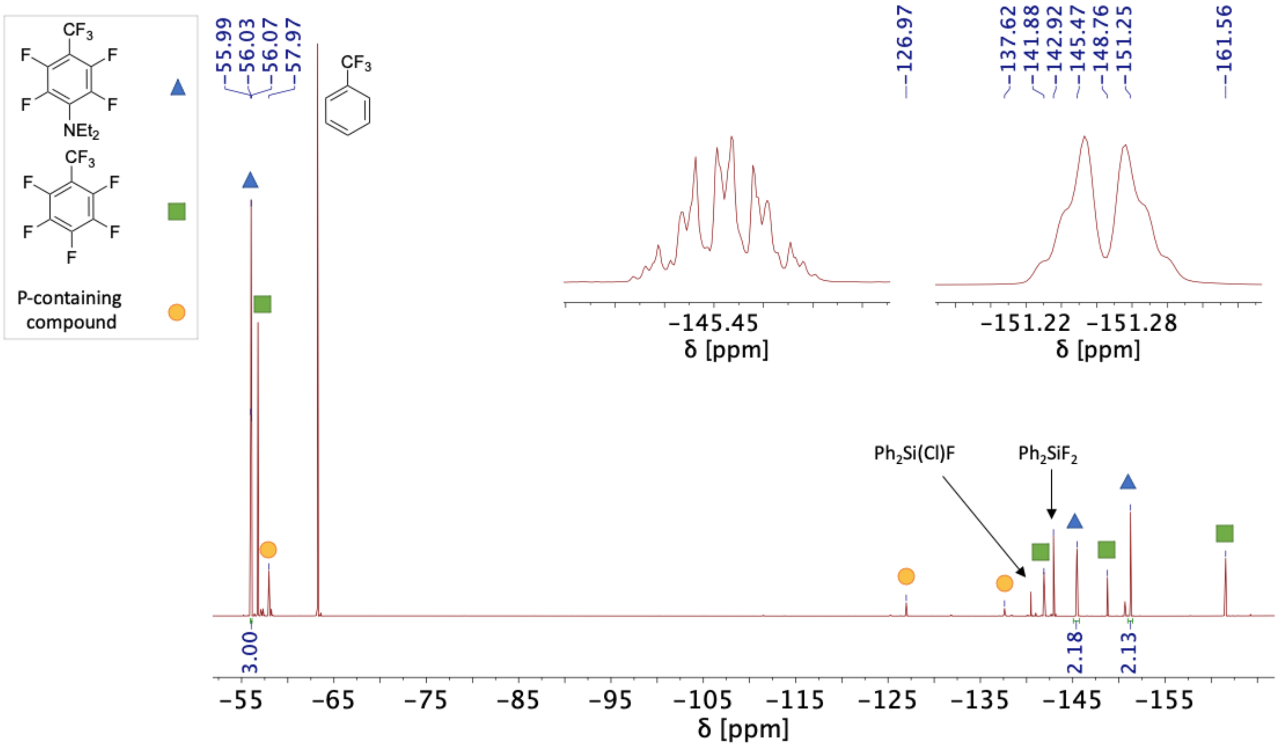

Figure S22: ^{19}F NMR spectrum (471 MHz, CD_3CN) of **15** obtained after 4 days at 60 °C followed by 6 days at 80 °C, using the general procedure in section 1.11.

1.12. Procedure for the stoichiometric synthesis of 3

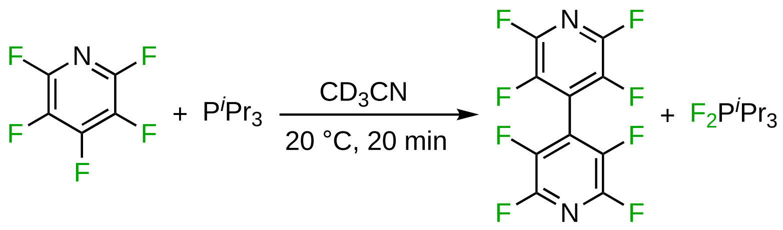

Pentafluoropyridine (33 µL, 0.30 mmol, 3.0 equivalents) and P^{i}Pr$_3$ (19 µL, 0.10 mmol, 1.0 equivalent) were dissolved in 0.4 mL of CD_3CN and placed into a J-Young NMR tube. The progress of reaction was monitored by ^{19}F NMR spectroscopy.

Perfluoro 4,4'-bipyridine (3):[11] Yield of 84%. ^{19}F NMR (565 MHz, CD_3CN): δ –91.04 (mc, 4F), –139.84 (mc, 4F). GC-MS(EI) $[C_{10}N_2F_8]^{+\cdot}$ 299.99075 *m/z* (6.92 ppm deviation from theoretical mass).

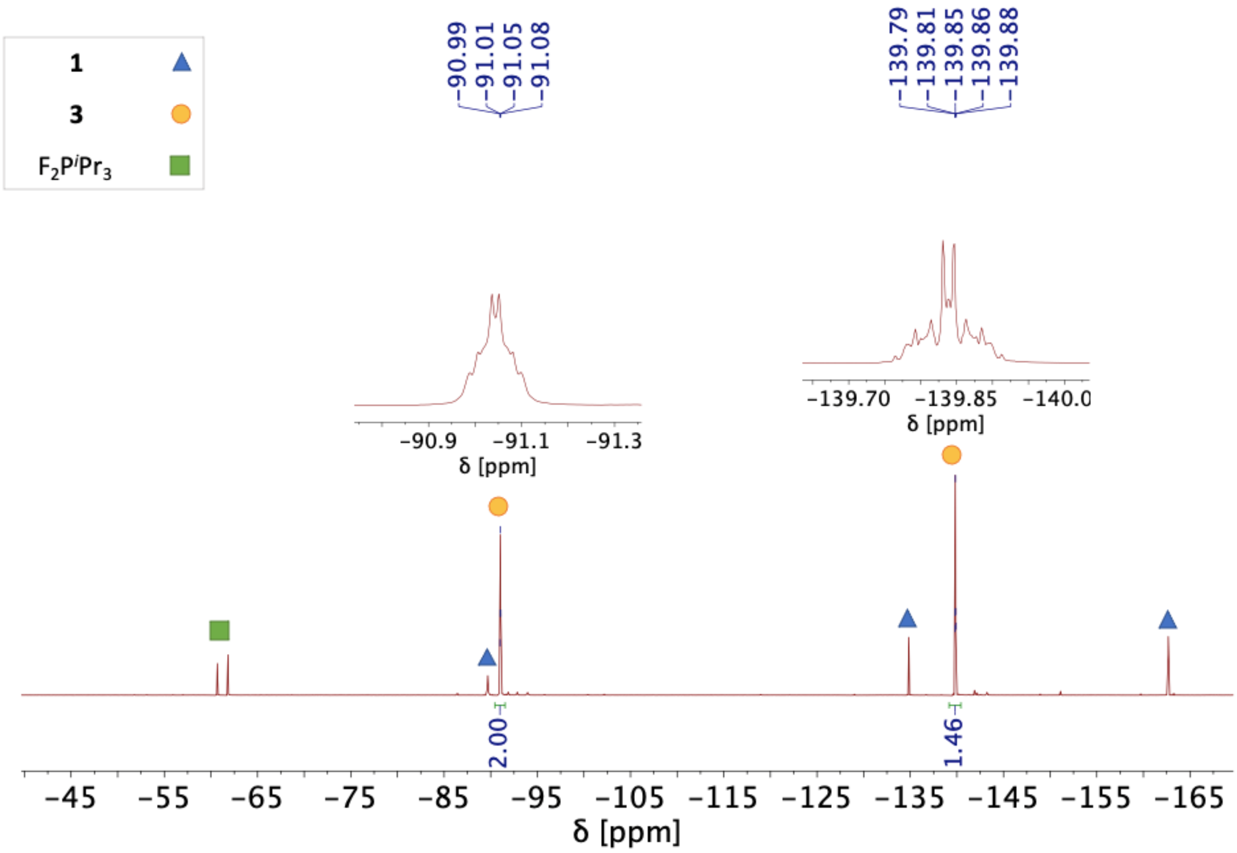

Figure S23: ^{19}F NMR spectrum (565 MHz, CD_3CN) of **3** obtained after 30 minutes at 20 °C.

2. Mechanistic Studies

2.1. Reactivity test of $PhSiH_3$ and pentafluoropyridine (1)

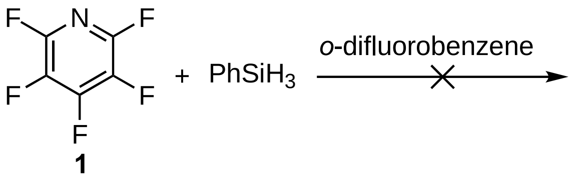

Pentafluoropyridine (44 µL, 0.40 mmol, 1.0 equivalent) and $PhSiH_3$ (49 µL, 0.40 mmol, 1.0 equivalent) were dissolved in 0.4 mL of *o*-difluorobenzene and placed into a J-Young NMR tube. The mixture was heated at 60 °C for 18 hours and monitored by ^{19}F NMR spectroscopy, which demonstrated that no reaction occurred.

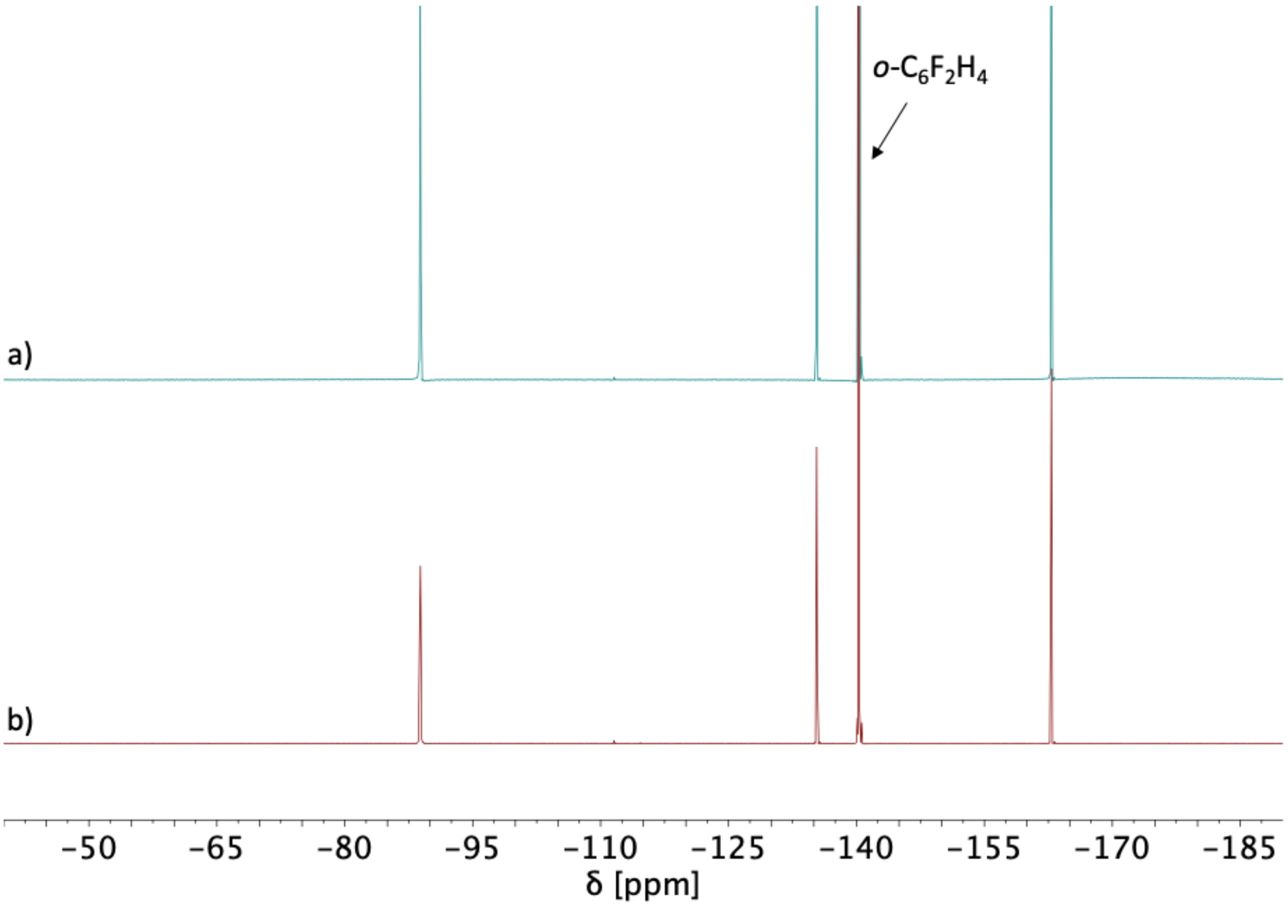

Figure S24 ^{19}F NMR spectra (565 MHz, *o*-difluorobenzene) of the system at t = 0 (spectrum a)) and after 18 hours at 60 °C (spectrum b)).

2.2. Reactivity test of Ph_2SiH_2 and P^nBu_3

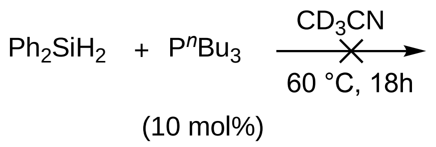

P^nBu_3 (10 µL, 0.040 mmol, 0.10 equivalent) and Ph_2SiH_2 (44 µL, 0.40 mmol, 1.0 equivalent) were dissolved in 0.4 mL of CD_3CN and placed into a J-Young NMR tube. The mixture was heated at 60 °C for 18 hours and monitored by ^1H and ^{31}P NMR spectroscopy, which demonstrated that no reaction occurred.

.

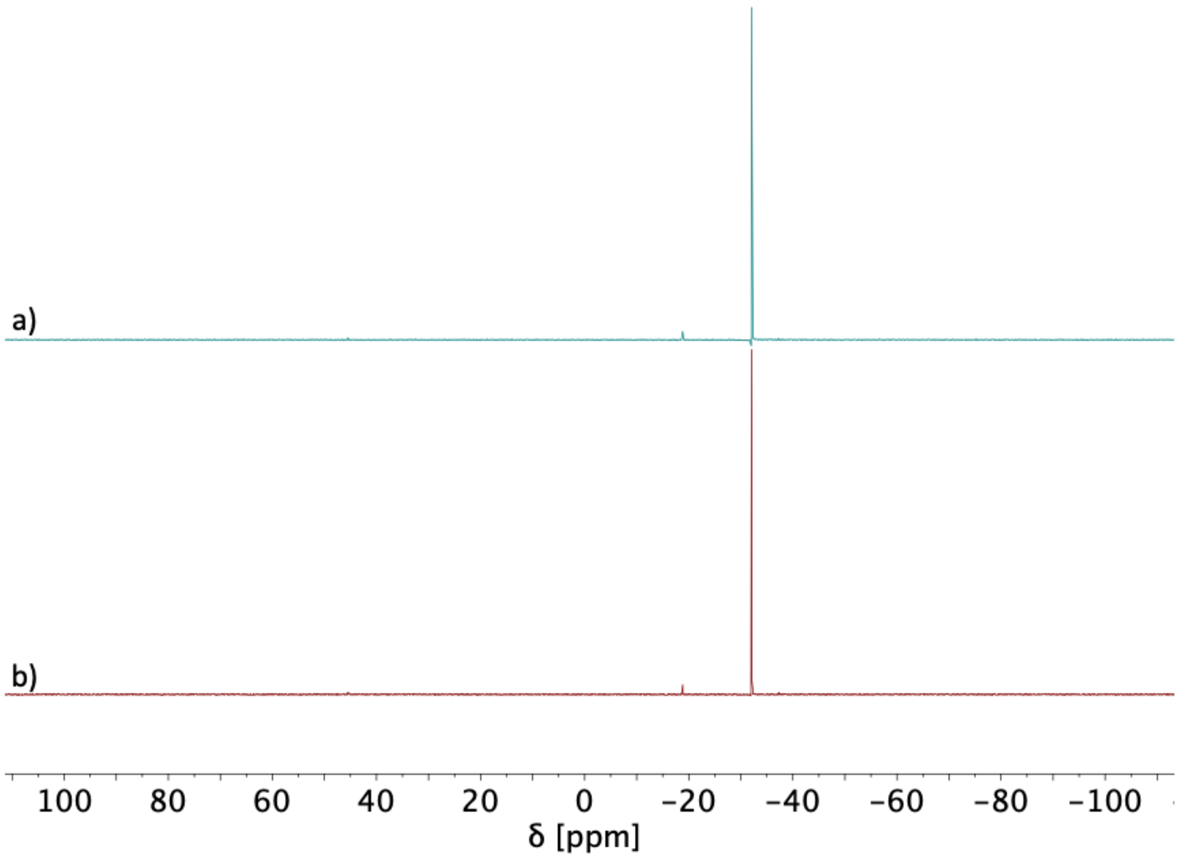

Figure S25 $\{^1H\}^{31}P$ NMR (243 MHz, CD_3CN) spectra of the system at t = 0 (spectrum a)) and after 18 hours at 60 °C (spectrum b)).

2.3. Reactivity test of Ph_2SiH_2 and pentafluoropyridine

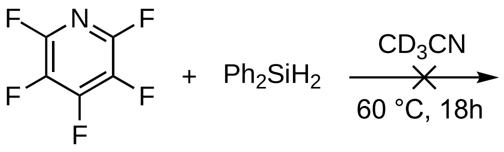

(1 equiv.) (1 equiv.)

1

Pentafluoropyridine (44 µL, 0.40 mmol, 1.0 equivalent) and Ph_2SiH_2 (44 µL, 0.40 mmol, 1.0 equivalent) were dissolved in 0.4 mL of CD_3CN and placed into a J-Young NMR tube. The mixture was heated at 60 °C for 18 hours and monitored by ^{1}H and ^{19}F NMR spectroscopy, which demonstrated that no reaction occurred.

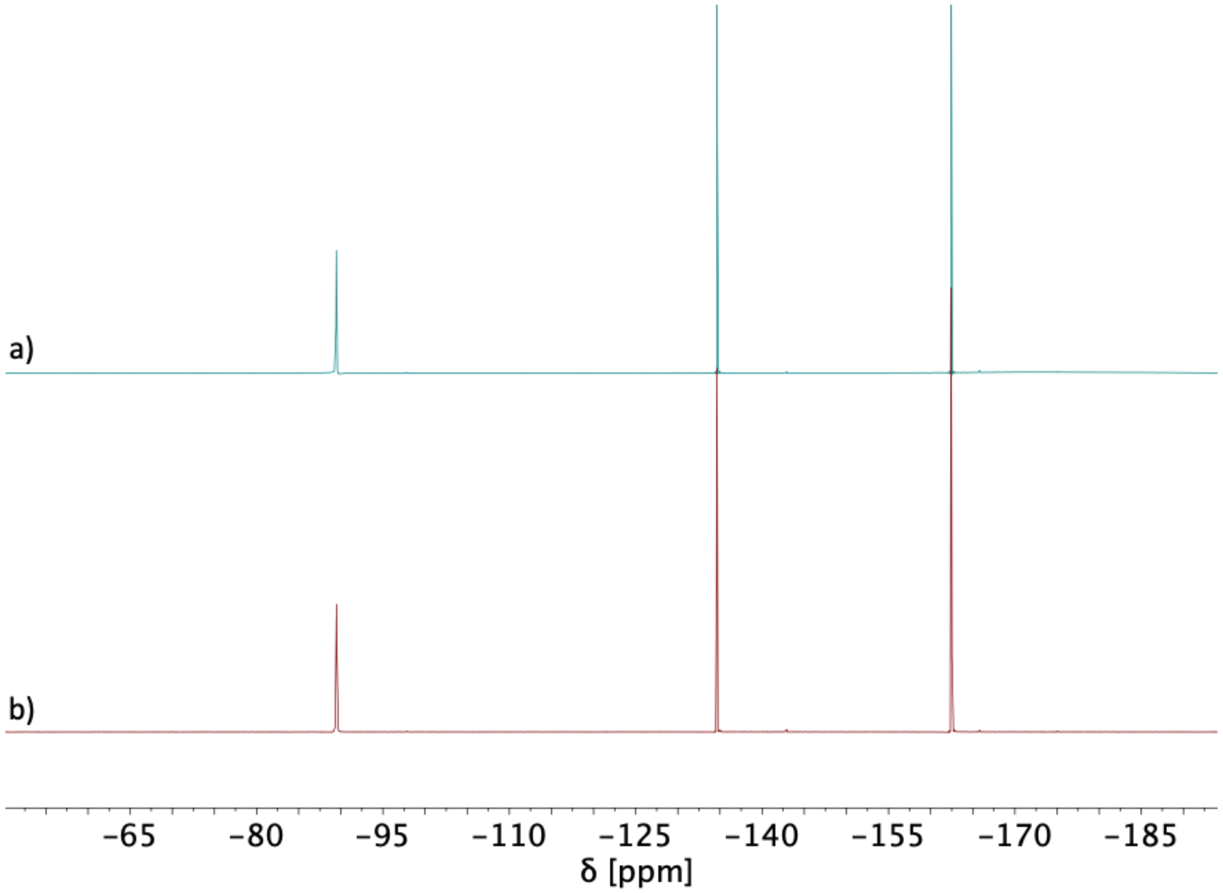

Figure S26 ^{19}F NMR spectra (565 MHz, CD_3CN) of the system at t = 0 (spectrum a)) and after 18 hours at 60 °C (spectrum b)).

2.4. Reactivity test of Ph_2SiH_2 and pentafluoropyridine in the presence of $[Me_4N]F$

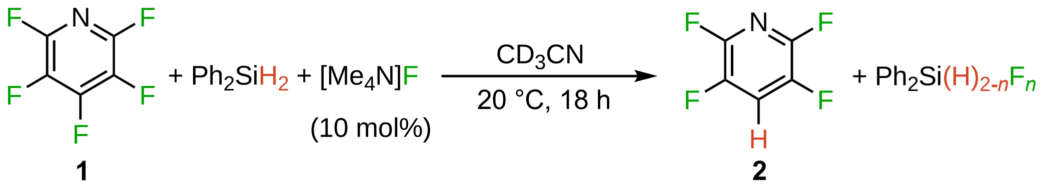

Pentafluoropyridine (44 µL, 0.40 mmol, 1.0 equivalent), Ph_2SiH_2 (44 µL, 0.40 mmol, 1.0 equivalent) and anhydrous Me_4NF (3.7 mg, 0.040 mmol, 0.10 equivalent) were dissolved in 0.4 mL of CD_3CN and placed into a J-Young NMR tube. The mixture was kept at 20 °C for 18 hours and monitored by ^{1}H and ^{19}F NMR spectroscopy. The yields are lower (19% after 20 minutes, 64% after 18 hours) than those of the reaction in the presence of $P^{n}Bu_3$ (Table 1, entry 6, yield 93% after 20 minutes at 20 °C). Hence, direct hydrodefluorination of **1** by catalytic hydrosilicate anions, formed *in situ* by reaction of **1** with $P^{n}Bu_3$, is possible, but significantly slower than hydrodefluorination through a phosphine-mediated pathway.

2.5. Characterisation of phosphonium salt [17][PhSi(H)$_{4\text{-}n}$F$_n$]

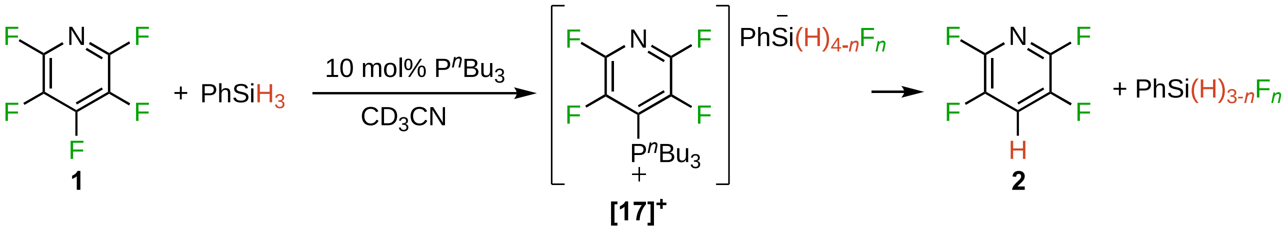

Pentafluoropyridine (44 µL, 0.40 mmol, 1.0 equivalent) and $PhSiH_3$ (50 µL, 0.40 mmol, 1.0 equivalent) were dissolved in 0.4 mL of the tested solvent and placed into a J-Young NMR tube. $P^{n}Bu_3$ (9.9 µL, 0.10 mmol) was added and the progress of the reaction was monitored after 1 hour at 20 °C and 2 hours at 60 °C. The reaction mixture was analysed by ^{1}H, ^{31}P and quantitative ^{19}F NMR with trifluorotoluene (25 µL, 0.20 mmol, 0.50 equivalent) used as an internal standard, as described in section 1.7.

The ^{31}P NMR spectrum showed the clean conversion of $P^{n}Bu_3$ into a new species **[17]**$^{+}$ after 1 hour at 20 °C.

[17]$^{+}$: ^{1}H NMR (600 MHz, CD_3CN): δ 2.68 (td, *J* = 12.7, 8.4 Hz, 6H), 1.69 – 1.59 (m, 6H), 1.54 (h, *J* = 7.1 Hz, 6H), 0.99 (t, $^{3}J_{HH}$ = 7.3 Hz, 9H). {^{1}H}^{31}P NMR (243 MHz, CD_3CN): δ 38.11 (mc, 1P). ^{19}F NMR (565 MHz, CD_3CN): δ –88.48 (mc, 2F), –130.32 (mc, 2F).

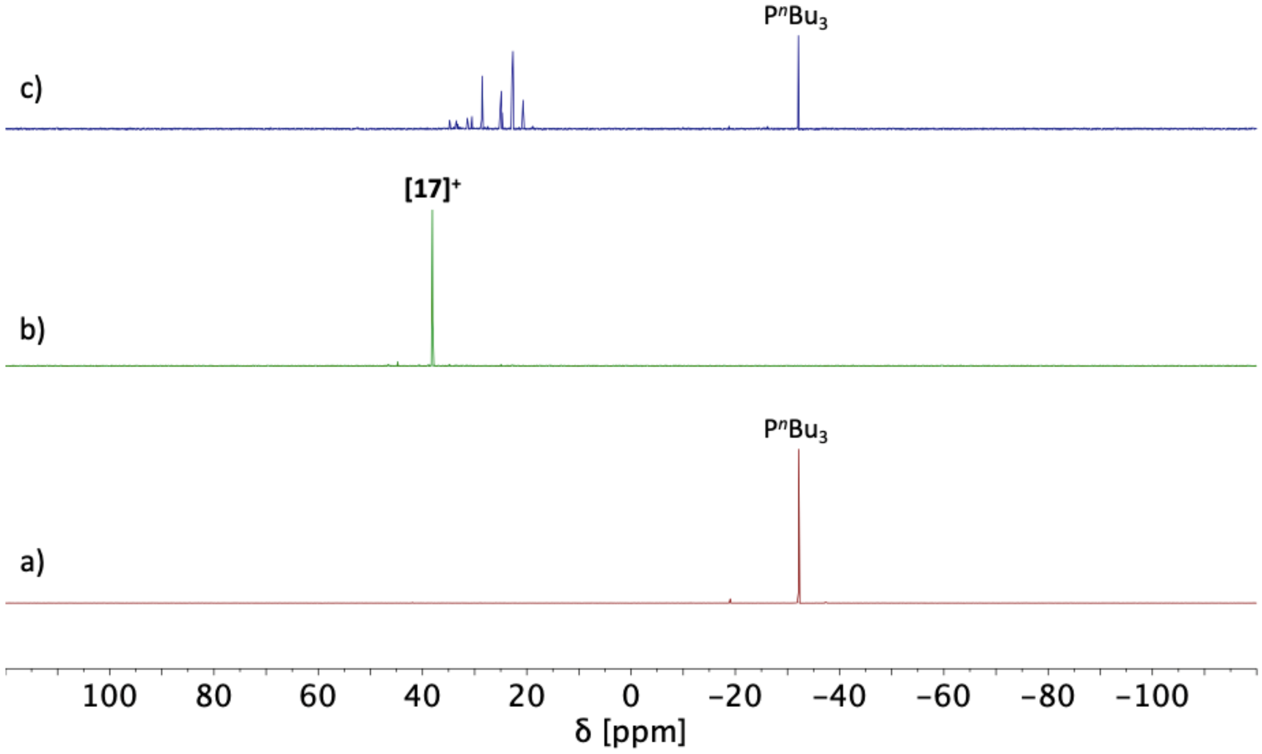

Figure S27 {^{1}H}^{31}P NMR (243 MHz, CD_3CN) spectra of the system at t = 0 (spectrum a)), after 1 hour at 20 °C (spectrum b)) and after 2 hours at 60 °C (spectrum c)).

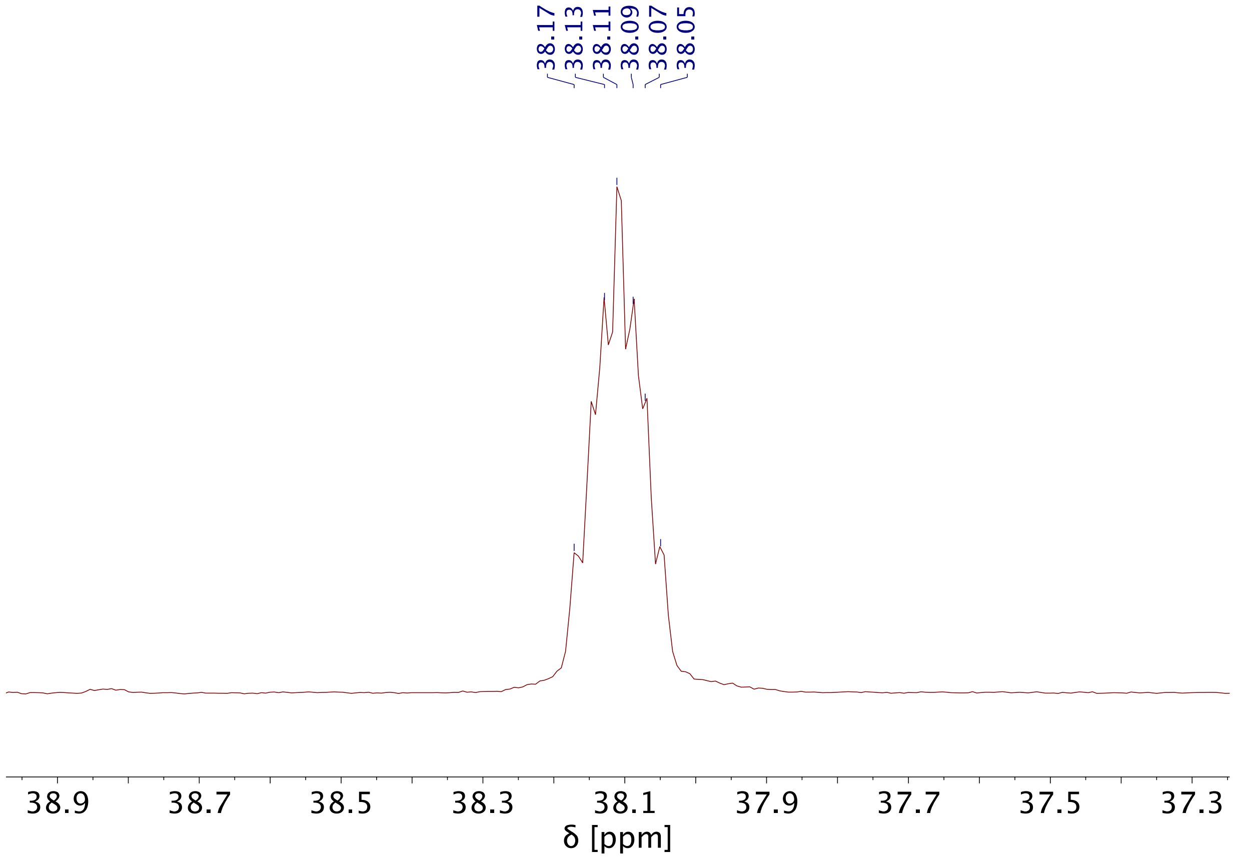

Figure S28 {^{1}H}^{31}P NMR (243 MHz, CD_3CN) spectrum of the system after 1 hour at 20 °C (species **[17]$^+$**).

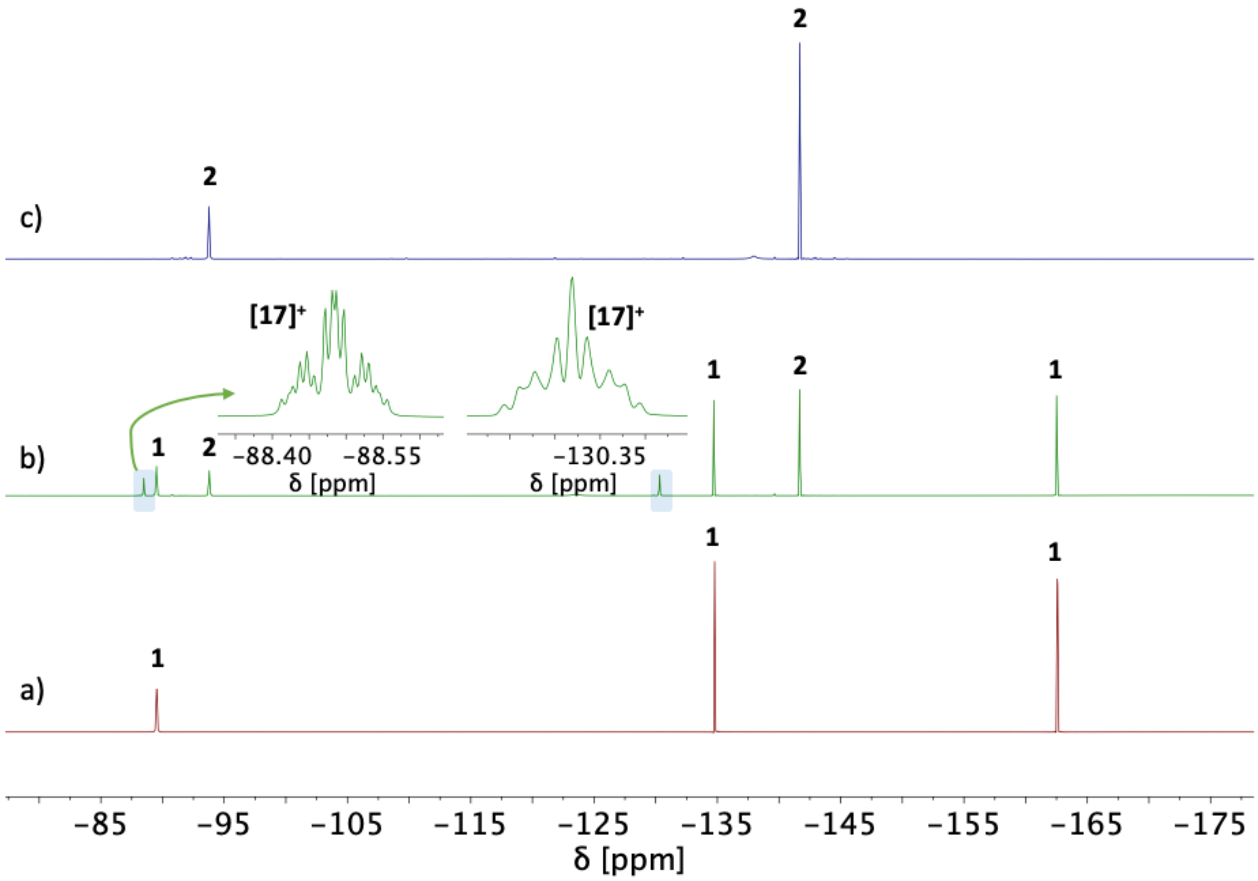

Figure S29 ^{19}F NMR (565 MHz, CD_3CN) spectra of the system at t = 0 (spectrum a)), after 1 hour at 20 °C (spectrum b)) and after 2 hours at 60 °C (spectrum c)).

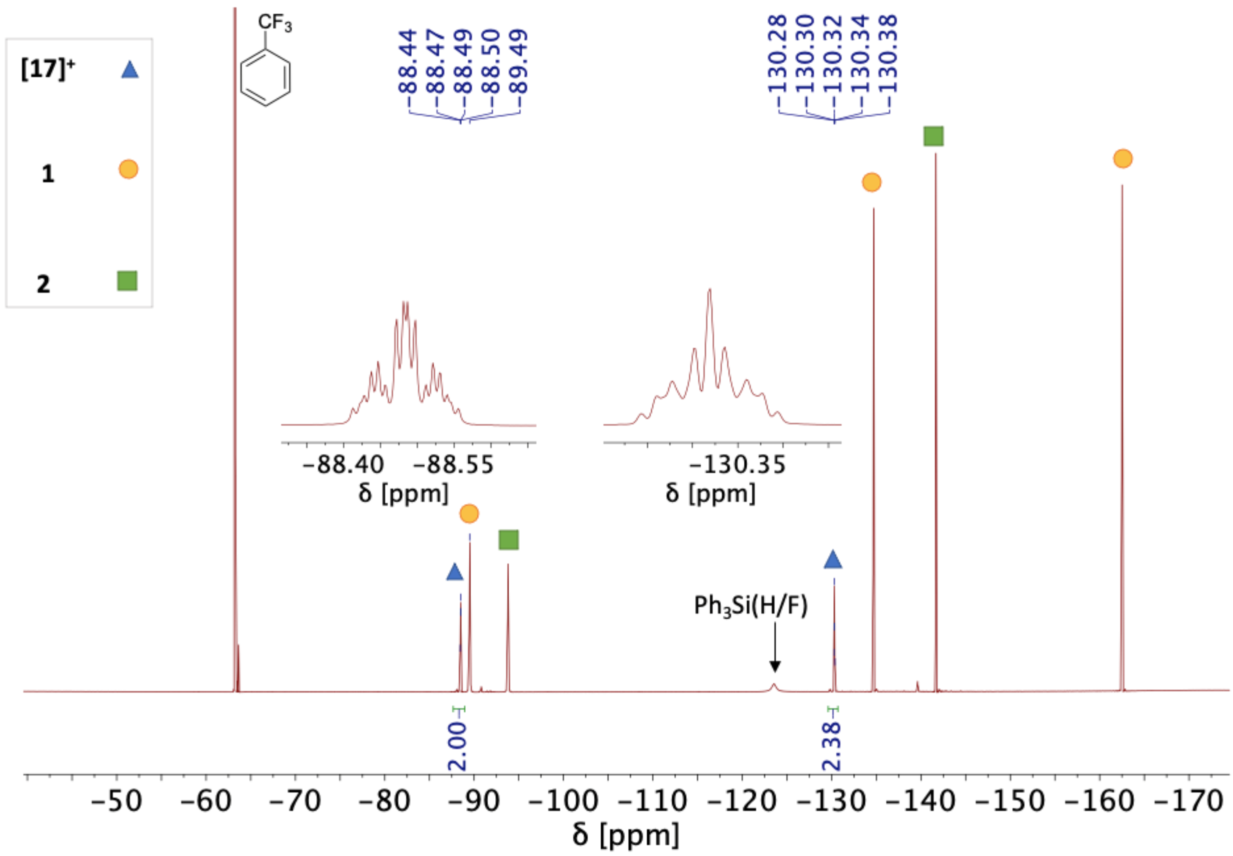

Figure S30 ^{19}F NMR (565 MHz, CD_3CN) spectrum of the system after 1 hour at 20 °C.

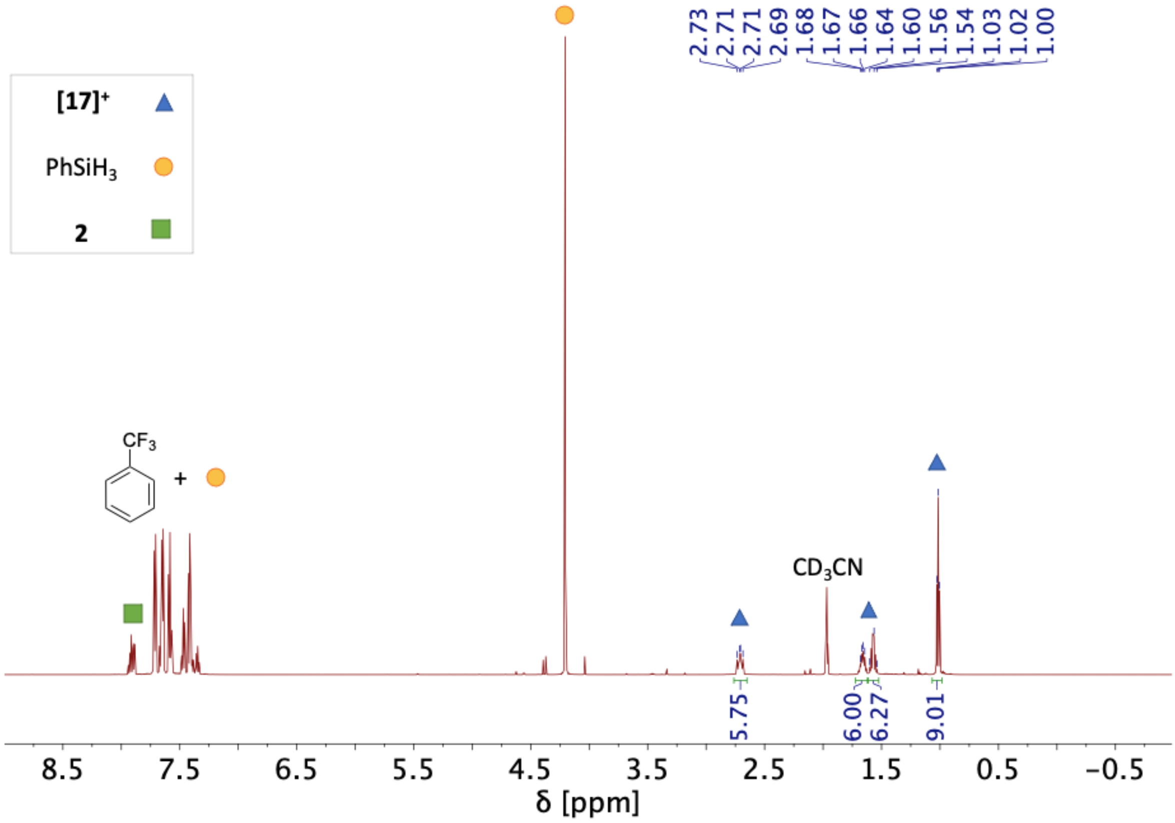

Figure S31 ^{1}H NMR (600 MHz, CD_3CN) spectrum of the system after 1 hour at 20 °C (species **[17]**$^{+}$).

2.6. Characterisation and reactivity of phosphonium salt [17]Br

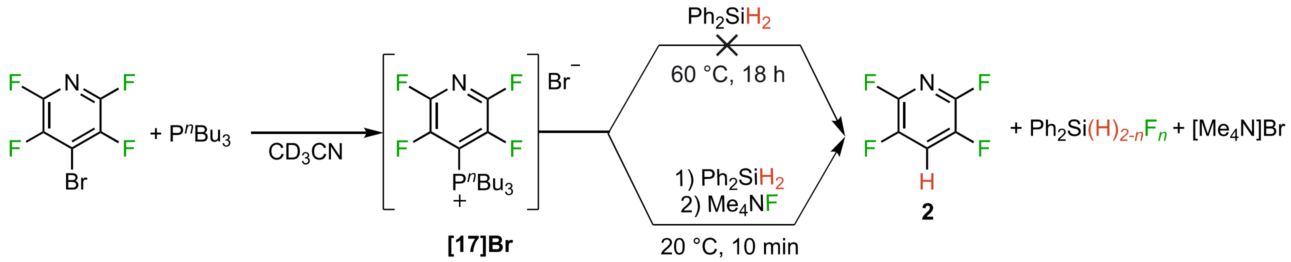

Pentafluoropyridine (22 µL, 0.20 mmol, 1.0 equivalent) and P^{n}Bu$_3$ (49 µL, 0.20 mmol, 1.0 equivalent) were dissolved in 0.4 mL of CD_3CN and placed into a J-Young NMR tube. The mixture turned immediately brown and ^{1}H, ^{19}F and ^{31}P NMR analyses showed the formation of **[17]Br**. Heating the system at 40 °C for 18 hours did not make any change in the reaction mixture. Ph_2SiH_2 (37 µL, 0.20 mmol, 1.0 equivalent) was added and the system was heated at 40 °C for other 18 hours. ^{1}H, ^{19}F and ^{31}P NMR analyses demonstrated that no further reaction occurred. The addition of anhydrous [Me$_4$N]F (20 mg, 0.20 mmol, 1.0 equivalent) induced the formation of a white solid corresponding to [Me$_4$N]Br, **2**, fluorosilane $Ph_2Si(H)_{2-n}F_n$ and some unreacted **[17]Br**.

[17]Br: ^{1}H NMR (500 MHz, CD_3CN): δ 2.92 (td, *J* = 13.0, 8.5 Hz, 6H), 1.66 (dq, *J* = 16.5, 8.5 Hz, 6H), 1.52 (h, *J* = 7.3 Hz, 6H), 0.96 (t, *J* = 7.3 Hz, 9H). {^{1}H}^{31}P NMR (202 MHz, CD_3CN): δ 37.87 (mc, 1P). ^{19}F NMR (471 MHz, CD_3CN): δ –89.23 (mc, 2F, *ortho*-F), –130.29 (mc, 2F, *meta*-F).

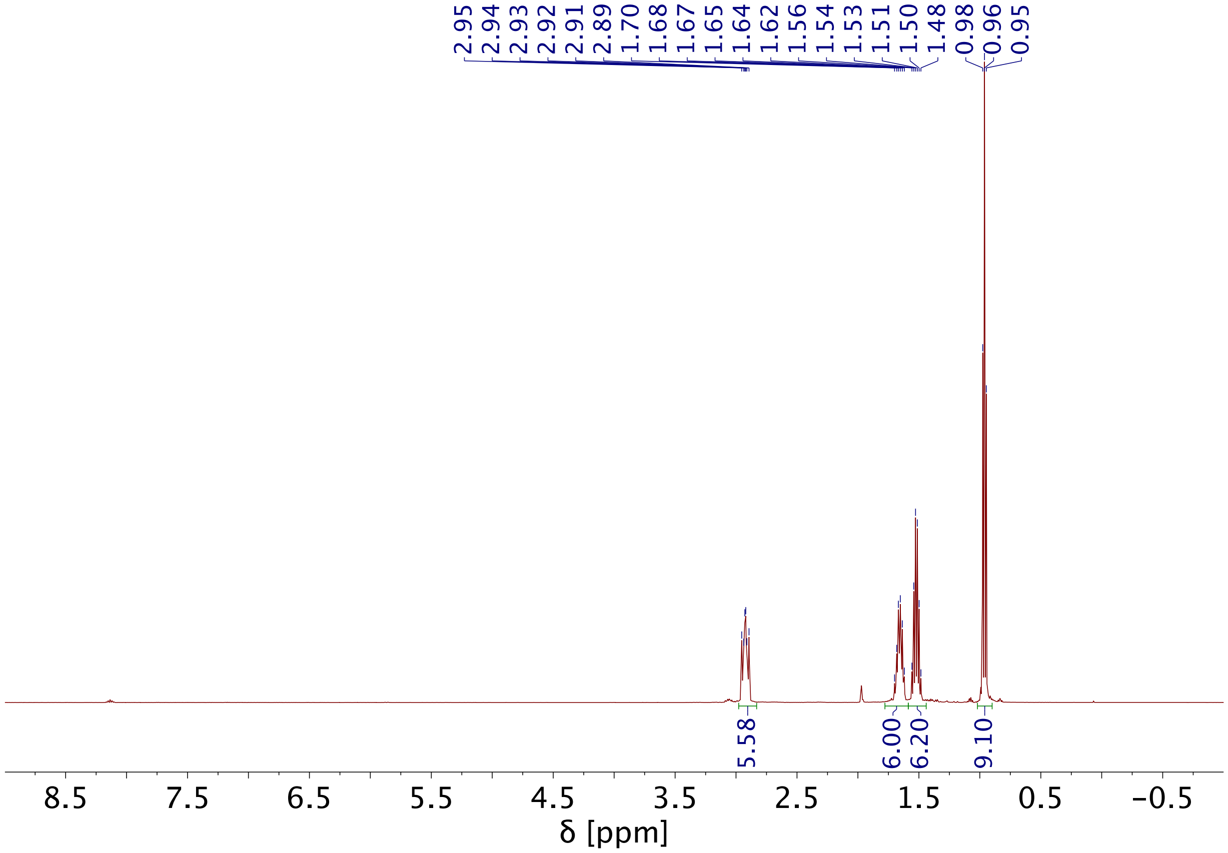

Figure S32 ^{1}H NMR (500 MHz, CD_3CN) spectrum of the system after 18 hours at 40 °C.

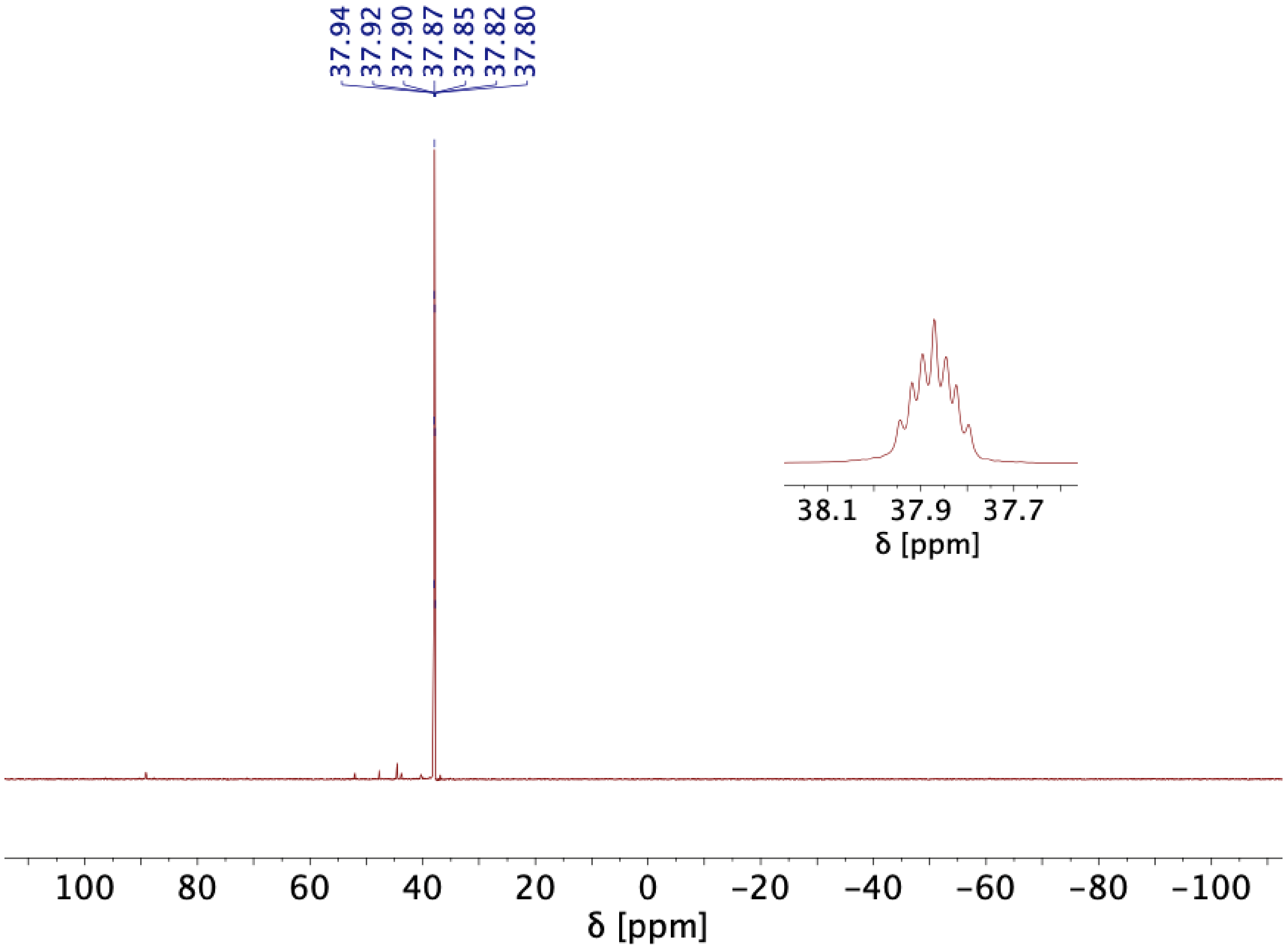

Figure S33 {^{1}H}^{31}P NMR (202 MHz, CD_3CN) spectrum of the system after 18 hours at 40 °C.

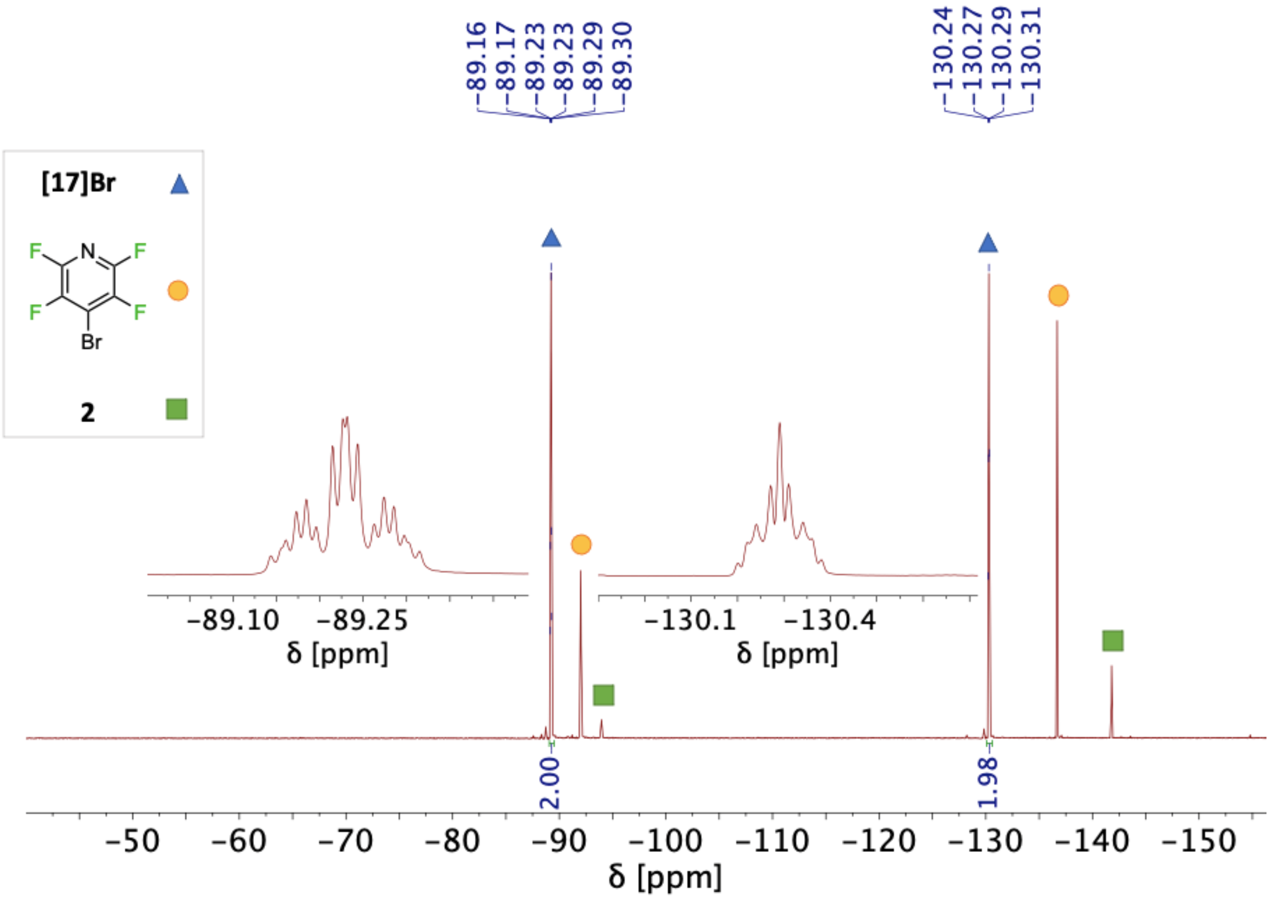

Figure S34 ^{19}F NMR (471 MHz, CD_3CN) spectrum of the system after 18 hours at 40 °C.

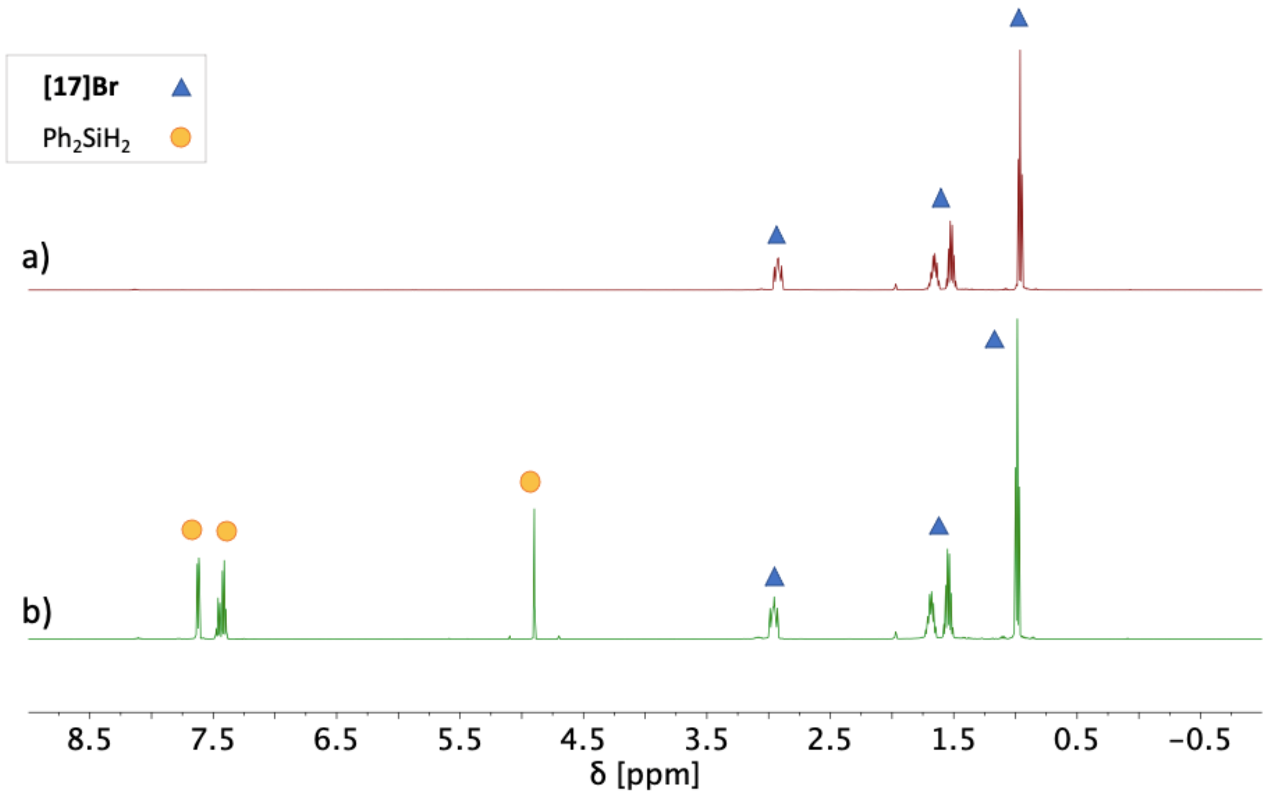

Figure S35 ^{1}H NMR (500 MHz, CD_3CN) spectra of the system after 18 hours at 40 °C (spectrum a)) and after 18 hours at 40 °C from the addition of Ph_2SiH_2 (spectrum b)).

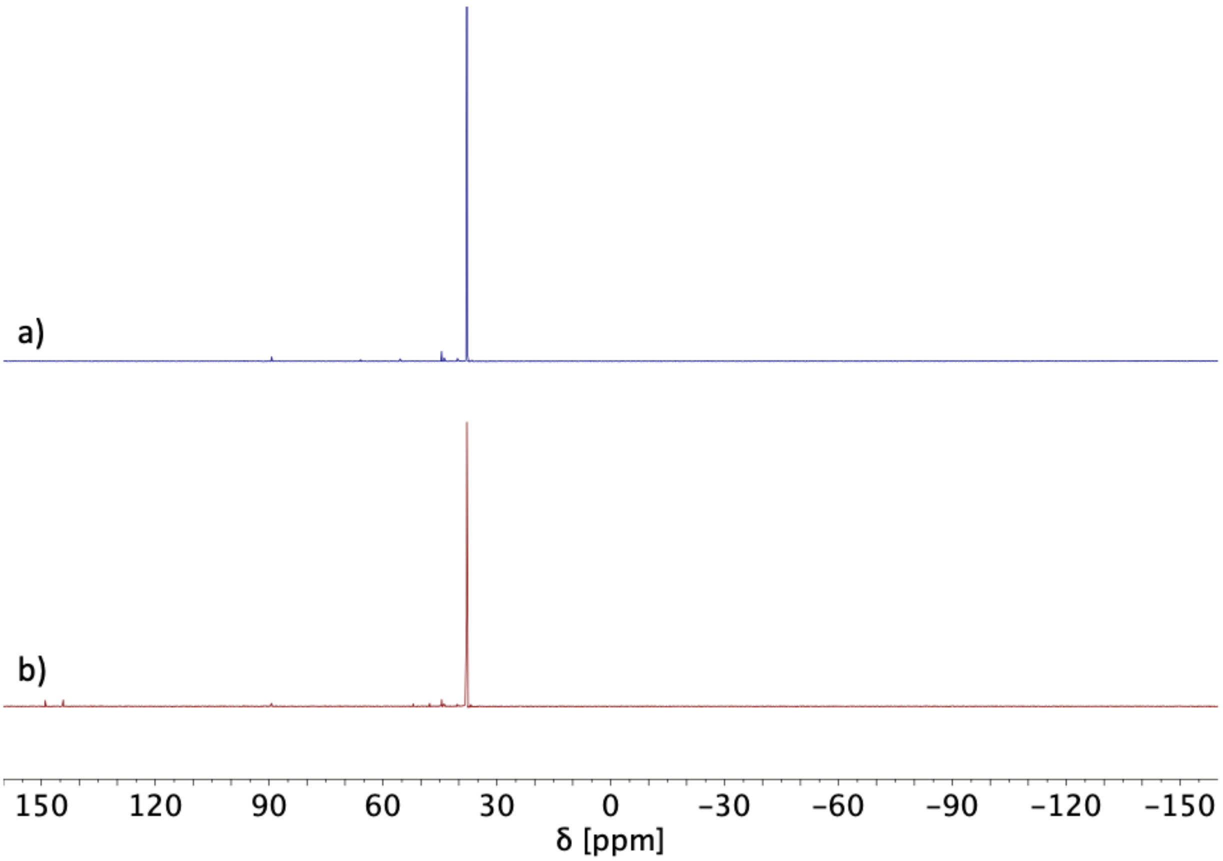

Figure S36 {^{1}H}^{31}P NMR (202 MHz, CD_3CN) spectra of the system after 18 hours at 40 °C (spectrum a)) and after 18 hours at 40 °C from the addition of Ph_2SiH_2 (spectrum b)).

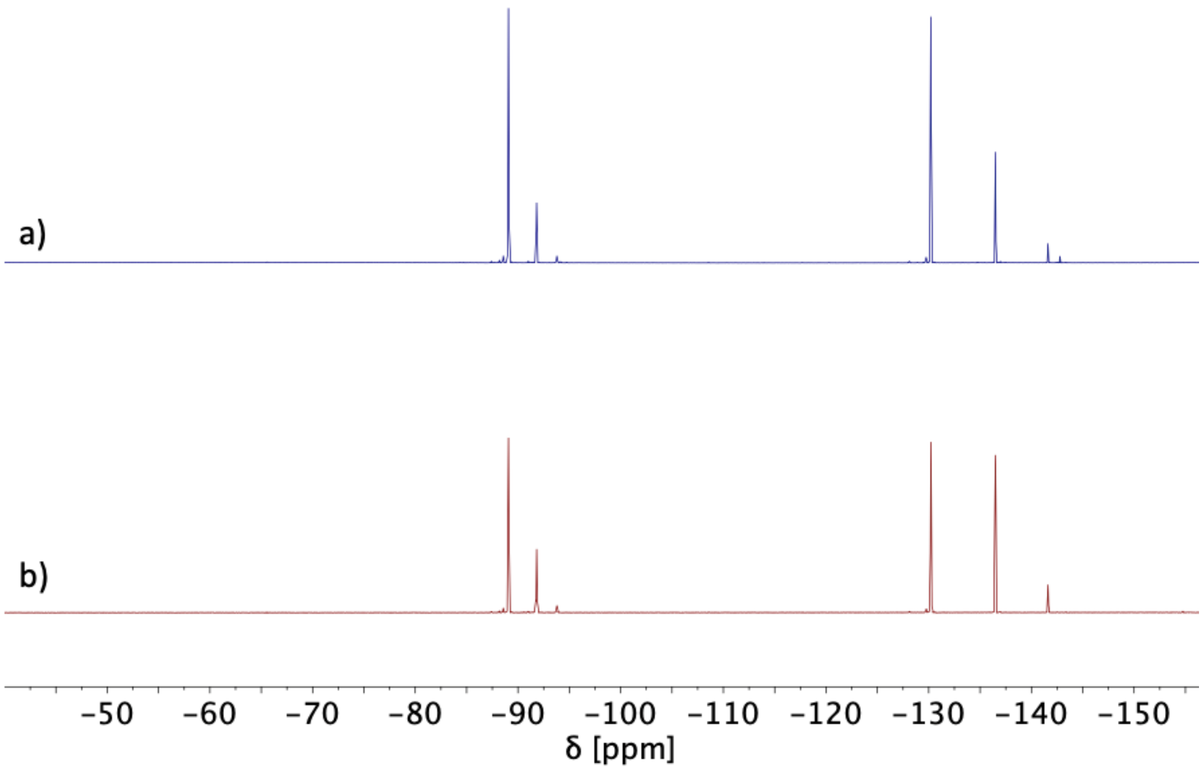

Figure S37 ^{19}F NMR (471 MHz, CD_3CN) spectra of the system after 18 hours at 40 °C (spectrum a)) and after 18 hours at 40 °C from the addition of Ph_2SiH_2 (spectrum b)).

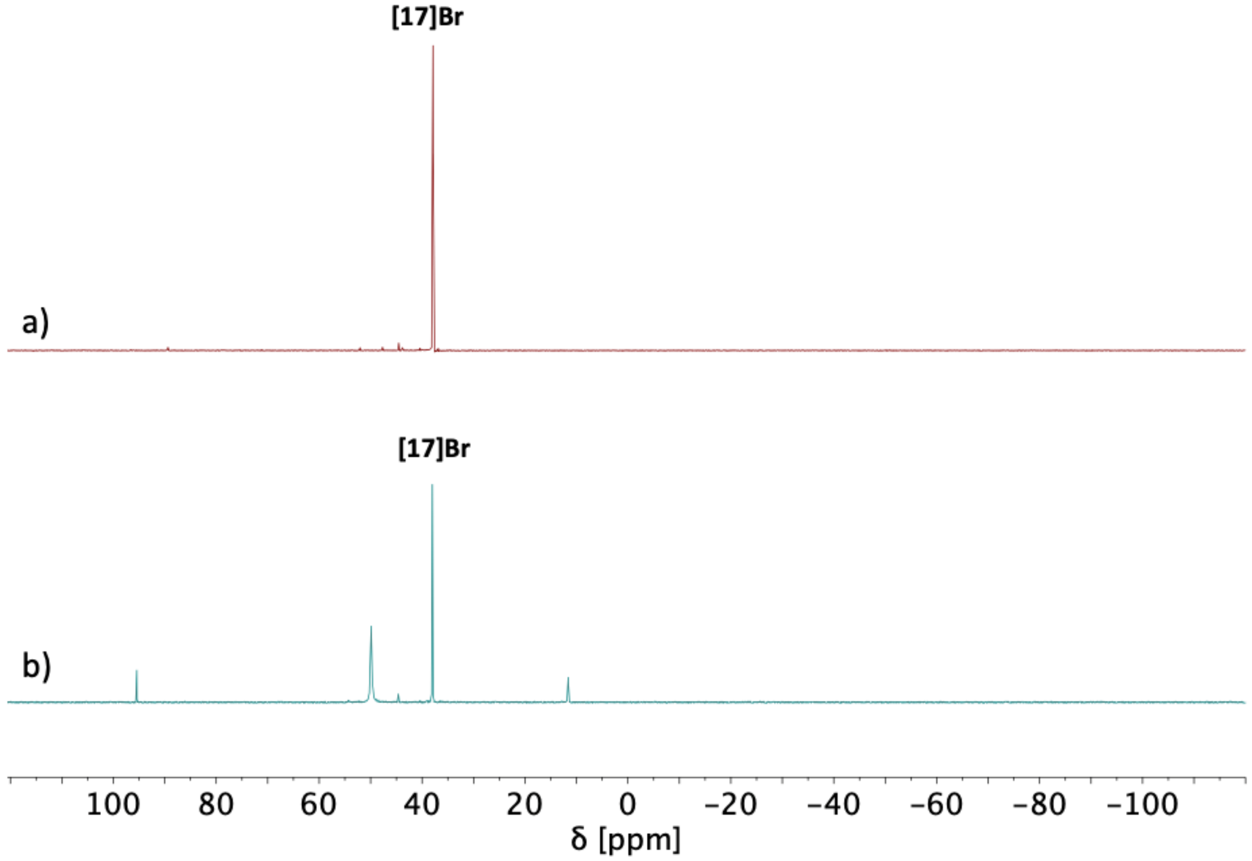

Figure S38 {^{1}H}^{31}P NMR (202 MHz, CD_3CN) spectra of the system after 18 hours at 40 °C from the addition of Ph_2SiH_2 (spectrum a)) and after 10 minutes at 20 °C from the addition of [Me_4N]F (spectrum b)).

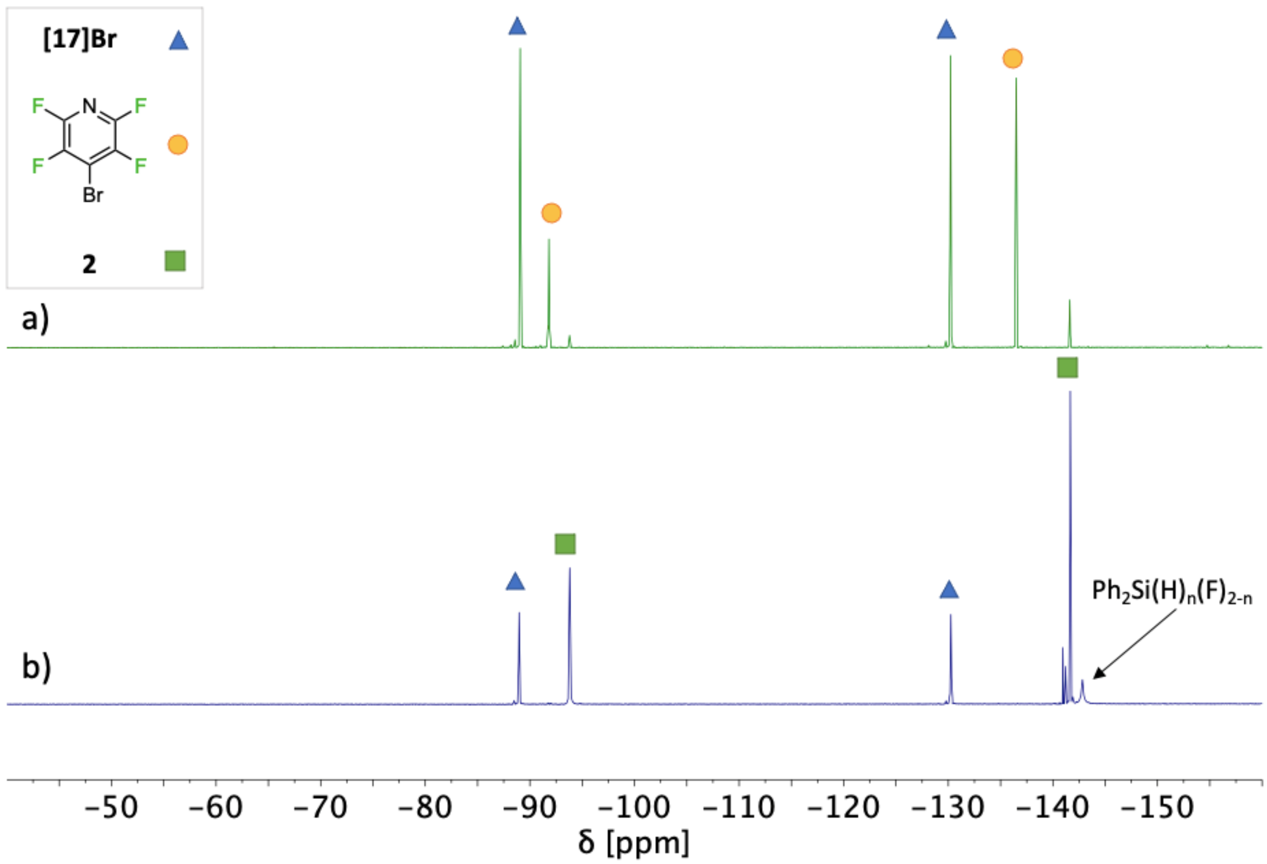

Figure S39 ^{19}F NMR (471 MHz, CD_3CN) spectra of the system after 18 hours at 40 °C from the addition of Ph_2SiH_2 (spectrum a)) and after 10 minutes at 20 °C from the addition of $[Me_4N]F$ (spectrum b)).

2.7. Observation of the phosphonium salt [18][$Ph_2Si(H)_{3-n}F_n$]

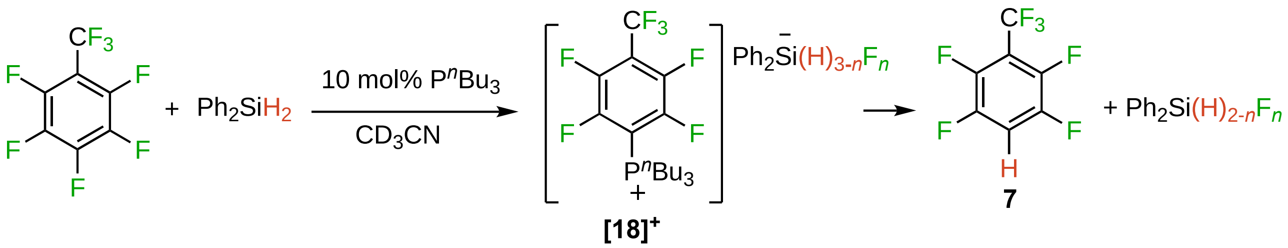

Perfluorotoluene (57 µL, 0.40 mmol, 1.0 equivalent) and Ph_2SiH_2 (74 µL, 0.40 mmol, 1.0 equivalent) were dissolved in 0.4 mL of CD_3CN and placed into a J-Young NMR tube. P^nBu_3 (10 µL, 0.040 mmol, 0.10 equivalent) was added and the progress of reaction was monitored by ^1H, ^{31}P and quantitative ^{19}F NMR with trifluorotoluene (25 µL, 0.20 mmol, 0.50 equivalent) used as an internal standard, as described in section 1.7. After 10 minutes at 20 °C from the start of the reaction an apparent septet (coupling constant of *ca.* 4 Hz) was observed in the ^{31}P NMR spectrum.

[18]$^+$: {^1H}^{31}P NMR (202 MHz, CD_3CN): δ 38.10 ppm (mc, 1P). ^{19}F NMR (471 MHz, CD_3CN): δ –58.0 (t, $^4J_{FF}$ = 18 Hz, 3F), –126.8 (mc, 2F), –137.3 (mc, 2F). ^1H signals of **[18]$^+$** were not identified due to their low intensity.

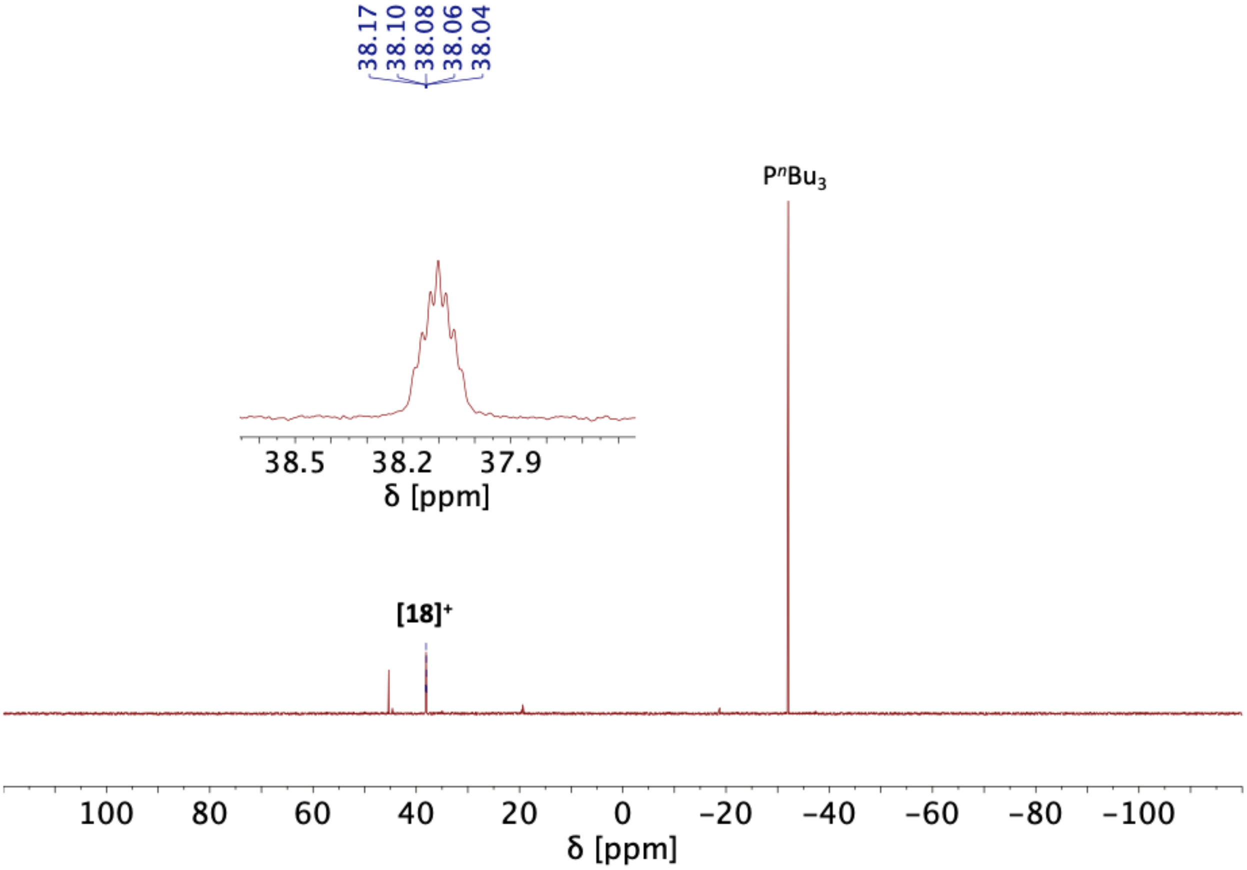

Figure S40 {^1H}^{31}P NMR (202 MHz, CD_3CN) spectrum of the system after 10 minutes at 20 °C.

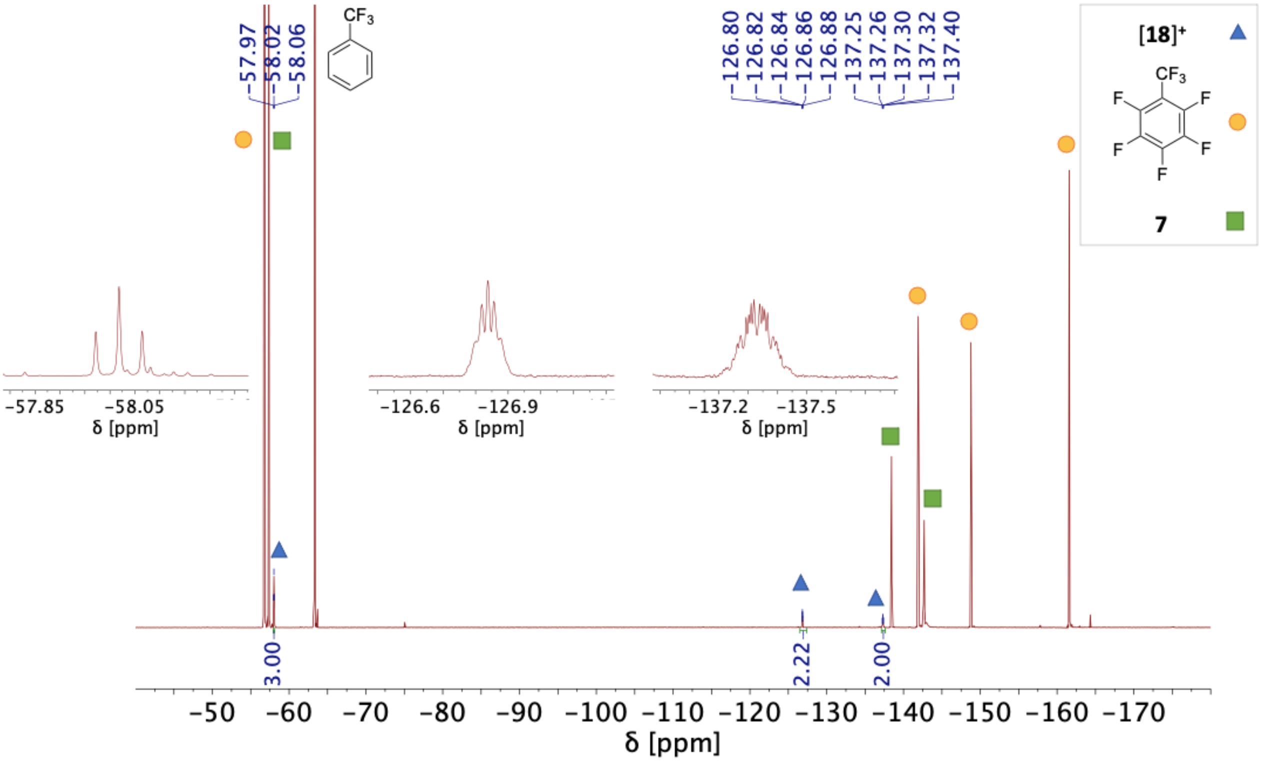

Figure S41 ^{19}F NMR (471 MHz, CD_3CN) spectrum of the system after 10 minutes at 20 °C.

2.8. Characterisation and reactivity of the phosphonium salt [16]Br

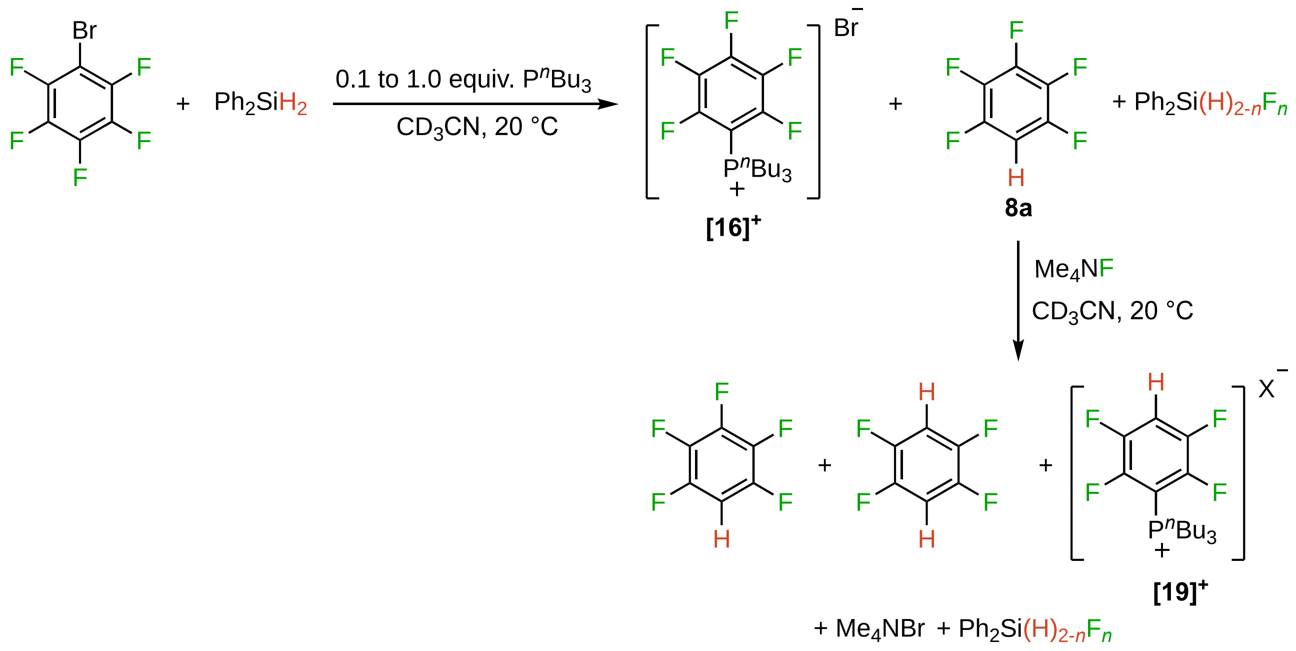

Bromopentafluorobenzene (51 µL, 0.40 mmol, 1.0 equivalent) and Ph_2SiH_2 (74 µL, 0.40 mmol, 1.0 equivalent) were dissolved in 0.4 mL of CD_3CN and placed into a J-Young NMR tube. P^nBu_3 (10 µL, 0.040 mmol, 0.10 equivalent) was added and the reaction mixture was monitored by quantitative ^{19}F NMR with trifluorotoluene (25 µL, 0.20 mmol, 0.50 equivalent) used as an internal standard, as described in section 1.7.

The formation of **[16]Br** (11% conversion) and pentafluorobenzene (3% conversion) was observed. More P^nBu_3 (90 µL, 0.36 mmol, 0.90 equivalent) was added in the mixture and the system was monitored over 18 hours at 20 °C by ^{31}P and ^{19}F NMR spectroscopy. The amount of **[16]Br** and pentafluorobenzene increased. The introduction of anhydrous $[NMe_4]F$ (37 mg, 0.40 mmol, 1.0 equivalent) in the mixture led to an increase in the amount of pentaflurobenzene, the formation of **8a** and the precipitation of a white solid identified as $[NMe_4]Br$. The system was monitored over 18 hours at 20 °C by ^{31}P and ^{19}F NMR spectroscopy.

[16]Br: ^{1}H NMR (500 MHz, CD_3CN): δ 2.93 (td, J = 12.9, 8.6 Hz, 6H), 1.65 (dq, J = 16.6, 8.6 Hz, 6H), 1.59 – 1.49 (m, 6H), 0.95 (t, $^3J_{HH}$ = 7.4 Hz, 9H). {^{1}H}^{31}P NMR (202 MHz, CD_3CN): δ 36.79 – 36.50 (m, 1P). ^{19}F NMR (471 MHz, CD_3CN): δ –128.37 (mc, 2F), –143.48 (mc, 1F), –158.54 (mc, 2F).

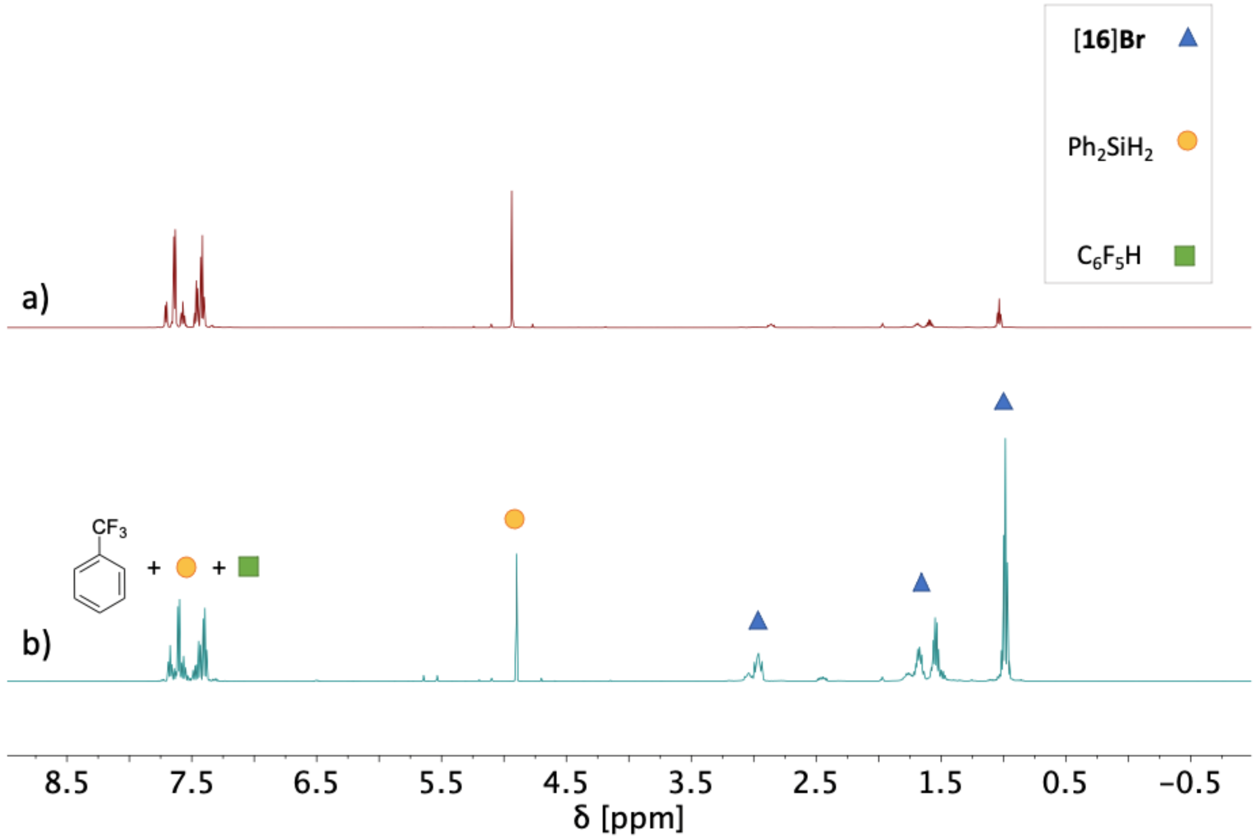

Figure S42 ^{1}H NMR (600 MHz, CD_3CN) spectra of the system after 10 minutes at 20 °C from the addition of 10 mol% of P^{n}Bu$_3$ (spectrum a)) and after 18 hours at 20 °C from the further addition of 0.9 equivalents of P^{n}Bu$_3$ (spectrum b)).

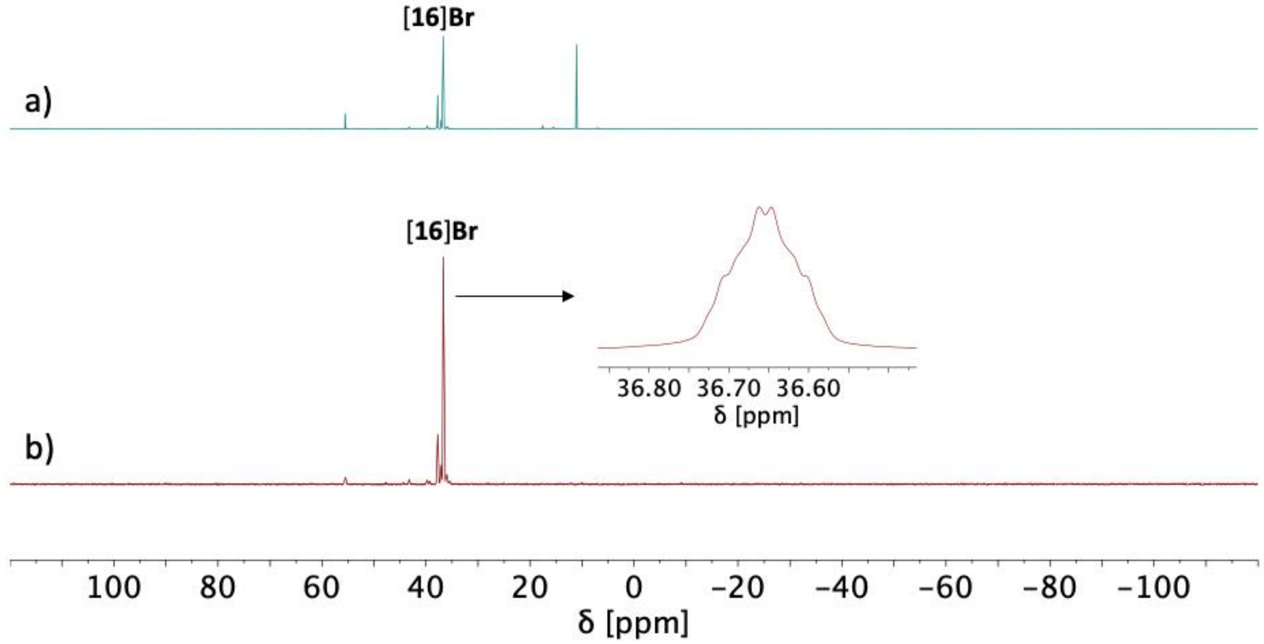

Figure S43 {^{1}H}^{31}P NMR (202 MHz, CD_3CN) spectra of the system after 10 minutes at 20 °C from the addition of 10 mol% of P^{n}Bu$_3$ (spectrum a)) and after 18 hours at 20 °C from the further addition of 0.9 equivalents of P^{n}Bu$_3$ (spectrum b)).

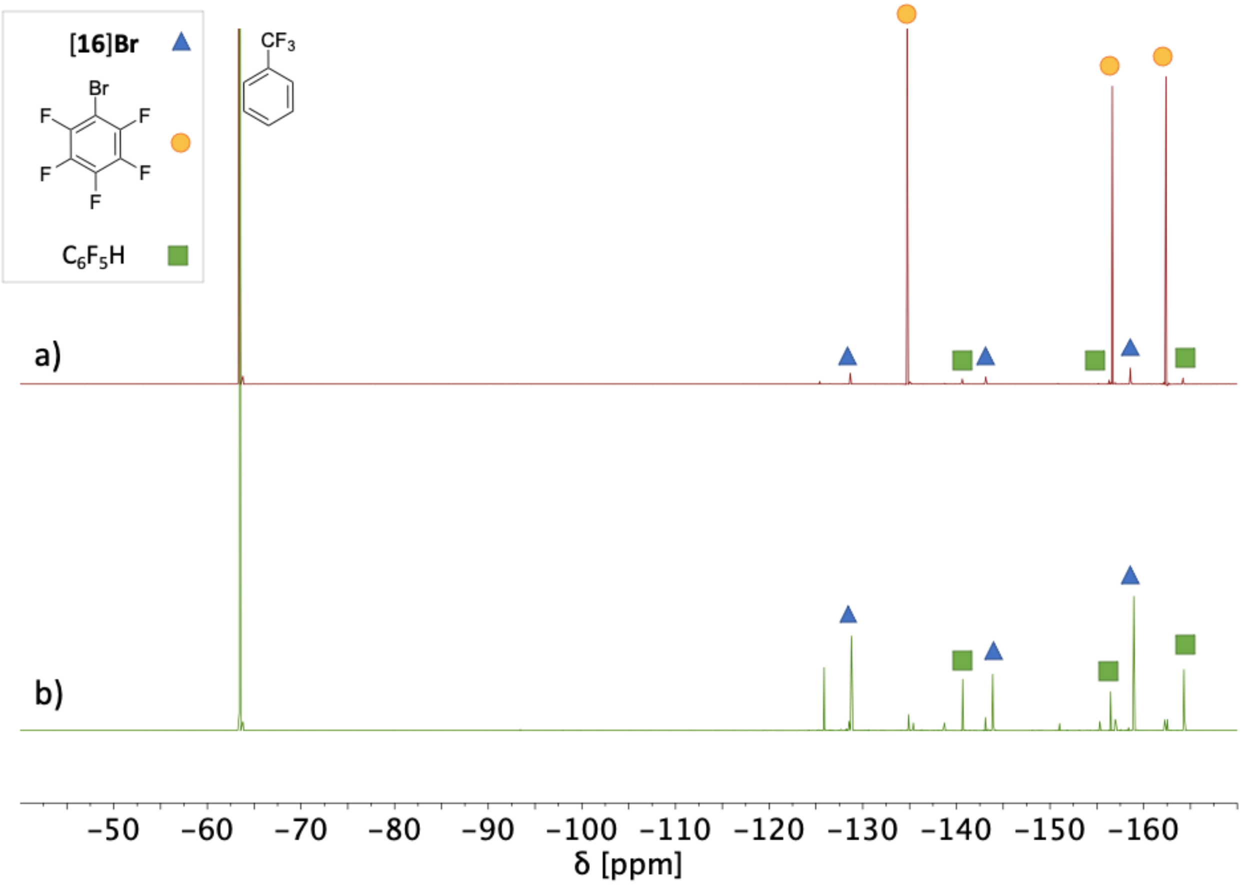

Figure S44 ^{19}F NMR (471 MHz, CD_3CN) spectra of the system after 10 minutes at 20 °C from the addition of 10 mol% of P^nBu_3 (spectrum a)) and after 18 hours at 20 °C from the further addition of 0.9 equivalents of P^nBu_3 (spectrum b)).

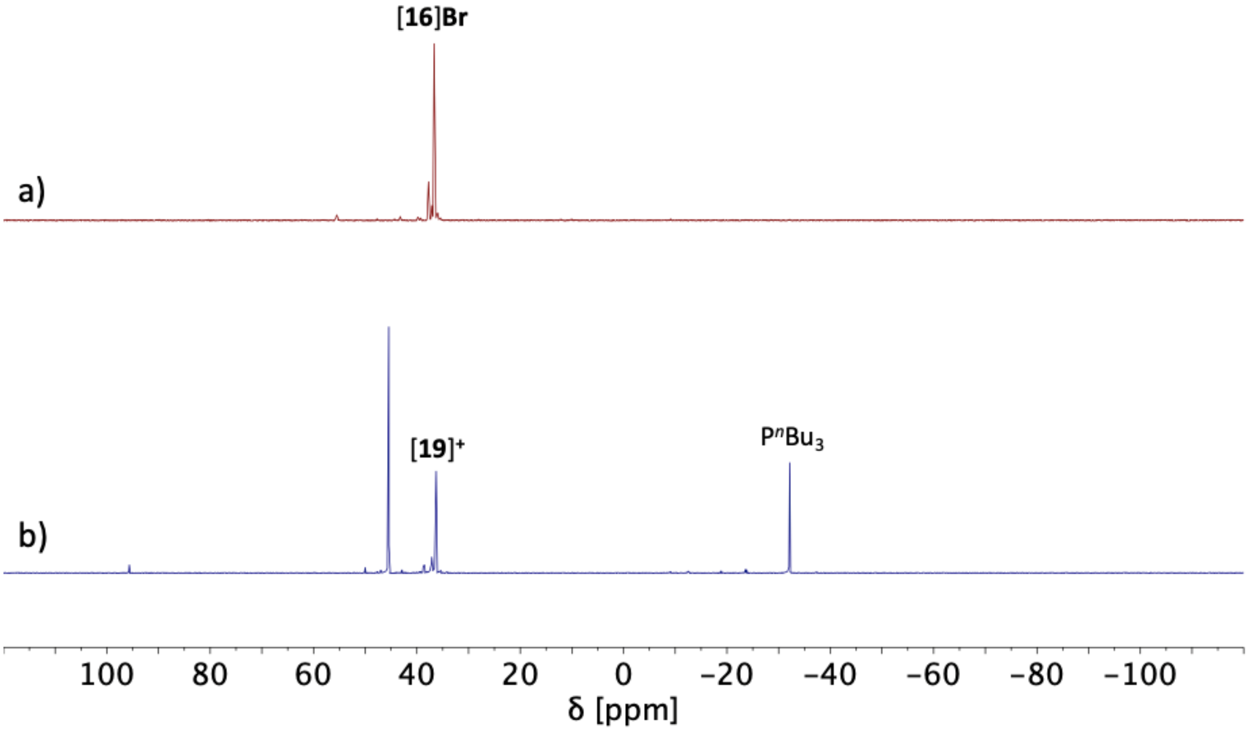

Figure S45 {^{1}H}^{31}P NMR (202 MHz, CD_3CN) spectra of the system after 18 hours at 20 °C from the further addition of 0.9 equivalents of P^nBu_3 (spectrum a)) and after 10 minutes at 20 °C from the addition of $[Me_4N]F$ (spectrum b)).

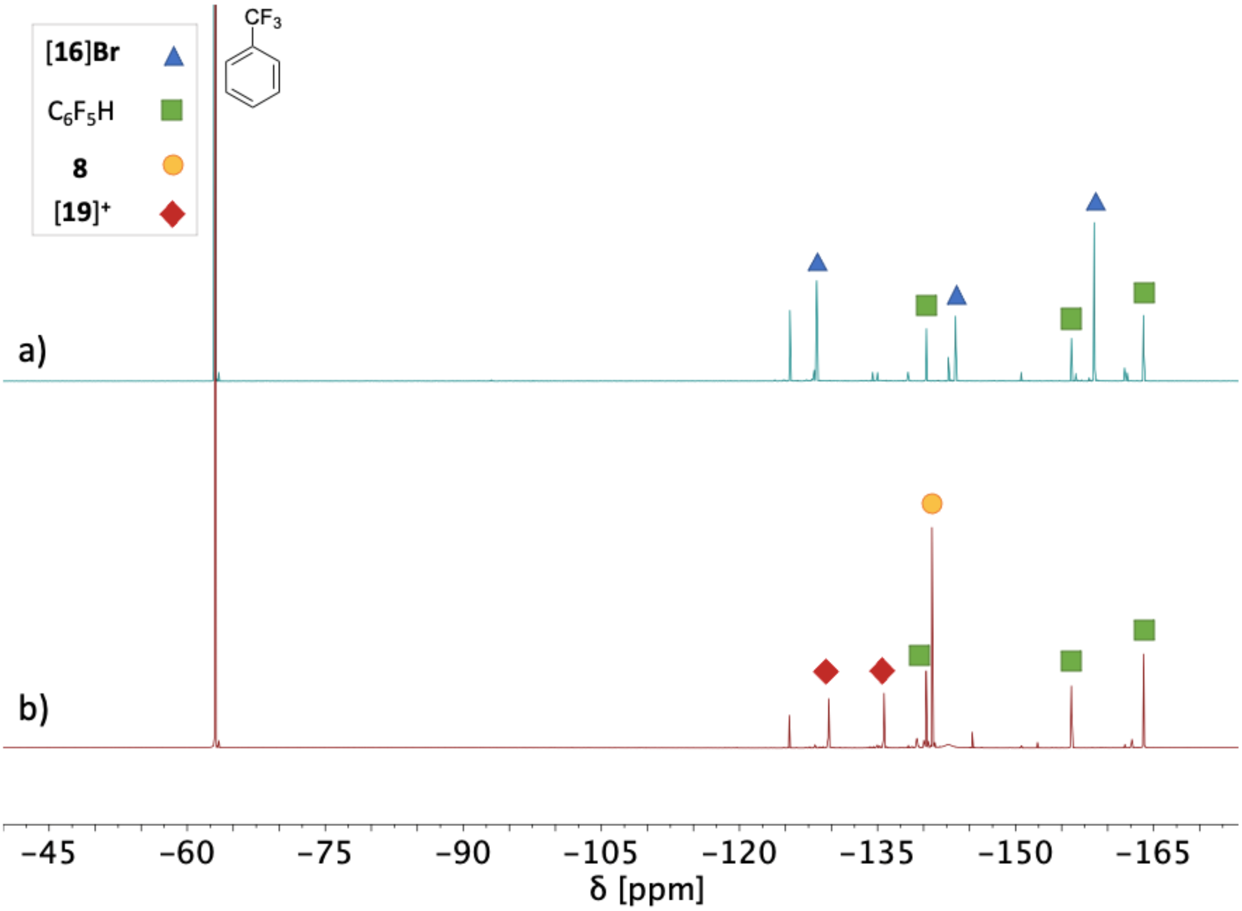

Figure S46 ^{19}F NMR (471 MHz, CD_3CN) spectra of the system after 18 hours at 20 °C from the further addition of 0.9 equivalents of P^nBu_3 (spectrum a)) and after 10 minutes at 20 °C from the addition of $[Me_4N]F$ (spectrum b)).

3. Computational Chemistry

Initial optimisations were performed at the (RI-)BP86/SV(P) level, followed by frequency calculations at the same level. Transition states were located by initially performing a constrained minimisation (by freezing internal coordinates that change most during the reaction) of a structure close to the anticipated transition state. This was followed by a frequency calculation to identify the transition vector to follow during a subsequent transition state optimisation. A final frequency calculation was then performed on the optimised transition-state structure. All minima were confirmed as such by the absence of imaginary frequencies and all transition states were identified by the presence of only one imaginary frequency.

Single-point calculations on the (RI-)BP86/SV(P) optimised geometries were performed using the hybrid PBE0 functional and the flexible def2-TZVPP basis set. The (RI-)PBE0/def2-TZVPP SCF energies were corrected for their zero-point energies, thermal energies and entropies (obtained from the (RI-)BP86/SV(P)-level frequency calculations). No symmetry constraints were applied during optimisations. All calculations were performed using the TURBOMOLE V6.40 package using the resolution of identity (RI) approximation.[12–22] Solvation effects were modelled using the COSMO module of TURBOMOLE.[23] The dielectric constant used was for MeCN (ε = 35.9 at 25 °C).[24]

Both enthalpies and Gibbs energies at 298.15 K are shown on the PES'. Gibbs energy changes are discussed in the main section of the manuscript. The difficulty in assessing entropy changes in solution from gas-phase calculations is acknowledged.[25–27] Single-point DFT-D3 corrections (on the (RI-)BP86/SV(P) geometries) have been applied at the PBE0-D3 level using Grimme's DFT-D3 V3.0 Rev 2 program (with BJ-damping)[28,29] and data presented in the main section of the paper includes this correction. Both DFT-D3 and DFT data are presented below.

Structures were visualised and modified using Facio,[30] Jmol,[31] and gOpenMol.

3.1. Tabulated energies for mechanism presented in scheme 4

Table S1: Energies and corrections at the PBE0/def2-TZVPP//BP86/SV(P) level. As described above, vibrational frequencies and derived corrections were at the BP86/SV(P) level and electronic energies, both in the gas phase and COSMO solvation, in addition to DFT-D3 corrections were at the PBE0/def2-TZVPP level.

	SCF (a.u.)	DFT-D3 corr. (a.u.)	COSMO (MeCN) (a.u.)	ZPE (a.u.)	Chem. Pot. (kJ/mol)	Energy (kJ/mol)	Entropy (kJ/K/mol)	Entropy at 1 mol/dm3 (kJ/K/mol)
Ph_2SiH_2	-753.483828	-0.0272	-753.492017	0.193283	398.96	538.61	0.4767	0.4500
Ph_2SiHF	-852.752370	-0.0276	-852.762224	0.188550	386.01	528.26	0.4854	0.4587
$[Ph_2SiH_2F]^-$	-853.358399	-0.0279	-853.443879	0.194003	401.36	543.07	0.4836	0.4569
**1**	-744.021127	-0.0094	-744.026087	0.047281	34.67	146.81	0.3844	0.3577
**M1**	-460.870945	-0.0087	-460.876044	0.108851	208.40	303.74	0.3281	0.3014
**TS_{12}**	-1204.869012	-0.0257	-1204.889163	0.156088	292.08	453.44	0.5495	0.5228
**M2**	-1204.924023	-0.0264	-1204.934966	0.158689	302.77	459.48	0.5339	0.5072
**M3**	-1204.927511	-0.0265	-1204.939349	0.159287	304.61	460.72	0.5319	0.5052
**M4**	-1104.895087	-0.0247	-1104.978201	0.157190	304.30	451.77	0.5029	0.4762
**M5**	-1105.666078	-0.0259	-1105.676025	0.164088	314.91	472.54	0.5370	0.5103
**M6**	-1105.658139	-0.0258	-1105.665154	0.163477	316.35	470.38	0.5250	0.4983
**TS_{61}**	-1105.624638	-0.0265	-1105.642515	0.160201	307.65	461.22	0.5234	0.4967
**2**	-644.842167	-0.0091	-644.848657	0.055077	58.55	164.65	0.3642	0.3375

Table S2: Relative energies of different states in the gas phase, with COSMO solvation in MeCN and with both COSMO solvation and DFT-D3 corrections.

					Gas Phase 298.15 K			COSMO MeCN 298.15 K			DFT-D3 COSMO MeCN 298.15 K		
	Electronic E gas (kJ/mol)	Electronic E+D gas (kJ/mol)	Electronic E MeCN (kJ/mol)	Electronic E+D MeCN (kJ/mol)	Rel H (kJ/mol)	Rel S (J/K/mol)	Rel G (kJ/mol)	Rel H (kJ/mol)	Rel S (J/K/mol)	Rel G (kJ/mol)	Rel H (kJ/mol)	Rel S (J/K/mol)	Rel G (kJ/mol)
Ph_2SiH_2													
Ph_2SiHF													
$[Ph_2SiH_2F]^-$													
**1**													
**M1**	0	0	0	0	0	0	0	0	0	0	0	0	0
**TS_{12}**	61	41	34	14	61	-163	110	34	-136	75	14	-136	55
**M2**	-84	-106	-86	-108	-77	-179	-24	-80	-152	-34	-102	-152	-56
**M3**	-93	-115	-98	-120	-85	-181	-32	-90	-154	-44	-112	-154	-66
**M4**	321	302	-73	-93	325	-203	385	-70	-176	-18	-89	-176	-37
**M5**	-112	-133	-116	-137	-103	-167	-53	-107	-140	-65	-128	-140	-86
**M6**	-91	-112	-87	-108	-84	-179	-31	-80	-152	-35	-101	-152	-56
**TS_{61}**	-3	-26	-28	-51	-5	-180	49	-30	-154	16	-53	-154	-7
**2**	-235	-236	-244	-244	-228	-12	-224	-236	-12	-233	-237	-12	-233

3.2. 3-component alternative mechanism for Si-H activation (c.f. Piers' mechanism)

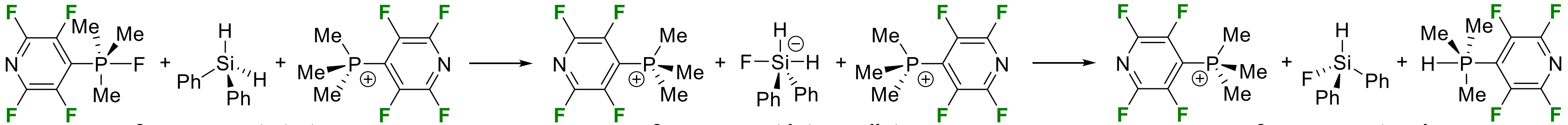

3-component start **3-component intermediate** **3-component end**

Table S3: Energies and corrections at the PBE0/def2-TZVPP//BP86/SV(P) level. As described above, vibrational frequencies and derived corrections were at the BP86/SV(P) level and electronic energies, both in the gas phase and COSMO solvation, in addition to DFT-D3 corrections were at the PBE0/def2-TZVPP level.

	SCF (a.u.)	DFT-D3 corr. (a.u.)	COSMO (MeCN) (a.u.)	ZPE (a.u.)	Chem. Pot. (kJ/mol)	Energy (kJ/mol)	Entropy (kJ/K/mol)	ln q(vib)	HOMO (eV)	LUMO (eV)	Entropy at 1 mol/dm3 (kJ/K/mol)
Start	-3063.326992	-0.096063	-3063.403577	0.511531	1102.95	1467.39	1.2307	58.08	-9.1374	-5.6136	1.2040
TS1	-3063.316750	-0.098043	-3063.390751	0.510491	1105.55	1462.63	1.2060	55.99	-9.2997	-5.4042	1.1793
Intermediate	-3063.327974	-0.096200	-3063.405328	0.510779	1110.60	1463.02	1.1903	54.19	-8.5633	-5.0345	1.1636
TS2	-3063.323394	-0.096075	-3063.399502	0.510378	1106.89	1461.48	1.1976	55.27	-8.6778	-5.0141	1.1709
End	-3063.330657	-0.095090	-3063.409383	0.511033	1095.48	1466.34	1.2522	60.52	-8.7458	-5.6035	1.2255

Table S4: Relative energies of different states in the gas phase, with COSMO solvation in MeCN and with both COSMO solvation and DFT-D3 corrections.

					Gas Phase 298.15 K			COSMO MeCN 298.15 K			DFT-D3 COSMO MeCN 298.15 K		
	Electronic E gas (kJ/mol)	Electronic E+D gas (kJ/mol)	Electronic E MeCN (kJ/mol)	Electronic E+D MeCN (kJ/mol)	Rel H (kJ/mol)	Rel S (J/K/mol)	Rel G (kJ/mol)	Rel H (kJ/mol)	Rel S (J/K/mol)	Rel G (kJ/mol)	Rel H (kJ/mol)	Rel S (J/K/mol)	Rel G (kJ/mol)
Start	0	0	0	0	0	0	0	0	0	0	0	0	0
TS1	27	22	34	28	22	-25	29	29	-25	36	24	-25	31
Intermediate	-3	-3	-5	-5	-7	-40	5	-9	-40	3	-9	-40	3
TS2	9	9	11	11	4	-33	13	5	-33	15	5	-33	15
End	-10	-7	-15	-13	-11	22	-17	-16	22	-23	-14	22	-20

3.3. xyz coordinates

Ph_2SiH_2

25

Si	-2.13544	-1.15576	1.18329
H	-2.66898	-0.67759	2.51312
C	-2.39619	0.15300	-0.15735
C	-0.30609	-1.58561	1.41980
C	-2.77697	2.11772	-2.17239
C	-3.29755	0.82407	-2.34243
C	-3.10879	-0.14682	-1.34272
C	-1.87792	1.46292	-0.00669
C	-2.06627	2.43638	-1.00112
H	-2.92531	2.88119	-2.95443
H	-3.85556	0.56819	-3.25878
H	-3.52727	-1.15780	-1.48939
H	-1.31504	1.73178	0.90462
H	-1.65658	3.45109	-0.86247
C	2.41767	-2.30569	1.77081
C	1.92209	-2.02393	0.48540
C	0.57415	-1.66646	0.31403
C	0.21278	-1.86822	2.70573
C	1.56062	-2.22629	2.88192
H	3.47621	-2.58456	1.90683
H	2.59133	-2.08072	-0.38968
H	0.20371	-1.44160	-0.70154
H	-0.44538	-1.80530	3.59010
H	1.94525	-2.44090	3.89319
H	-2.92247	-2.38111	0.77816

Ph_2SiHF

25

Si	-2.11942	-1.27531	1.04004
H	-2.78336	-1.00456	2.36511
C	-2.37445	0.11332	-0.20137
C	-0.30342	-1.64426	1.34591
C	-2.80078	2.21433	-2.05595
C	-3.44072	0.97602	-2.23453
C	-3.23105	-0.06448	-1.31324
C	-1.73495	1.36606	-0.03903
C	-1.94687	2.40891	-0.95563
H	-2.96661	3.03164	-2.77787
H	-4.10914	0.81872	-3.09774
H	-3.73786	-1.03291	-1.46040
H	-1.05456	1.53459	0.81486
H	-1.44117	3.37867	-0.81293
C	2.42920	-2.23183	1.82053
C	1.99081	-1.92284	0.52036
C	0.63785	-1.62832	0.28735
C	0.15768	-1.95615	2.64748
C	1.51191	-2.24688	2.88539

H	3.49258	-2.45989	2.00465
H	2.70963	-1.90820	-0.31588
H	0.30915	-1.37967	-0.73670
H	-0.55274	-1.97129	3.49287
H	1.85375	-2.48609	3.90633
F	-2.88046	-2.59756	0.40442

$[Ph_2SiH_2F]^-$
26

Si	-1.73148	-0.37400	1.56008
H	-0.93286	0.97100	1.87951
C	-2.36134	0.41207	-0.12408
C	-0.04536	-1.37686	1.61003
C	-3.26532	1.58539	-2.57299
C	-3.60649	0.25903	-2.24935
C	-3.16262	-0.31125	-1.04224
C	-2.03201	1.74137	-0.47683
C	-2.47463	2.32796	-1.67759
H	-3.61372	2.03750	-3.51953
H	-4.22689	-0.33450	-2.94661
H	-3.42941	-1.34613	-0.77530
H	-1.40397	2.32258	0.22390
H	-2.19946	3.37122	-1.92038
C	2.42862	-2.81312	1.69920
C	2.40279	-1.42546	1.92795
C	1.18128	-0.72738	1.88114
C	0.00987	-2.77480	1.38432
C	1.22315	-3.48580	1.42560
H	3.38398	-3.36813	1.73319
H	3.34333	-0.88493	2.14291
H	1.16073	0.36406	2.05798
H	-0.93995	-3.29104	1.17276
H	1.23184	-4.57665	1.24360
F	-2.61600	-1.87221	1.19538
H	-2.57307	-0.22693	2.84036

1
11

C	0.22445	-1.15121	0.52387
F	0.44915	-2.35155	1.04738
N	-0.25113	1.35707	-0.56754
C	-0.47824	0.27002	-1.28098
C	-0.25904	-1.03114	-0.79315
C	0.46625	0.00980	1.28308
C	0.20319	1.24685	0.66698
F	-0.93935	0.42043	-2.52370
F	-0.49699	-2.11758	-1.52987
F	0.92417	-0.08138	2.53278
F	0.42055	2.36770	1.35715

M1
13

C	-0.72666	0.17095	-1.50065
P	-0.99328	0.30647	0.34773
H	0.21015	-0.37192	-1.76608
H	-0.68648	1.19006	-1.94485
H	-1.58928	-0.35964	-1.96092
C	0.68237	0.97837	0.84469
C	-0.74674	-1.48872	0.81766
H	0.76371	0.98507	1.95407
H	0.77736	2.03070	0.49672
H	1.52950	0.38607	0.42732
H	-0.72387	-1.58176	1.92600
H	0.19340	-1.92014	0.40277
H	-1.60818	-2.08951	0.45054

TS_{12}
24

C	0.87757	-2.62738	-1.81662
C	0.26183	-3.17652	1.01024
C	-0.62973	-0.16173	-0.13801
C	2.36106	-1.21974	0.31060
F	-1.72753	-1.00109	-0.52578
P	0.70338	-1.83746	-0.16710
N	0.02060	2.58667	0.50142
C	0.10176	2.11751	-0.73652
C	-0.22717	0.81759	-1.11397
C	-0.72229	0.39959	1.18471
C	-0.37119	1.72508	1.43057
F	0.51896	2.97094	-1.68876
F	-0.06548	0.39148	-2.39785
F	-1.05525	-0.44282	2.20227
F	-0.44607	2.17900	2.69436
H	1.21268	-1.86349	-2.54787
H	-0.12184	-2.99183	-2.13458
H	1.59684	-3.47666	-1.79616
H	0.20138	-2.75038	2.03281
H	0.99955	-4.00988	0.98832
H	-0.74345	-3.56076	0.73710
H	2.30433	-0.77436	1.32744
H	2.66714	-0.42116	-0.39958
H	3.12494	-2.02741	0.30563

M2
24

C	-0.04471	-1.54375	-1.09175
C	-0.26543	-0.23352	1.75930
C	0.02591	1.70712	-0.78296
C	1.96285	0.00153	0.05237
F	-1.69831	0.12976	-0.24687

P	0.05767	-0.02155	-0.04644
N	-0.03658	4.31625	-1.85330
C	-0.35998	3.27487	-2.59688
C	-0.36137	1.95406	-2.11139
C	0.36669	2.83389	-0.01758
C	0.31323	4.11426	-0.59830
F	-0.70497	3.50029	-3.86825
F	-0.70698	0.95492	-2.93959
F	0.74544	2.70964	1.27115
F	0.63287	5.17929	0.14293
H	-0.15340	-1.22694	-2.14854
H	-0.95897	-2.10289	-0.81293
H	0.86217	-2.17365	-0.98846
H	-0.73729	0.69457	2.13873
H	0.65719	-0.44733	2.33548
H	-1.00450	-1.05184	1.87661
H	2.32218	0.85052	0.67384
H	2.40768	0.10042	-0.96357
H	2.33761	-0.94490	0.50440

M3

24

C	-0.11912	-1.24603	-1.31472
C	0.03649	-0.35629	1.87288
F	1.67469	-0.28249	0.04752
C	0.20185	1.75816	-0.41885
C	-2.08117	0.09189	0.11228
P	-0.05378	0.00009	0.05675
N	-4.92085	-0.15449	0.27764
C	-4.35400	0.96927	-0.11332
C	-2.95622	1.14100	-0.21441
C	-2.73450	-1.08850	0.51442
C	-4.13477	-1.16988	0.58741
F	-5.15454	1.99380	-0.43041
F	-2.51976	2.34806	-0.63579
F	-2.00761	-2.18477	0.84076
F	-4.71283	-2.31096	0.98172
H	0.66425	-0.96180	-2.04565
H	-1.11171	-1.31356	-1.80177
H	0.16086	-2.23262	-0.89184
H	-0.91273	-0.16498	2.41028
H	0.85405	0.27359	2.27844
H	0.33661	-1.41598	2.00112
H	-0.29876	2.43030	0.30540
H	-0.23598	1.94982	-1.41884
H	1.29154	1.94538	-0.43602

$[M4]^+$

23

C	-0.33833	-1.28278	-2.67444
C	-1.18497	-2.48407	-0.10772

C	0.02822	0.22113	-0.08018
C	1.71471	-2.12862	-0.71870
P	0.05171	-1.41099	-0.90383
N	0.03781	2.64653	1.31522
C	-0.22892	2.63308	0.02563
C	-0.25177	1.44070	-0.73452
C	0.31240	0.27761	1.30335
C	0.30172	1.52516	1.95740
F	-0.48402	3.77808	-0.58259
F	-0.53240	1.48970	-2.03875
F	0.59020	-0.84726	1.98174
F	0.56435	1.57807	3.25123
H	0.40559	-0.64697	-3.19679
H	-1.35253	-0.85904	-2.82349
H	-0.30389	-2.31119	-3.09714
H	-0.96279	-2.58129	0.97507
H	-1.15280	-3.48843	-0.58385
H	-2.19801	-2.04732	-0.24059
H	1.96823	-2.22733	0.35711
H	2.46043	-1.47230	-1.21648
H	1.73206	-3.13247	-1.19668

M5
24

C	-0.09198	-1.24529	-1.32713
C	0.08214	-0.35769	1.87204
H	1.55197	-0.24021	0.03097
C	0.23799	1.76721	-0.41005
C	-2.10241	0.09304	0.11372
P	0.07498	-0.01120	0.05016
N	-4.94073	-0.17060	0.28248
C	-4.38020	0.95577	-0.11509
C	-2.98353	1.12994	-0.21674
C	-2.74539	-1.08443	0.52002
C	-4.14550	-1.17823	0.59622
F	-5.19222	1.97391	-0.43606
F	-2.54644	2.34131	-0.64584
F	-2.01138	-2.18253	0.85396
F	-4.71816	-2.32264	0.99847
H	0.73811	-1.06395	-2.04334
H	-1.07595	-1.19073	-1.83540
H	0.03917	-2.25874	-0.89119
H	-0.86503	-0.06520	2.36882
H	0.94354	0.19495	2.30593
H	0.25633	-1.44418	2.01893
H	-0.33537	2.39722	0.29722
H	-0.18841	1.93075	-1.42024
H	1.31050	2.05054	-0.40289

M6
24

C	-0.06232	-1.61127	-1.05202
C	-0.27460	-0.29629	1.76328
C	0.04434	1.76108	-0.79523
C	1.93860	0.01075	0.03830
H	-1.48767	0.11315	-0.25069
P	0.00313	-0.03138	-0.05707
N	-0.06648	4.36543	-1.86897
C	-0.35480	3.31721	-2.61809
C	-0.32147	1.99847	-2.12801
C	0.35001	2.89231	-0.02661
C	0.27438	4.17199	-0.60917
F	-0.68526	3.53581	-3.89572
F	-0.63933	0.97764	-2.95012
F	0.72061	2.77933	1.26778
F	0.56400	5.24731	0.13167
H	-0.13712	-1.33248	-2.12390
H	-0.98707	-2.16140	-0.77552
H	0.83764	-2.24375	-0.90397
H	-0.47595	0.69116	2.22924
H	0.60873	-0.74855	2.26010
H	-1.17341	-0.93514	1.89405
H	2.27335	0.86584	0.66603
H	2.36581	0.13804	-0.98236
H	2.34389	-0.93024	0.47499

TS_{61}
24

C	-0.28167	-2.94270	-1.54688
C	-0.88049	-2.85079	1.41185
C	-0.23144	-0.27684	-0.05966
C	1.78854	-2.43269	0.40075
H	-1.64144	-1.09639	-0.41019
P	-0.00314	-2.04456	0.02406
N	-0.15212	2.59771	-0.08013
C	0.19247	1.88551	-1.14439
C	0.25625	0.49486	-1.17405
C	-0.40191	0.52865	1.12330
C	-0.43052	1.91602	1.02449
F	0.55211	2.56610	-2.24616
F	0.66951	-0.15541	-2.30031
F	-0.68060	-0.08831	2.30487
F	-0.71229	2.62910	2.12842
H	0.18872	-2.37286	-2.37275
H	-1.36594	-3.04823	-1.74347
H	0.19933	-3.94225	-1.46179
H	-0.41570	-2.55543	2.37350
H	-0.77813	-3.94915	1.27288
H	-1.95099	-2.56925	1.40493
H	2.08552	-1.91036	1.33600

H	2.42278	-2.05100	-0.42864
H	1.96858	-3.52292	0.52261

2

11

C	0.22952	-1.18022	0.53641
H	0.41798	-2.17424	0.96915
N	-0.24553	1.33702	-0.55936
C	-0.47384	0.25114	-1.27331
C	-0.25261	-1.04756	-0.77157
C	0.46539	-0.01834	1.28181
C	0.20731	1.22793	0.67536
F	-0.93268	0.40223	-2.51812
F	-0.50078	-2.12300	-1.52736
F	0.92290	-0.08607	2.53718
F	0.42533	2.35010	1.36581

3-component start

72

C	-0.03967	3.99683	-2.43836
C	-1.16436	2.48302	-0.16343
C	-1.22301	5.44992	-0.07550
C	1.36529	4.01359	0.15588
P	-0.26818	3.98863	-0.63711
N	-2.66537	7.63284	0.92992
C	-2.47632	7.51041	-0.36784
C	-1.75878	6.43306	-0.93550
C	-1.44963	5.61413	1.30931
C	-2.17987	6.73105	1.76053
F	-2.97921	8.43369	-1.17198
F	-1.60535	6.37040	-2.26139
F	-0.97430	4.71506	2.18336
F	-2.38876	6.88521	3.05890
H	0.50926	4.90471	-2.76161
H	-1.01719	3.95152	-2.95984
H	0.55999	3.08980	-2.68586
H	-1.24014	2.41184	0.94275
H	-0.58332	1.60975	-0.53893
H	-2.17675	2.48894	-0.62061
H	1.25619	3.88265	1.25307
H	1.88134	4.97064	-0.07139
H	1.95157	3.16018	-0.25644
Si	2.65242	-0.23308	1.11709
C	2.36110	0.25075	-0.70208
C	1.38662	0.56589	2.28313
C	1.98167	0.95953	-3.43207
C	1.06083	0.11384	-2.78292
C	1.24699	-0.23950	-1.43291
C	3.27601	1.10127	-1.37190
C	3.09189	1.45328	-2.72317
H	1.85018	1.20855	-4.49870

H	0.20413	-0.29719	-3.34412
H	0.54064	-0.93913	-0.94727
H	4.16956	1.47416	-0.84056
H	3.83316	2.09305	-3.23119
C	-0.48275	1.79275	4.03856
C	-0.87827	0.71940	3.21878
C	0.04563	0.10936	2.34956
C	1.76836	1.64475	3.11939
C	0.84478	2.25427	3.98889
H	-1.20393	2.25713	4.73160
H	-1.91267	0.33908	3.27355
H	-0.27366	-0.74302	1.72190
H	2.81315	2.00288	3.11321
H	1.16812	3.07851	4.64666
H	4.01476	0.29035	1.49422
H	2.62753	-1.73012	1.25225
C	-2.33690	-3.56792	-0.35688
C	0.85043	-4.33085	-0.68831
F	-0.29427	-2.38385	0.35787
C	-0.51778	-4.29721	2.09958
C	-1.21773	-6.08963	-0.04706
P	-0.71975	-4.20855	0.27790
N	-1.95598	-8.70897	-0.87269
C	-1.78143	-8.42434	0.40155
C	-1.41809	-7.13982	0.86626
C	-1.42220	-6.44290	-1.39596
C	-1.78460	-7.74924	-1.76280
F	-1.96022	-9.40113	1.29136
F	-1.27945	-6.98016	2.19701
F	-1.27198	-5.50810	-2.36505
F	-1.96738	-8.04557	-3.05073
H	-2.68388	-2.78199	0.34217
H	-3.09688	-4.36834	-0.45389
H	-2.16335	-3.09821	-1.34581
H	1.14717	-5.38310	-0.86815
H	1.63305	-3.79399	-0.11682
H	0.71616	-3.81249	-1.65868
H	0.17903	-5.11719	2.36173
H	-1.49481	-4.51786	2.57503
H	-0.12587	-3.32905	2.45999

3-component TS1

72

C	0.73972	3.48712	-1.73214
C	-1.01596	3.71189	0.64234
C	0.16200	6.17641	-0.49199
C	1.91383	4.22269	0.87850
P	0.45184	4.38843	-0.18752
N	-0.29968	8.92555	-0.80033
C	-0.07938	8.17133	-1.85723
C	0.15921	6.78105	-1.76720
C	-0.07674	7.01868	0.61699

C	-0.30359	8.39243	0.40606
F	-0.08224	8.73992	-3.05321
F	0.37522	6.07827	-2.88259
F	-0.08711	6.51397	1.86161
F	-0.52825	9.17915	1.44739
H	1.64939	3.86570	-2.24096
H	-0.13263	3.57959	-2.40973
H	0.87360	2.41366	-1.46123
H	-1.23694	4.29554	1.55901
H	-0.80690	2.65427	0.92419
H	-1.88314	3.75284	-0.05021
H	1.75234	4.75405	1.83851
H	2.80458	4.63771	0.36083
H	2.05116	3.12965	1.06105
Si	0.69234	-0.19510	0.72304
C	0.11815	-0.07633	-1.09023
C	-0.52840	0.16272	2.14544
C	-0.64601	0.20463	-3.82453
C	-1.60625	0.31950	-2.80312
C	-1.22683	0.17596	-1.45490
C	1.06937	-0.18141	-2.13588
C	0.69526	-0.04954	-3.48642
H	-0.94325	0.30474	-4.88154
H	-2.66244	0.51002	-3.05948
H	-2.00207	0.25433	-0.67245
H	2.13167	-0.37009	-1.89545
H	1.45481	-0.14970	-4.28011
C	-2.26486	0.81693	4.30994
C	-2.70274	-0.03562	3.28156
C	-1.83839	-0.36982	2.22426
C	-0.11416	1.02826	3.18886
C	-0.96126	1.34128	4.26682
H	-2.93624	1.06622	5.14834
H	-3.72237	-0.45526	3.31151
H	-2.17717	-1.07204	1.44756
H	0.90442	1.45634	3.16779
H	-0.60207	1.99523	5.07940
H	1.39779	1.20794	0.76717
H	1.93378	-1.00710	0.98909
C	-1.74004	-3.66578	-0.66877
C	1.45919	-3.77851	-0.40971
F	-0.19909	-2.13988	0.62128
C	-0.37285	-4.37070	2.03025
C	-0.23197	-6.03100	-0.35271
P	-0.21708	-4.20018	0.21529
N	-0.21986	-8.62143	-1.49712
C	-0.31251	-8.46267	-0.19310
C	-0.32247	-7.19509	0.43317
C	-0.13304	-6.25005	-1.74282
C	-0.13132	-7.55345	-2.26749
F	-0.40033	-9.55078	0.56773
F	-0.41929	-7.15698	1.77220
F	-0.03826	-5.19543	-2.58003

F	-0.03713	-7.73063	-3.58356
H	-2.39322	-3.15147	0.06257
H	-2.27027	-4.53038	-1.11553
H	-1.46104	-2.93520	-1.45305
H	2.01835	-4.68963	-0.70342
H	1.99331	-3.22994	0.39010
H	1.34929	-3.10050	-1.27957
H	0.49296	-4.94004	2.42430
H	-1.29815	-4.92986	2.27402
H	-0.40213	-3.35531	2.46348

3-component intermediate
72

C	-0.19692	3.90900	-1.73470
C	-0.99242	3.83071	1.10194
C	0.45228	6.20319	0.10783
C	1.89569	3.63031	0.34466
P	0.29587	4.37717	-0.05454
N	0.74905	8.96626	0.50997
C	0.43888	8.50120	-0.68272
C	0.27618	7.12292	-0.94787
C	0.78850	6.73991	1.37079
C	0.92270	8.13357	1.51811
F	0.27221	9.36476	-1.67469
F	-0.04038	6.72713	-2.18489
F	0.98097	5.92921	2.42380
F	1.23359	8.63247	2.70634
H	0.56928	4.22107	-2.47161
H	-1.17374	4.36429	-1.99529
H	-0.27935	2.79708	-1.74946
H	-0.72021	4.10808	2.13991
H	-1.07315	2.72030	1.04385
H	-1.96021	4.29944	0.82366
H	2.19952	3.92693	1.36960
H	2.65386	3.97952	-0.38865
H	1.81473	2.50409	0.29339
Si	0.78925	-0.35916	0.15964
C	0.43710	0.23440	-1.64422
C	-0.17475	0.31116	1.69392
C	0.03381	1.12515	-4.32840
C	-1.05145	0.65983	-3.56408
C	-0.84465	0.20143	-2.24873
C	1.50888	0.72446	-2.43102
C	1.32100	1.14477	-3.76052
H	-0.12152	1.46208	-5.36706
H	-2.06328	0.63532	-4.00404
H	-1.69912	-0.20179	-1.68040
H	2.52148	0.76669	-1.98990
H	2.18209	1.48963	-4.35848
C	-1.50973	1.35181	3.99595
C	-2.24585	0.74721	2.96124
C	-1.58217	0.21716	1.83820

C	0.53987	0.93605	2.74569
C	-0.10901	1.43335	3.89079
H	-2.02502	1.74733	4.88740
H	-3.34406	0.67105	3.03973
H	-2.16851	-0.29289	1.05652
H	1.63895	1.02224	2.66808
H	0.47966	1.88504	4.70776
H	1.92692	0.73906	0.34152
H	1.80454	-1.48539	0.36099
C	-1.13104	-4.17907	-1.55034
C	1.25139	-4.47074	0.21201
F	-0.49924	-1.68119	-0.04675
C	-1.43791	-4.13328	1.37745
C	-0.67870	-6.69812	-0.04048
P	-0.50711	-4.86659	0.00850
N	-0.84305	-9.49343	-0.23542
C	-1.46084	-8.86149	0.74131
C	-1.41484	-7.45740	0.89306
C	-0.02877	-7.41110	-1.07161
C	-0.14426	-8.81332	-1.12366
F	-2.15391	-9.57532	1.61798
F	-2.06577	-6.88648	1.91145
F	0.69586	-6.75888	-1.99505
F	0.46695	-9.47894	-2.09393
H	-1.00500	-3.07552	-1.42876
H	-2.20240	-4.44291	-1.67684
H	-0.54096	-4.55877	-2.40867
H	1.62283	-4.89339	1.16952
H	1.32849	-3.35605	0.23082
H	1.83829	-4.88128	-0.63485
H	-1.04710	-4.47781	2.35599
H	-2.51743	-4.37253	1.29620
H	-1.26281	-3.03801	1.23605

3-component TS2

72

C	0.23160	4.32416	-1.66814
C	-1.18180	3.90563	1.11383
C	0.31444	6.26561	0.47387
C	1.83341	3.72487	0.81368
P	0.28878	4.42673	0.16022
N	0.14842	9.04620	0.86816
C	-0.78009	8.42353	0.16792
C	-0.74406	7.03443	-0.05467
C	1.28268	6.96280	1.22351
C	1.14780	8.36058	1.38361
F	-1.77785	9.13765	-0.33722
F	-1.71729	6.44116	-0.76911
F	2.32737	6.34405	1.78792
F	2.06581	9.01297	2.08549
H	0.70044	5.23709	-2.09619
H	-0.82045	4.25245	-2.00788

H	0.76853	3.41302	-1.99634
H	-1.17521	4.44835	2.08429
H	-1.16305	2.80801	1.27370
H	-2.09363	4.18392	0.54589
H	1.86811	3.84014	1.91558
H	2.70343	4.24377	0.36223
H	1.84486	2.64790	0.53752
Si	0.28540	0.42132	-0.46510
C	-0.52513	0.25045	-2.19425
C	-0.75214	0.15617	1.12459
C	-1.73542	0.04627	-4.77005
C	-2.24599	0.94557	-3.81788
C	-1.63265	1.05468	-2.55738
C	-0.04085	-0.65895	-3.16683
C	-0.62409	-0.74986	-4.44326
H	-2.20062	-0.03057	-5.76687
H	-3.12051	1.57143	-4.06402
H	-2.02844	1.78686	-1.83068
H	0.82095	-1.30029	-2.92082
H	-0.20997	-1.44857	-5.19012
C	-2.27694	-0.17106	3.51734
C	-2.91701	-0.13984	2.26517
C	-2.16307	0.02718	1.08864
C	-0.12987	0.11540	2.39823
C	-0.87842	-0.03975	3.58056
H	-2.86660	-0.29466	4.44093
H	-4.01399	-0.24266	2.20477
H	-2.69082	0.05386	0.11816
H	0.96948	0.20371	2.47485
H	-0.36644	-0.06139	4.55797
H	-0.07325	2.01226	-0.43302
H	1.75948	0.76545	-0.26952
C	-0.96543	-3.79632	-0.90593
C	1.98707	-4.09283	-0.67655
F	0.78547	-1.34661	-0.46546
C	0.36379	-3.50545	1.71022
C	0.19626	-6.21292	0.37076
P	0.39183	-4.39914	0.13588
N	-0.09760	-8.99672	0.57274
C	-0.10046	-8.25148	1.65879
C	0.04298	-6.84615	1.62194
C	0.19559	-7.04522	-0.77004
C	0.04394	-8.43612	-0.61279
F	-0.24408	-8.84719	2.83452
F	0.03014	-6.15538	2.76619
F	0.33899	-6.51573	-1.99592
F	0.04275	-9.21400	-1.68623
H	-0.74211	-2.71312	-1.04808
H	-1.93464	-3.93344	-0.38192
H	-0.97664	-4.32671	-1.87931
H	2.81619	-4.44776	-0.02862
H	2.02789	-2.98554	-0.80390
H	2.02676	-4.60545	-1.65905

H	1.19851	-3.82814	2.36463
H	-0.60319	-3.64657	2.23323
H	0.48385	-2.43785	1.41114

3-component end
72

C	1.36516	4.14510	-1.75554
C	-1.51733	4.24399	-0.31261
C	0.38337	6.13181	0.13952
C	1.34203	3.71571	1.28366
P	0.33470	4.08087	-0.22853
N	0.22583	8.88227	0.86531
C	0.62909	8.51857	-0.33623
C	0.71088	7.17013	-0.74641
C	-0.06414	6.57150	1.39657
C	-0.11957	7.93838	1.72081
F	0.96589	9.48124	-1.19912
F	1.11459	6.93285	-2.01600
F	-0.46896	5.67365	2.33548
F	-0.54040	8.31397	2.93263
H	2.18402	4.88134	-1.64304
H	0.73763	4.45619	-2.61513
H	1.76833	3.13130	-1.95309
H	-1.88279	4.51739	0.69949
H	-1.98705	3.29051	-0.63022
H	-1.77747	5.06887	-1.00905
H	0.66712	3.38015	2.09701
H	1.91422	4.60114	1.62765
H	2.03182	2.88490	1.02426
Si	0.19354	0.20654	-0.61650
C	0.25160	0.61273	-2.44960
C	-1.44954	0.36468	0.28279
C	0.42353	1.14570	-5.23282
C	-0.68517	1.59639	-4.49493
C	-0.76610	1.33636	-3.11653
C	1.35769	0.16021	-3.21247
C	1.44665	0.42802	-4.58902
H	0.49145	1.35640	-6.31277
H	-1.49031	2.15842	-4.99634
H	-1.63679	1.71081	-2.55225
H	2.16949	-0.41011	-2.72758
H	2.32016	0.07667	-5.16288
C	-3.90703	0.48096	1.70178
C	-3.89177	0.09872	0.34893
C	-2.67426	0.03730	-0.35215
C	-1.48653	0.73887	1.64893
C	-2.70090	0.79721	2.35281
H	-4.86186	0.53580	2.25016
H	-4.83573	-0.14654	-0.16578
H	-2.68482	-0.26616	-1.41371
H	-0.55009	0.99776	2.17360
H	-2.70849	1.09684	3.41389

H	0.16382	2.53810	-0.40125
H	1.38672	0.58945	0.20789
C	-0.20295	-4.41161	-1.78162
C	1.60762	-4.17306	0.56718
F	0.42637	-1.50099	-0.64571
C	-1.29961	-3.74797	0.87760
C	-0.20198	-6.50878	0.31949
P	-0.03491	-4.70975	0.00348
N	-0.32991	-9.28063	0.71353
C	-1.19560	-8.48982	1.31334
C	-1.18285	-7.08518	1.15460
C	0.70919	-7.38969	-0.30487
C	0.59983	-8.77458	-0.07329
F	-2.11403	-9.03312	2.09662
F	-2.09058	-6.34144	1.79317
F	1.67058	-6.90933	-1.11037
F	1.45348	-9.59865	-0.65985
H	-0.05264	-3.31963	-1.93300
H	-1.21816	-4.71006	-2.11909
H	0.56283	-4.98656	-2.34120
H	1.69996	-4.33028	1.66278
H	1.68149	-3.08976	0.32535
H	2.39945	-4.74166	0.03789
H	-1.20843	-3.87910	1.97483
H	-2.31714	-4.04368	0.55144
H	-1.10429	-2.68764	0.60107

4. References

(1) Borys, A. M. An Illustrated Guide to Schlenk Line Techniques. *Organometallics* **2023**, *42* (3), 182–196. https://doi.org/10.1021/ACS.ORGANOMET.2C00535/ASSET/IMAGES/LARGE/OM2C00535_0017.JPEG.

(2) Harris, R. K.; Becker, E. D.; Cabral De Menezes, S. M.; Goodfellow, R.; Granger, P. NMR Nomenclature. Nuclear Spin Properties and Conventions for Chemical Shifts(IUPAC Recommendations 2001). *Pure and Applied Chemistry* **2001**, *73* (11), 1795–1818. https://doi.org/10.1351/PAC200173111795.

(3) Harris, R. K.; Becker, E. D.; Menezes, S. M. C. de; Granger, P.; Hoffman, R. E.; Zilm, K. W. Further Conventions for NMR Shielding and Chemical Shifts (IUPAC Recommendations 2008). *Pure and Applied Chemistry* **2008**, *80* (1), 59–84. https://doi.org/10.1351/PAC200880010059.

(4) Chulsky, K.; Malahov, I.; Bawari, D.; Dobrovetsky, R. Metallomimetic Chemistry of a Cationic, Geometrically Constrained Phosphine in the Catalytic Hydrodefluorination and Amination of Ar−F Bonds. *Cite This: J. Am. Chem. Soc* **2023**, *145*, 3794. https://doi.org/10.1021/jacs.2c13318.

(5) Chambers, R. D.; Hall, C. W.; Hutchinson, J.; Millar, R. W. Polyhalogenated Heterocyclic Compounds. Part 42. 1 Fluorinated Nitrogen Heterocycles with Unusual Substitution Patterns F F F F O-O-O. *J. Chem. Soc., Perkin Trans. 1* **1998**.

(6) Xu, P.; López-Rojas, P.; Ritter, T. Radical Decarboxylative Carbometalation of Benzoic Acids: A Solution to Aromatic Decarboxylative Fluorination. *J Am Chem Soc* **2021**, *143* (14), 5349–5354. https://doi.org/10.1021/JACS.1C02490/ASSET/IMAGES/LARGE/JA1C02490_0004.JPEG.

(7) Zhang, J.; Zhao, X.; Yang, J.-D.; Cheng, J.-P. Diazaphospholene-Catalyzed Hydrodefluorination of Polyfluoroarenes with Phenylsilane via Concerted Nucleophilic Aromatic Substitution. *Cite This: J. Org. Chem* **2021**, *2022*, 300. https://doi.org/10.1021/acs.joc.1c02360.

(8) Yoshida, H.; Minabe, T.; Ohshita, J.; Kunai, A. Aminosilylation of Arynes with Aminosilanes: Synthesis of 2-Silylaniline Derivatives{. **2005**. https://doi.org/10.1039/b505615b.

(9) Lin, X.; Haimov, E.; Redko, B.; Vigalok, A. Selective Stepwise Arylation of Unprotected Peptides by PtIV Complexes. *Angewandte Chemie - International Edition* **2022**, *61* (29). https://doi.org/10.1002/ANIE.202205368.

(10) Chulsky, K.; Malahov, I.; Bawari, D.; Dobrovetsky, R. Metallomimetic Chemistry of a Cationic, Geometrically Constrained Phosphine in the Catalytic Hydrodefluorination and Amination of Ar-F Bonds. *J Am Chem Soc* **2023**, *145* (6), 3786–3794. https://doi.org/10.1021/JACS.2C13318/ASSET/IMAGES/LARGE/JA2C13318_0006.JPEG.

(11) Gutov, A. V; Rusanov, E. B.; Ryabitskii, A. B.; Chernega, A. N. Octafluoro-4,4 0-Bipyridine and Its Derivatives: Synthesis, Molecular and Crystal Structure. https://doi.org/10.1016/j.jfluchem.2009.11.022.

(12) Császár, P.; Pulay, P. Geometry Optimization by Direct Inversion in the Iterative Subspace. *J Mol Struct* **1984**, *114* (C), 31–34. https://doi.org/10.1016/S0022-2860(84)87198-7.

(13) Ahlrichs, R.; Bär, M.; Häser, M.; Horn, H.; Kölmel, C. Electronic Structure Calculations on Workstation Computers: The Program System Turbomole. *Chem Phys Lett* **1989**, *162* (3), 165–169. https://doi.org/10.1016/0009-2614(89)85118-8.

(14) Treutler, O.; Ahlrichs, R. Efficient Molecular Numerical Integration Schemes. *J Chem Phys* **1995**, *102* (1), 346–354. https://doi.org/10.1063/1.469408.

(15) Eichkorn, K.; Weigend, F.; Treutler, O.; Ahlrichs, R. Auxiliary Basis Sets for Main Row Atoms and Transition Metals and Their Use to Approximate Coulomb Potentials. *Theor Chem Acc* **1997**, *97* (1–4), 119–124. https://doi.org/10.1007/S002140050244/METRICS.

(16) Von Arnim, M.; Ahlrichs, R. Geometry Optimization in Generalized Natural Internal Coordinates. *J Chem Phys* **1999**, *111* (20), 9183–9190. https://doi.org/10.1063/1.479510.

(17) Deglmann, P.; Furche, F.; Ahlrichs, R. An Efficient Implementation of Second Analytical Derivatives for Density Functional Methods. *Chem Phys Lett* **2002**, *362* (5–6), 511–518. https://doi.org/10.1016/S0009-2614(02)01084-9.

(18) Deglmann, P.; May, K.; Furche, F.; Ahlrichs, R. Nuclear Second Analytical Derivative Calculations Using Auxiliary Basis Set Expansions. *Chem Phys Lett* **2004**, *384* (1–3), 103–107. https://doi.org/10.1016/J.CPLETT.2003.11.080.

(19) Baldes, A.; Weigend, F. Efficient Two-Component Self-Consistent Field Procedures and Gradients: Implementation in TURBOMOLE and Application To. *Mol Phys* **2013**, *111* (16–17), 2617–2624. https://doi.org/10.1080/00268976.2013.802037.

(20) Furche, F.; Ahlrichs, R.; Hättig, C.; Klopper, W.; Sierka, M.; Weigend, F. Turbomole. *Wiley Interdiscip Rev Comput Mol Sci* **2014**, *4* (2), 91–100. https://doi.org/10.1002/WCMS.1162.

(21) Deglmann, P.; Furche, F. Efficient Characterization of Stationary Points on Potential Energy Surfaces. *J Chem Phys* **2002**, *117* (21), 9535–9538. https://doi.org/10.1063/1.1523393.

(22) Eichkorn, K.; Treutler, O.; Öhm, H.; Häser, M.; Ahlrichs, R. Auxiliary Basis Sets to Approximate Coulomb Potentials. *Chem Phys Lett* **1995**, *240* (4), 283–290. https://doi.org/10.1016/0009-2614(95)00621-A.

(23) Schäfer, A.; Klamt, A.; Sattel, D.; Lohrenz, J. C. W.; Eckert, F. COSMO Implementation in TURBOMOLE: Extension of an Efficient Quantum Chemical Code towards Liquid Systems. *Physical Chemistry Chemical Physics* **2000**, *2* (10), 2187–2193. https://doi.org/10.1039/B000184H.

(24) Côté, J. F.; Brouillette, D.; Desnoyers, J. E.; Rouleau, J. F.; St-Arnaud, J. M.; Perron, G. Dielectric Constants of Acetonitrile, γ-Butyrolactone, Propylene Carbonate, and 1,2-Dimethoxyethane as a Function of Pressure and Temperature. *J Solution Chem* **1996**, *25* (12), 1163–1173. https://doi.org/10.1007/BF00972644.

(25) Ardura, D.; López, R.; Sordo, T. L. Relative Gibbs Energies in Solution through Continuum Models: Effect of the Loss of Translational Degrees of Freedom in Bimolecular Reactions on Gibbs Energy Barriers. *J Phys Chem B* **2005**, *109* (49), 23618–23623. https://doi.org/10.1021/JP0540499.

(26) Dub, P. A.; Poli, R. A Computational Study of Solution Equilibria of Platinum-Based Ethylene Hydroamination Catalytic Species Including Solvation and Counterion Effects: Proper Treatment of the Free Energy of Solvation. *J Mol Catal A Chem* **2010**, *324* (1–2), 89–96. https://doi.org/10.1016/J.MOLCATA.2010.03.003.

(27) Leung, B. O.; Reid, D. L.; Armstrong, D. A.; Rauk, A. Entropies in Solution from Entropies in the Gas Phase. *Journal of Physical Chemistry A* **2004**, *108* (14), 2720–2725. https://doi.org/10.1021/JP030265A/ASSET/IMAGES/MEDIUM/JP030265AE00022.GIF.

(28) Grimme, S.; Antony, J.; Ehrlich, S.; Krieg, H. A Consistent and Accurate Ab Initio Parametrization of Density Functional Dispersion Correction (DFT-D) for the 94 Elements H-Pu. *Journal of Chemical Physics* **2010**, *132* (15), 154104. https://doi.org/10.1063/1.3382344/926936.

(29) Grimme, S.; Ehrlich, S.; Goerigk, L. Effect of the Damping Function in Dispersion Corrected Density Functional Theory. *J Comput Chem* **2011**, *32* (7), 1456–1465. https://doi.org/10.1002/JCC.21759.

(30) SUENAGA, M. Facio: New Computational Chemistry Environment for PC GAMESS. *Journal of Computer Chemistry, Japan* **2005**, *4* (1), 25–32. https://doi.org/10.2477/JCCJ.4.25.

(31) *Jmol: an open-source Java viewer for chemical structures in 3D*. https://jmol.sourceforge.net/ (accessed 2023-09-21).
